# Integrative Annotation of 21,037 Human Genes Validated by Full-Length cDNA Clones

**DOI:** 10.1371/journal.pbio.0020162

**Published:** 2004-04-20

**Authors:** Tadashi Imanishi, Takeshi Itoh, Yutaka Suzuki, Claire O'Donovan, Satoshi Fukuchi, Kanako O Koyanagi, Roberto A Barrero, Takuro Tamura, Yumi Yamaguchi-Kabata, Motohiko Tanino, Kei Yura, Satoru Miyazaki, Kazuho Ikeo, Keiichi Homma, Arek Kasprzyk, Tetsuo Nishikawa, Mika Hirakawa, Jean Thierry-Mieg, Danielle Thierry-Mieg, Jennifer Ashurst, Libin Jia, Mitsuteru Nakao, Michael A Thomas, Nicola Mulder, Youla Karavidopoulou, Lihua Jin, Sangsoo Kim, Tomohiro Yasuda, Boris Lenhard, Eric Eveno, Yoshiyuki Suzuki, Chisato Yamasaki, Jun-ichi Takeda, Craig Gough, Phillip Hilton, Yasuyuki Fujii, Hiroaki Sakai, Susumu Tanaka, Clara Amid, Matthew Bellgard, Maria de Fatima Bonaldo, Hidemasa Bono, Susan K Bromberg, Anthony J Brookes, Elspeth Bruford, Piero Carninci, Claude Chelala, Christine Couillault, Sandro J. de Souza, Marie-Anne Debily, Marie-Dominique Devignes, Inna Dubchak, Toshinori Endo, Anne Estreicher, Eduardo Eyras, Kaoru Fukami-Kobayashi, Gopal R. Gopinath, Esther Graudens, Yoonsoo Hahn, Michael Han, Ze-Guang Han, Kousuke Hanada, Hideki Hanaoka, Erimi Harada, Katsuyuki Hashimoto, Ursula Hinz, Momoki Hirai, Teruyoshi Hishiki, Ian Hopkinson, Sandrine Imbeaud, Hidetoshi Inoko, Alexander Kanapin, Yayoi Kaneko, Takeya Kasukawa, Janet Kelso, Paul Kersey, Reiko Kikuno, Kouichi Kimura, Bernhard Korn, Vladimir Kuryshev, Izabela Makalowska, Takashi Makino, Shuhei Mano, Regine Mariage-Samson, Jun Mashima, Hideo Matsuda, Hans-Werner Mewes, Shinsei Minoshima, Keiichi Nagai, Hideki Nagasaki, Naoki Nagata, Rajni Nigam, Osamu Ogasawara, Osamu Ohara, Masafumi Ohtsubo, Norihiro Okada, Toshihisa Okido, Satoshi Oota, Motonori Ota, Toshio Ota, Tetsuji Otsuki, Dominique Piatier-Tonneau, Annemarie Poustka, Shuang-Xi Ren, Naruya Saitou, Katsunaga Sakai, Shigetaka Sakamoto, Ryuichi Sakate, Ingo Schupp, Florence Servant, Stephen Sherry, Rie Shiba, Nobuyoshi Shimizu, Mary Shimoyama, Andrew J Simpson, Bento Soares, Charles Steward, Makiko Suwa, Mami Suzuki, Aiko Takahashi, Gen Tamiya, Hiroshi Tanaka, Todd Taylor, Joseph D Terwilliger, Per Unneberg, Vamsi Veeramachaneni, Shinya Watanabe, Laurens Wilming, Norikazu Yasuda, Hyang-Sook Yoo, Marvin Stodolsky, Wojciech Makalowski, Mitiko Go, Kenta Nakai, Toshihisa Takagi, Minoru Kanehisa, Yoshiyuki Sakaki, John Quackenbush, Yasushi Okazaki, Yoshihide Hayashizaki, Winston Hide, Ranajit Chakraborty, Ken Nishikawa, Hideaki Sugawara, Yoshio Tateno, Zhu Chen, Michio Oishi, Peter Tonellato, Rolf Apweiler, Kousaku Okubo, Lukas Wagner, Stefan Wiemann, Robert L Strausberg, Takao Isogai, Charles Auffray, Nobuo Nomura, Takashi Gojobori, Sumio Sugano

**Affiliations:** **1**Integrated Database Group, Biological Information Research Center, National Institute of Advanced Industrial Science and TechnologyTokyoJapan; **2**Bioinformatics Laboratory, Genome Research Department, National Institute of Agrobiological SciencesIbarakiJapan; **3**Human Genome Center, The Institute of Medical Science, The University of TokyoTokyoJapan; **4**EMBL Outstation—European Bioinformatics Institute, Wellcome Trust Genome CampusCambridgeUnited Kingdom; **5**Center for Information Biology and DNA Data Bank of Japan, National Institute of GeneticsShizuokaJapan; **6**Nara Institute of Science and TechnologyNaraJapan; **7**Integrated Database Group, Japan Biological Information Research Center, Japan Biological Informatics ConsortiumTokyoJapan; **8**BITS CompanyShizuokaJapan; **9**Quantum Bioinformatics Group, Center for Promotion of Computational Science and Engineering, Japan Atomic Energy Research InstituteKyotoJapan; **10**Reverse Proteomics Research InstituteChibaJapan; **11**Central Research Laboratory, HitachiTokyoJapan; **12**Bioinformatics Center, Institute for Chemical Research, Kyoto UniversityKyotoJapan; **13**National Center for Biotechnology Information, National Library of Medicine, National Institutes of HealthBethesda, MarylandUnited States of America; **14**Centre National de la Recherche Scientifique (CNRS), Laboratoire de Physique MathematiqueMontpellierFrance; **15**The Wellcome Trust Sanger Institute, Wellcome Trust Genome CampusCambridgeUnited Kingdom; **16**National Cancer Institute, National Institutes of HealthBethesda, MarylandUnited States of America; **17**Department of Biological Sciences, Idaho State UniversityPocatello, IdahoUnited States of America; **18**Korea Research Institute of Bioscience and BiotechnologyTaejeonKorea; **19**Center for Genomics and Bioinformatics, Karolinska InstitutetStockholmSweden; **20**Genexpress—CNRS—Functional Genomics and Systemic Biology for HealthVillejuif CedexFrance; **21**Sino-French Laboratory in Life Sciences and GenomicsShanghaiChina; **22**Tokyo Research Laboratories, Kyowa Hakko Kogyo CompanyTokyoJapan; **23**MIPS—Institute for Bioinformatics, GSF—National Research Center for Environment and HealthNeuherbergGermany; **24**Centre for Bioinformatics and Biological Computing, School of Information Technology, Murdoch UniversityMurdoch, Western AustraliaAustralia; **25**Medical Education and Biomedical Research Facility, University of IowaIowa City, IowaUnited States of America; **26**Genome Exploration Research Group, RIKEN Genomic Sciences Center, RIKEN Yokohama InstituteKanagawaJapan; **27**Medical College of Wisconsin, MilwaukeeWisconsinUnited States of America; **28**HUGO Gene Nomenclature Committee, University College LondonLondonUnited Kingdom; **29**Genome Science Laboratory, RIKENSaitamaJapan; **30**Ludwig Institute of Cancer ResearchSao PauloBrazil; **31**CNRSVandoeuvre les NancyFrance; **32**Lawrence Berkeley National Laboratory, BerkeleyCaliforniaUnited States of America; **33**Department of Bioinformatics, Medical Research Institute, Tokyo Medical and Dental UniversityTokyoJapan; **34**Swiss Institute of BioinformaticsGenevaSwitzerland; **35**Bioresource Information Division, RIKEN BioResource Center, RIKEN Tsukuba InstituteIbarakiJapan; **36**Genome Knowledgebase, Cold Spring Harbor LaboratoryCold Spring Harbor, New YorkUnited States of America; **37**Chinese National Human Genome Center at ShanghaiShanghaiChina; **38**Division of Genetic Resources, National Institute of Infectious DiseasesTokyoJapan; **39**Graduate School of Frontier Sciences, Department of Integrated Biosciences, University of TokyoChibaJapan; **40**Functional Genomics Group, Biological Information Research Center, National Institute of Advanced Industrial Science and TechnologyTokyoJapan; **41**Department of Primary Care and Population Sciences, Royal Free University College Medical School, University College LondonLondonUnited Kingdom; **42**Clinical and Molecular Genetics Unit, The Institute of Child HealthLondonUnited Kingdom; **43**Department of Genetic Information, Division of Molecular Life Science, School of Medicine, Tokai UniversityKanagawaJapan; **44**South African National Bioinformatics Institute, University of the Western CapeBellvilleSouth Africa; **45**Kazusa DNA Research InstituteChibaJapan; **46**RZPD Resource Center for Genome ResearchHeidelbergGermany; **47**Molecular Genome Analysis, German Cancer Research Center-DKFZHeidelbergGermany; **48**Pennsylvania State UniversityUniversity Park, PennsylvaniaUnited States of America; **49**Department of Bioinformatic Engineering, Graduate School of Information Science and Technology, Osaka UniversityOsakaJapan; **50**Medical Photobiology Department, Photon Medical Research Center, Hamamatsu University School of MedicineShizuokaJapan; **51**Computational Biology Research Center, National Institute of Advanced Industrial Science and TechnologyTokyoJapan; **52**Department of Molecular Biology, Keio University School of MedicineTokyoJapan; **53**Department of Biological Sciences, Graduate School of Bioscience and Biotechnology, Tokyo Institute of TechnologyKanagawaJapan; **54**Global Scientific Information and Computing Center, Tokyo Institute of TechnologyTokyoJapan; **55**Molecular Biology Laboratory, Medicinal Research Laboratories, Taisho Pharmaceutical CompanySaitamaJapan; **56**Department of Population Genetics, National Institute of GeneticsShizuokaJapan; **57**Human Genome Research Group, Genomic Sciences Center, RIKEN Yokohama InstituteKanagawaJapan; **58**Columbia University and Columbia Genome CenterNew York, New YorkUnited States of America; **59**Department of Biotechnology, Royal Institute of TechnologyStockholmSweden; **60**Biology Division and Genome Task Group, Office of Biological and Environmental Research, United States Department of EnergyWashington, D.CUnited States of America; **61**Faculty of Bio-Science, Nagahama Institute of Bio-Science and TechnologyShigaJapan; **62**Institute for Genomic ResearchRockville, MarylandUnited States of America; **63**Center for Genome Information, Department of Environmental Health, University of CincinnatiCincinnati, OhioUnited States of America; **64**State Key Laboratory of Medical Genomics, Shanghai Institute of Hematology, Rui-Jin Hospital, Shanghai Second Medical UniversityShanghaiChina; **65**PointOne SystemsWauwatosa, WisconsinUnited States of America; **66**Graduate School of Life and Environmental Sciences, University of TsukubaIbarakiJapan; **67**Department of Genetics, Graduate University for Advanced StudiesShizuokaJapan; **68**Department of Medical Genome Sciences, Graduate School of Frontier Sciences, University of TokyoTokyoJapan

## Abstract

The human genome sequence defines our inherent biological potential; the realization of the biology encoded therein requires knowledge of the function of each gene. Currently, our knowledge in this area is still limited. Several lines of investigation have been used to elucidate the structure and function of the genes in the human genome. Even so, gene prediction remains a difficult task, as the varieties of transcripts of a gene may vary to a great extent. We thus performed an exhaustive integrative characterization of 41,118 full-length cDNAs that capture the gene transcripts as complete functional cassettes, providing an unequivocal report of structural and functional diversity at the gene level. Our international collaboration has validated 21,037 human gene candidates by analysis of high-quality full-length cDNA clones through curation using unified criteria. This led to the identification of 5,155 new gene candidates. It also manifested the most reliable way to control the quality of the cDNA clones. We have developed a human gene database, called the H-Invitational Database (H-InvDB; http://www.h-invitational.jp/). It provides the following: integrative annotation of human genes, description of gene structures, details of novel alternative splicing isoforms, non-protein-coding RNAs, functional domains, subcellular localizations, metabolic pathways, predictions of protein three-dimensional structure, mapping of known single nucleotide polymorphisms (SNPs), identification of polymorphic microsatellite repeats within human genes, and comparative results with mouse full-length cDNAs. The H-InvDB analysis has shown that up to 4% of the human genome sequence (National Center for Biotechnology Information build 34 assembly) may contain misassembled or missing regions. We found that 6.5% of the human gene candidates (1,377 loci) did not have a good protein-coding open reading frame, of which 296 loci are strong candidates for non-protein-coding RNA genes. In addition, among 72,027 uniquely mapped SNPs and insertions/deletions localized within human genes, 13,215 nonsynonymous SNPs, 315 nonsense SNPs, and 452 indels occurred in coding regions. Together with 25 polymorphic microsatellite repeats present in coding regions, they may alter protein structure, causing phenotypic effects or resulting in disease. The H-InvDB platform represents a substantial contribution to resources needed for the exploration of human biology and pathology.

## Abstract 2

An international team has systematically validated and annotated just over 21,000 human genes using full-length cDNA, thereby providing a valuable new resource for the human genetics community

## Introduction

The draft sequences of the human, mouse, and rat genomes are already available (Lander et al. 2001; Marshall 2001; Venter et al. 2001; Waterston et al. 2002). The next challenge comes in the understanding of basic human molecular biology through interpretation of the human genome. To display biological data optimally we must first characterize the genome in terms of not only its structure but also function and diversity. It is of immediate interest to identify factors involved in the developmental process of organisms, non-protein-coding functional RNAs, the regulatory network of gene expression within tissues and its governance over states of health, and protein–gene and protein–protein interactions. In doing so, we must integrate this information in an easily accessible and intuitive format. The human genome may encode only 30,000 to 40,000 genes (Lander et al. 2001; Venter et al. 2001), suggesting that complex interdependent gene regulation mechanisms exist to account for the complex gene networks that differentiate humans from lower-order organisms. In organisms with small genomes, it is relatively straightforward to use direct computational prediction based upon genomic sequence to identify most genes by their long open reading frames (ORFs). However, computational gene prediction from the genomic sequence of organisms with short exons and long introns can be somewhat error-prone (Ashburner 2000; Reese et al. 2000; Lander et al. 2001).

Previous efforts to catalogue the human transcriptome were based on expressed sequence tags (ESTs) used for the identification of new genes (Adams et al. 1991; Auffray et al. 1995; Houlgatte et al. 1995), chromosomal assignment of genes (Gieser and Swaroop 1992; Khan et al. 1992; Camargo et al. 2001), prediction of genes (Nomura et al. 1994), and assessment of gene expression (Okubo et al. 1992). Recently, Camargo et al. (2001) generated a large collection of ORF ESTs, and Saha et al. (2002) conducted a large-scale serial analysis of gene expression patterns to identify novel human genes. The availability of human full-length transcripts from many large-scale sequencing projects (Nomura et al. 1994; Nagase et al. 2001; Wiemann et al. 2001; Yudate 2001; Kikuno et al. 2002;Strausberg et al. 2002) has provided a unique opportunity for the comprehensive evaluation of the human transcriptome through the annotation of a variety of RNA transcripts. Protein-coding and non-protein-coding sequences, alternative splicing (AS) variants, and sense–antisense RNA pairs could all be functionally identified. We thus designed an international collaborative project to establish an integrative annotation database of 41,118 human full-length cDNAs (FLcDNAs). These cDNAs were collected from six high-throughput sequencing projects and evaluated at the first international jamboree, entitled the Human Full-length cDNA Annotation Invitational (H-Invitational or H-Inv) (Cyranoski 2002). This event was held in Tokyo, Japan, and took place from August 25 to September 3, 2002.

Efforts which have been made in the same area as the H-Inv annotation work include the Functional Annotation of Mouse (FANTOM) project (Kawai et al. 2001; Bono et al. 2002; Okazaki et al. 2002), Flybase (GOC 2001), and the RIKEN *Arabidopsis* full-length cDNA project (Seki et al. 2002). In our own project, great effort has been taken at all levels, not only in the annotation of the cDNAs but also in the way the data can be viewed and queried. These aspects, along with the applications of our research to disease research, distinguish our project from other similar projects.

This manuscript provides the first report by the H-Inv consortium, showing some of the discoveries made so far and introducing our new database of the human transcriptome. It is hoped that this will be the first in a long line of publications announcing discoveries made by the H-Inv consortium. Here we describe results from our integrative annotation in four major areas: mapping the transcriptome onto the human genome, functional annotation, polymorphism in the transcriptome, and evolution of the human transcriptome. We then introduce our new database of the human transcriptome, the H-Invitational Database (H-InvDB; http://www.h-invitational.jp), which stores all annotation results by the consortium. Free and unrestricted access to the H-Inv annotation work is available through the database. Finally, we summarize our most important findings thus far in the H-Inv project in Concluding Remarks.

## Results/Discussion

### Mapping the Transcriptome onto the Human Genome

#### Construction of the nonredundant human FLcDNA database

We present the first experimentally validated nonredundant transcriptome of human FLcDNAs produced by six high-throughput cDNA sequencing projects (Ota et al. 1997, 2004; Strausberg et al. 1999; Hu et al. 2000; Wiemann et al. 2001; Yudate 2001; Kikuno et al. 2002) as of July 15, 2002. The dataset consists of 41,118 cDNAs (H-Inv cDNAs) that were derived from 184 diverse cell types and tissues (see Dataset S1). The number of clones, the number of libraries, major tissue origins, methods, and URLs of cDNA clones for each cDNA project are summarized in Table 1. H-Inv cDNAs include 8,324 cDNAs recently identified by the Full-Length Long Japan (FLJ) project. The FLJ clones represent about half of the H-Inv cDNAs (Table 1). The policies for library selection and the results of initial analysis of the constituent projects were reported by the participants themselves: the Chinese National Human Genome Center (CHGC) (Hu et al. 2000), the Deutsches Krebsforschungszentrum (DKFZ/MIPS) (Wiemann et al. 2001), the Institute of Medical Science at the University of Tokyo (IMSUT) (Suzuki et al. 1997 ; Ota et al. 2004), the Kazusa cDNA sequence project of the Kazusa DNA Research Institute (KDRI) (Hirosawa et al. 1999; Nagase et al. 1999; Suyama et al. 1999; Kikuno et al. 2002), the Helix Research Institute (HRI) (Yudate et al. 2001), and the Mammalian Gene Collection (MGC) (Strausberg et al. 1999; Moonen et al. 2002), as well as FLJ mentioned earlier (Ota et al. 2004). The variation in tissue origins for library construction among these six groups resulted in rare occurrences of sequence redundancy among the collections. In a recent study, the FLJ project has described the complete sequencing and characterization of 21,243 human cDNAs (Ota et al. 2004). On the other hand, the H-Inv project characterized cDNAs from this project and six high-throughput cDNA producers by using a different suite of computational analysis techniques and an alternative system of functional annotation.

**Table 1:**
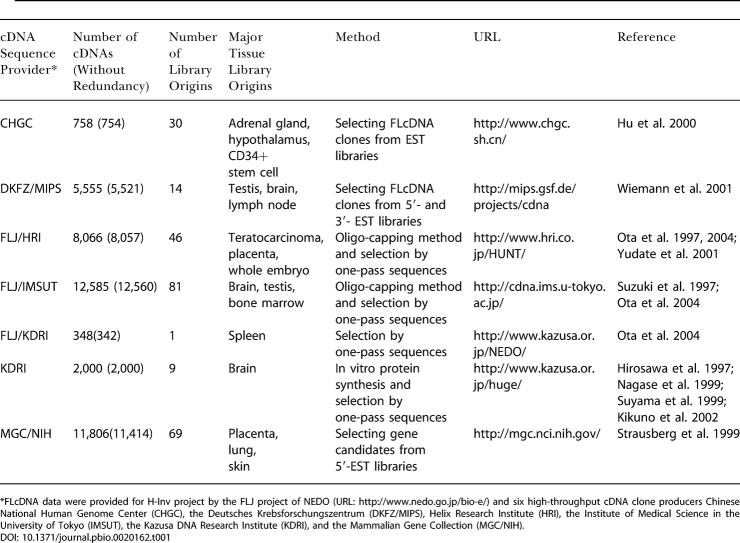
Summary of cDNA Resources

*FLcDNA data were provided for H-Inv project by the FLJ project of NEDO (URL: http://www.nedo.go.jp/bio-e/) and six high-throughput cDNA clone producers Chinese National Human Genome Center (CHGC), the Deutsches Krebsforschungszentrum (DKFZ/MIPS), Helix Research Institute (HRI), the Institute of Medical Science in the University of Tokyo (IMSUT), the Kazusa DNA Research Institute (KDRI), and the Mammalian Gene Collection (MGC/NIH)

**Figure 1:**
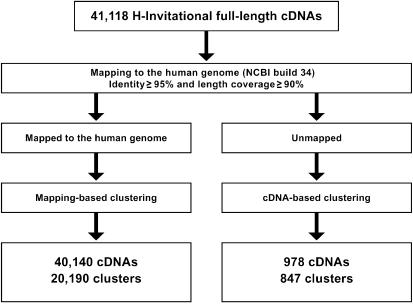
Procedure for Mapping and Clustering the H-Inv cDNAs The cDNAs were mapped to the genome and clustered into loci. The remaining unmapped cDNAs were clustered based upon the grouping of significantly similar cDNAs.

The 41,118 H-Inv cDNAs were mapped on to the human genome, and 40,140 were considered successfully aligned. The alignment criterion was that a cDNA was only aligned if it had both 95% identity and 90% length coverage against the genome (Figure 1). The mean identity of all the alignments between 40,140 mapped cDNAs and genomic sequences was 99.6 %, and the mean coverage against the genomic sequence was 99.6%. In some cases, terminal exons were aligned with low identity or low coverage. For example, 89% of internal exons have identity of 99.8% or higher, while only 78% and 50% of the first and last exons do, respectively. These alignments with low identity or low coverage seemed to be caused by the unsuccessful alignments of the repetitive sequences found in UTR regions and the misalignments of 3′ terminal poly-A sequences. Although better alignments could be obtained for these sequences by improving the mapping procedure, we concluded that the quality of the FLcDNAs was high overall.

Due to redundancy and AS within the human transcriptome, these 40,140 cDNAs were clustered to 20,190 loci (H-Inv loci). For the remaining 978 unmapped cDNAs, we conducted cDNA-based clustering, which yielded 847 clusters. The clusters created had an average of 2.0 cDNAs per locus (Table 2). The average was only 1.2 for unmapped clusters, probably because many of these genes are encoded by heterochromatic regions of the human genome and show limited levels of gene expression. The gene density for each chromosome varied from 0.6 to 19.0 genes/Mb, with an average of 6.5 genes/Mb. This distribution of genes over the genome is far from random. This biased gene localization concurs with the gene density on chromosomes found in similar previous reports (Lander et al. 2001; Venter et al. 2001). This indicates that the sampled cDNAs are unbiased with respect to chromosomal location. Most cDNAs were mapped only at a single position on the human genome. However, 1,682 cDNAs could be mapped at multiple positions (with mean values of 98.2% identity and 98.1% coverage). The multiple matching may be caused by either recent gene duplication events or artificial duplication of the human genome caused by misassembled contigs. In our study we have selected only the “best” loci for the cDNAs (see Materials and Methods for details).

**Table 2:**
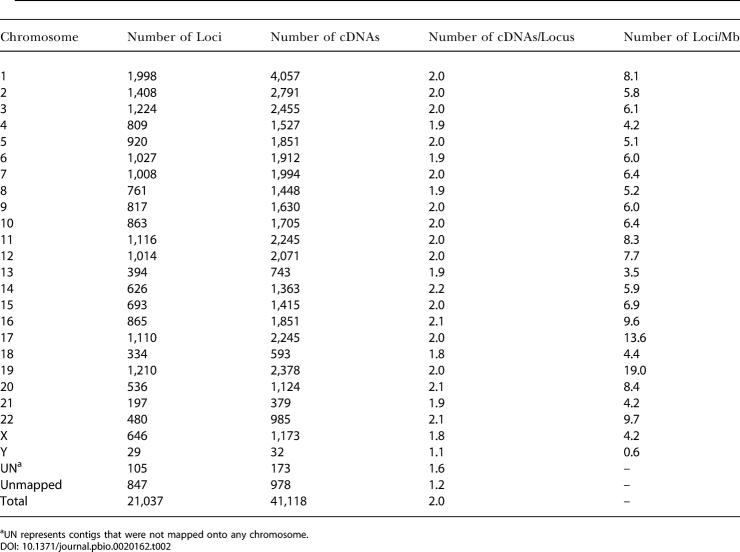
The Clustering Results of Human FLcDNAs onto the Human Genome

^a^UN represents contigs that were not mapped onto any chromosome

In total, 21,037 clusters (20,190 mapped and 847 unmapped) were identified and entered into the H-InvDB. We assigned H-Inv cluster IDs (e.g., HIX0000001) to the clusters and H-Inv cDNA IDs (e.g., HIT000000001) to all curated cDNAs. A representative sequence was selected from each cluster and used for further analyses and annotation.

#### Comparison of the mapped H-Inv cDNAs with other annotated datasets

**Figure 2:**
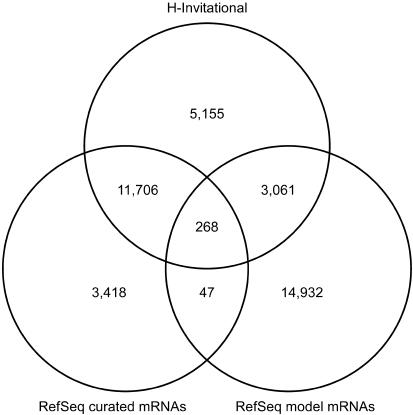
A Comparison of the Mapped H-Inv FLcDNAs and the RefSeq mRNAs The mapped H-Inv cDNAs, the RefSeq curated mRNAs (accession prefixes NM and NR), and the RefSeq model mRNAs (accession prefixes XM and XR) provided by the genome annotation process were clustered based on the genome position. The numbers of loci that were identified by clustering are shown.

In order to evaluate the H-Inv dataset, we compared all of the mapped H-Inv cDNAs with the Reference Sequence Collection (RefSeq) mRNA database (Pruitt and Maglott 2001) (Figure 2). The RefSeq mRNA database consists of two types of datasets. These are the curated mRNAs (accession prefix NM and NR) and the model mRNAs that are provided through automated processing of the genome annotation (accession prefix XM and XR).

From the comparison, we found that 5,155 (26%) of the H-Inv loci had no counterparts and were unique to the H-Inv. All of these 5,155 loci are candidates for new human genes, although non-protein-coding RNAs (ncRNAs) (25%), hypothetical proteins with ORFs less than 150 amino acids (55%), and singletons (91%) were enriched in this category. In fact, 1,340 of these H-Inv-unique loci were questionable and require validation by further experiments because they consist of only single exons, and the 3′ termini of these loci align with genomic poly-A sequences. This feature suggests internal poly-A priming although some occurrences might be bona fide genes. The most reliable set of newly identified human genes in our dataset is composed of 1,054 protein-coding and 179 non-protein-coding genes that have multiple exons. Therefore, at least 6.1% (1,233/20,190) of the H-Inv loci could be used to newly validate loci that the RefSeq datasets do not presently cover. These genes are possibly less expressed since the proportion of singletons (H-Inv loci consisting of a single H-Inv cDNA) was high (84%).

On the other hand, 78% (11,974/15,439) of the curated RefSeq mRNAs were covered by the H-Inv cDNAs. These figures suggest that further extensive sequencing of FLcDNA clones will be required in order to cover the entire human gene set. Nonetheless, this effort provides a systematic approach using the H-Inv cDNAs, even though a portion of the cDNAs have already been utilized in the RefSeq datasets.

It is noteworthy that H-Inv cDNAs overlapped 3,061 (17%) of RefSeq model mRNAs, supporting this proportion of the hypothetical RefSeq sequences. These newly confirmed 3,061 loci have a mean number of exons greater than RefSeq model mRNAs that were not confirmed, but smaller than RefSeq curated mRNAs. The overlap between H-Inv cDNAs and RefSeq model mRNAs was smaller than that between H-Inv cDNAs and RefSeq curated mRNAs. This suggests that the genes predicted from genome annotation may tend to be less expressed than RefSeq curated genes, or that some may be artifacts. All these results highlight the great importance of comprehensive collections of analyzed FLcDNAs for validating gene prediction from genome sequences. This may be especially true for higher organisms such as humans.

#### Incomplete parts of the human genome sequences

The existence of 978 unmapped cDNAs (847 clusters) suggests that the human genome sequence (National Center for Biotechnolgy Information [NCBI] build 34 assembly) is not yet complete. The evidence supporting this statement is twofold. First, most of those unmapped cDNAs could be partially mapped to the human genome. Using BLAST, 906 of the unmapped cDNAs (corresponding to 786 clusters) showed at least one sequence match to the human genome with a bit score higher than 100. Second, most of the cDNAs could be mapped unambiguously to the mouse genome sequences. A total of 907 unmapped cDNAs (779 clusters; 92%) could be mapped to the mouse genome with coverage of 90% or higher. If we adopted less stringent requirements, more cDNAs could be mapped to the mouse genome. The rest might be less conserved genes, genes in unfinished sections of the mouse genome, or genes that were lost in the mouse genome. Based on these observations, we conclude that the human genome sequence is not yet complete, leaving some portions to be sequenced or reassembled.

The proportion of the genome that is incomplete is estimated to be 3.7%–4.0%. The figure of 4.0% is based upon the proportion of H-Inv cDNA clusters that could not be mapped to the genome (847/21,037), while the 3.7% estimate is based on both H-Inv cDNAs and RefSeq sequences (only NMs). This statistic indicates that a minimum of one out of every 25–27 clusters appears to be unrepresented in the current human genome dataset, in its full form. Possible reasons for this include unsequenced regions on the human genome and regions where an error may have occurred during sequence assembly. If this is the case, this lends support to the use of cDNA mapping to facilitate the completion of whole genome sequences (Kent and Haussler 2001). For example, we can predict the arrangement of contigs based on the order of mapped exons. In addition we can use the sequences of unmapped exons to search for those clones that contain unsequenced parts of the genome. The mapping results of partially mapped cDNAs are thus quite useful.

#### Primary structure of genes on the human genome

Using the H-Inv cDNAs, the precise structures of many human genes could be identified based on the results of our cDNA mapping (Table S1). The median length of last exons (786 bp) was found to be longer than that of other exons, and the median length of first introns (3,152 bp) longer than that of other introns. These observed characteristics of human gene structures concur with the previous work using much smaller datasets (Hawkins 1988; Maroni 1996; Kriventseva and Gelfand 1999).

In the human genome, 50% of the sequence is occupied by repetitive elements (Lander et al. 2001). Repetitive elements were previously regarded by many as simply “junk” DNA. However, the contribution of these repetitive stretches to genome evolution has been suggested in recent works (Makalowski 2000; Deininger and Batzer 2002; Sorek et al. 2002; Lorenc and Makalowski 2003). The 21,037 loci of representative cDNAs were searched for repetitive elements using the RepeatMasker program. RepeatMasker indicated that 9,818 (47%) of the H-Inv cDNAs, including 5,442 coding hypothetical proteins, contained repetitive sequences. The existence of *Alu* repeats in 5% of human cDNAs was reported previously (Yulug et al. 1995). Our results revealed a significant number of repetitive sequences including *Alu* in the human transcriptome. Among them, 1,866 cDNAs overlapped repetitive sequences in their ORFs. Moreover, 554 of 1,866 cDNAs had repetitive sequences contained completely within their ORFs, including 81 cDNAs that were identical or similar to known proteins. This may indicate the involvement of repetitive elements in human transcriptome evolution, as suggested by the presence of Alu repeats in AS exons (Sorek et al. 2002) and the contribution to protein variability by repetitive elements in protein-coding regions (Makalowski 2000). We detected 2,254 and 5,427 cDNAs containing repetitive sequences in their 5′ UTR and 3′ UTR, respectively. The positioning of the repetitive elements suggests they play a regulatory role in the control of gene expression (Deininger and Batzer 2002) (see Table S1 or the H-InvDB for details).

#### AS transcripts

We wished to investigate the extent to which the functional diversity of the human proteome is affected by AS. In order to do this, we searched for potential AS isoforms in 7,874 loci that were supported by at least two H-Inv cDNAs. We examined whether or not these cDNAs represented mutually exclusive AS isoforms, using a combination of computational methods and human curation (see Materials and Methods). All AS isoforms that were supported independently by both methods were defined as the H-Inv AS dataset. Our analysis showed that 3,181 loci (40 % of the 7,874 loci) encoded 8,553 AS isoforms expressing a total of 18,612 AS exons. On average, 2.7 AS isoforms per locus were identified in these AS-containing loci. This figure represents half of the AS isoforms predicted by another group (Lander et al. 2001). Our result highlights the degree to which full-length sequencing of redundant clones is necessary when characterizing the complete human transcriptome. The relative positions of AS exons on the loci varied: 4,383 isoforms comprising 1,538 loci were 5′ terminal AS variants; 5,678 isoforms comprising 1,979 loci were internal AS variants; and 2,524 isoforms comprising 921 loci were 3′ terminal AS variants.

The AS isoforms found in the H-Inv AS dataset have strikingly diverse functions. Motifs are found over a wide range of protein sequences. For certain types of subcellular targeting signals, such as signal peptides, position within the entire protein sequence appears crucial. A total of 3,020 (35 %) AS isoforms contained AS exons that overlapped protein-coding sequences. 1,660 out of 3,020 AS isoforms (55%) harbored AS exons that encoded functional motifs. Additionally, 1,475 loci encoded AS isoforms that had different subcellular localization signals, and 680 loci had AS isoforms that had different transmembrane domains. These results suggest marked functional differentiation between the varying isoforms. If this is the case, it would appear that AS contributes significantly to the functional diversity of the human proteome.

As the coverage of the human transcriptome by H-Inv cDNAs is incomplete, it would be misleading to conjecture that our dataset comprehensively includes all AS transcripts from every human gene. However, the current collection is a robust characterization of the existing functional diversity of the human proteome, and it represents a valuable resource of full-length clones for the characterization of experimentally determined AS isoforms.

In the cases where three-dimensional (3D) structures could be assigned to H-Inv cDNA protein products, we have examined the possible impact of AS rearrangements on the 3D structure. Our analysis was performed using the Genomes TO Protein structures and functions database (GTOP) (Kawabata et al. 2002). We found that some of the sequence regions in which internal exons vary between different isoforms contained regions encoding SCOP domains (Lo Conte et al. 2000). This discovery allowed us to perform a simple analysis of the structural effects of AS. Our analysis of the SCOP domain assignments revealed that the loci displaying AS are much more likely to contain class c (β–α–β units, α/β) SCOP domains than class d (segregated α and β regions, α+β) or class g (small) domains.

**Figure 3:**
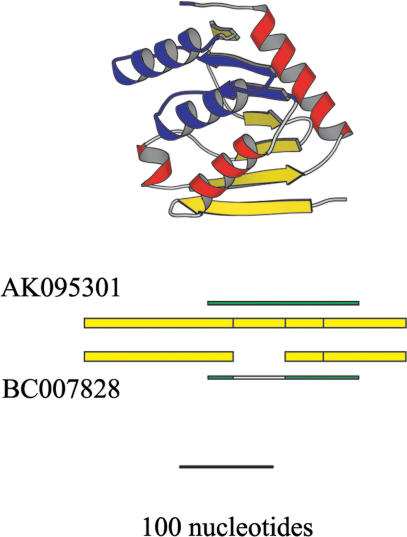
An Example of Different Structures Encoded by AS Variants Exons are presented from the 5′ end, with those shared by AS variants aligned vertically. The AS variants, with accession numbers AK095301 and BC007828, are aligned to the SCOP domain d.136.1.1 and corresponding PDB structure 1byr. Helices and beta sheets are red and yellow, respectively. Green bars indicate regions aligned to the PDB structure, while open rectangles represent gaps in the alignments. AK095301 is aligned to the entire PDB structure shown, while BC007828 is lacking the alignment to the purple segment of the structure.

An example of exon differences between AS isoforms is presented in Figure 3. The structures shown are those of proteins in the Brookhaven Protein Data Bank (PDB) (Berman et al. 2000) to which the amino acid sequences of the corresponding AS isoforms are aligned. Segments of the AS isoform sequences that are not aligned with the corresponding 3D structure are shown in purple. Figure 3 demonstrates that exon differences resulting from AS sometimes give rise to significant alternations in 3D structure.

### Functional Annotation

We predicted the ORFs of 41,118 H-Inv cDNA sequences using a computational approach (see Figure S1), of which 39,091 (95.1%) were protein coding and the remaining 2,027 (4.9%) were non-protein-coding. Since the structures and functions of protein products from AS isoforms are expected to be basically similar, we selected a “representative transcript” from each of the loci (see Figure S2). Then we identified 19,660 protein-coding and 1,377 non-protein-coding loci (Table 3). Human curation suggested that a total of 86 protein-coding transcripts should be deemed questionable transcripts. Once identified as dubious these sequences were excluded from further analysis. The remaining representatives from the 19,574 protein-coding loci were used to define a set of human proteins (H-Inv proteins). The tentative functions of the H-Inv proteins were predicted by computational methods. Following computational predictions was human curation.

**Table 3:**
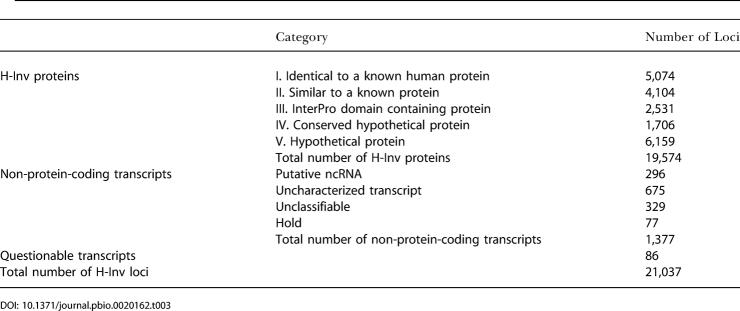
Statistics Obtained from the Functional Annotation Results

**Figure 4:**
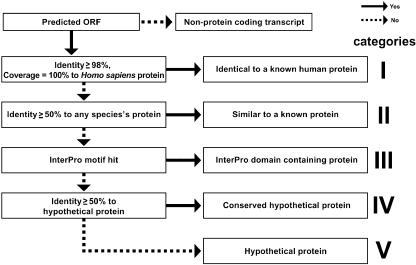
Schematic Diagram of Human Curation for H-Inv Proteins The diagram illustrates the human curation pipeline to classify H-Inv proteins into five similarity categories; Category I , II, III, IV, and V proteins.

After determination of the H-Inv proteins, we performed a standardized functional annotation as illustrated in Figure 4, during which we assigned the most suitable data source ID to each H-Inv protein based on the results of similarity search and InterProScan. We classified the 19,574 H-Inv proteins according to the levels of the sequence similarity. Using a system developed for the human cDNA annotation (see Figure S2), we classified the H-Inv proteins into five categories (Table 3). Three categories contain translated gene products that are related to known proteins: 5,074 (25.9%) were defined as identical to a known human protein (Category I proteins); 4,104 (21.0%) were defined as similar to a known protein (Category II proteins); and 2,531 (12.9%) as domain-containing proteins (Category III proteins). In total, we were able to assign biological function to 59.9% of H-Inv proteins by similarity or motif searches. The remaining proteins, for which no biological functional was inferred, were annotated as conserved hypothetical proteins (Category IV proteins; 1,706, 8.7%) if they had a high level of similarity to other hypothetical proteins in other species, or as hypothetical proteins (Category V proteins; 6,159, 31.5%) if they did not.

To predict the functions of hypothetical proteins (Category IV and V proteins), we used 196 sequence patterns of functional importance derived from tertiary structures of protein modules, termed 3D keynotes (Go 1983; Noguti et al. 1993). Application of the 3D keynotes to the H-Inv proteins resulted in the prediction of functions in 350 hypothetical proteins (see Protocol S1).

#### Features of ORFs deduced from human FLcDNAs

The mean and median lengths of predicted ORFs were calculated for the 19,574 H-Inv proteins. These were 1,095 bp and 806 bp, respectively (Table 4). The values obtained were smaller than those from other eukaryotes, and are inconsistent with estimates reported previously (Shoemaker et al. 2001). However, as has been seen in the earlier annotation of the fission yeast genome (Das et al. 1997), our dataset might contain stretches which mimic short ORFs. This would lead to a bias in our ORF prediction and result in an erroneous estimate of the average ORF length. We examined the size distributions of ORFs from the five categories, and found that the distribution pattern was quite similar across categories. The exception was Category V, in which short ORFs were unusually abundant (Figure S3). Judging from the length distribution of ORFs in the five categories of H-Inv proteins, the majority of ORFs shorter than 600 bps in Category V seemed questionable. In order to have a protein dataset that contains as many sequences to be further analyzed as possible, we have taken the longest ORFs over 80 amino acids if no significant candidates were detected by the sequence similarity and gene prediction (see Figure S1). The consequence of this is that Category V appears to contain short questionable ORFs, a certain fraction of which may be prediction errors. Nevertheless, these ORFs could be true. It is also possible that those ORFs were in fact translated in vivo when we curated the cDNAs manually. The existence of many functional short proteins in the human proteome is already confirmed, and there are 199 known human proteins that are 80 amino acids or shorter in the current Swiss-Prot database. We think that the H-Inv hypothetical proteins require experimentally verification in the future. Excluding the hypothetical proteins from the analysis, we obtained mean and median lengths for the ORFs of 1,368 bp and 1,130 bp, respectively, which are reasonably close to those for other eukaryotes (Table 4).

**Table 4:**
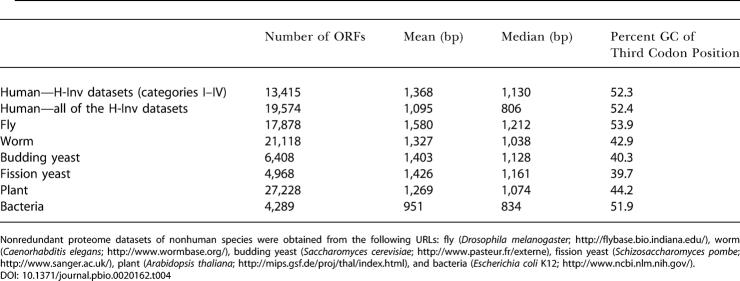
The Features of Predicted ORFs

Nonredundant proteome datasets of nonhuman species were obtained from the following URLs: fly (Drosophila melanogaster; http://flybase.bio.indiana.edu/), worm (Caenorhabditis elegans; http://www.wormbase.org/), budding yeast (Saccharomyces cerevisiae; http://www.pasteur.fr/externe), fission yeast (Schizosaccharomyces pombe; http://www.sanger.ac.uk/), plant (Arabidopsis thaliana; http://mips.gsf.de/proj/thal/index.html), and bacteria (Escherichia coli K12; http://www.ncbi.nlm.nih.gov/)

Of the 4,104 Category II proteins, 3,948 proteins (96.2%) were similar to the functionally identified proteins of mammals (Figure S4). This implies that the predicted functions in this study were based on the comparative study with closely related species, so that the functional assignment retains a high level of accuracy if we suppose that protein function is more highly conserved in more closely related species. Moreover, the patterns of codon usage and the codon adaptation index (CAI; http://biobase.dk/embossdocs/cai.html) of H-Inv proteins were investigated (Table S2). The results indicated that the ORF prediction scheme worked equally well in the five similarity categories of H-Inv proteins.

Each H-Inv protein in the five categories was investigated in relation to the tissue library of origin (Table S3). We found that at least 30% of the clones mainly isolated from dermal connective, muscle, heart, lung, kidney, or bladder tissues could be classified as Category I proteins. Hypothetical proteins (Category V), on the other hand, were abundant in both endocrine and exocrine tissues. This bias may indicate that expression in some tissues may not have been studied in enough detail. If this is the case, then there is likely a significant gap between our current knowledge of the human proteome and its true dimensions.

#### Non-protein-coding genes

Over recent years, ncRNAs have been found to play key roles in a variety of biological processes in addition to their well-known function in protein synthesis (Moore and Steitz 2002; Storz 2002). Analysis of the H-Inv cDNA dataset revealed that 6.5% of the transcripts are possibly non-protein-coding, although the number is much smaller than that estimated in mice (Okazaki et al. 2002). We believe that this difference between the two species is mainly due to the larger number of mouse libraries that were used and to a rare-transcript enrichment step that was applied to these collections.

**Figure 5:**
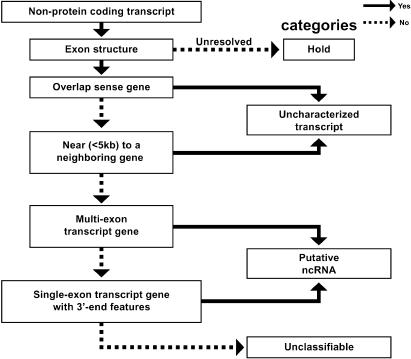
The Manual Annotation Flow Chart of ncRNAs Candidate non-protein-coding genes were compared with the human genome, ESTs, cDNA 3′-end features and the locus genomic environment. The candidates were then classified into four categories: hold (cDNAs improperly mapped onto the human genome); uncharacterized transcripts (transcripts overlapping a sense gene or located within 5 kb of a neighboring gene with EST support); putative ncRNAs (multiexon or single exon transcripts supported by ESTs or 3′-end features); and unclassifiable (possible genomic fragments).

To identify ncRNAs, we manually annotated 1,377 representative non-protein-coding transcripts, which were classified into four categories (see Table 3; Figure 5): putative ncRNAs, uncharacterized transcripts (possible 3′ UTR fragments supported by ESTs), unclassifiable transcripts (possible genomic fragments), and hold transcripts (not stringently mapped onto the human genome). Of these, 296 (19.5%) were putative ncRNAs with no neighboring transcripts in the close vicinity (> 5 kb) and supported by ESTs with a poly-A signal or a poly-A tail, indicating that these may represent genuine ncRNA genes. On the other hand, a large fraction of the non-protein-coding transcripts (675; 44.5%) were classified as possible 3′ UTRs of genes that were mapped less than 5 kb upstream. The 5-kb range is an arbitrary distance that we defined as one of our selection criteria for identifying ncRNAs. However, authentic non-protein-coding genes might be located adjacent to other protein-coding genes (as described earlier). Thus, some of the transcripts initially annotated as uncharacterized ESTs may correspond to ncRNAs when these sequences satisfy the other selection criteria.

We defined a manual annotation strategy (Figure 5) that allowed us to select convincing putative ncRNAs with various lines of supporting evidence. These are the following: absence of a neighboring gene in the close vicinity, overlap with human or mouse ESTs, occurrence in the 3′ end of cDNA sequences, as well as overlap with mouse cDNAs. Out of 296 annotated putative ncRNAs, we identified 47 ncRNAs with conserved RNA secondary structure motifs (Rivas and Eddy 2001), and nearly 60% of these were found expressed in up to eight human tissues (data not shown), indicating that the manual curation strategy employed in this study may facilitate the identification of novel non-protein-coding genes in other species.

#### The functions of human proteins identified through an analysis of domains

Proteins in many cases are composed of distinct domains each of which corresponds to a specific function. The identification and classification of functional domains are necessary to obtain an overview of the whole human proteome. In particular, the analysis of functional domains allows us to elucidate the evolution of the novel domain architectures of genes that life forms have acquired in conjunction with environmental changes. The human proteome deduced from the H-Inv cDNAs was subjected to InterProScan, which assigned functional motifs from the PROSITE, PRINTS, SMART, Pfam, and ProDom databases (Mulder et al. 2003). A total of 19,574 H-Inv proteins were examined, and 9,802 of them (50.1%) were assigned at least one InterPro code that was classified into either repeats (a region that is not expected to fold into a globular domain on its own), domains (an independent structural unit that can be found alone or in conjunction with other domains or repeats), and/or families (a group of evolutionarily related proteins that share one or more domains/repeats in common) when compared with those of fly, worm, budding and fission yeasts, *Arabidopsis thaliana,* and Escherichia coli (Table S4). Moreover, the proteins were classified according to the Gene Ontology (GO) codes that were assigned to InterPro entries (Table S5).

#### Identification of human enzymes and metabolic pathways

One of the most important goals of the functional annotation of human cDNAs is to predict and discover new, previously uncharacterized enzymes. In addition, revealing their positions in the metabolic pathways helps us understand the underlying biochemical and physiological roles of these enzymes in the cells. We thus searched for potential enzymes among the H-Inv proteins, and mapped them to a database of known metabolic pathways.

We could assign 656 kinds of potential Enzyme Commission (EC) numbers to 1,892 of the 19,574 H-Inv proteins based on matches to the InterPro entries and GO assignments and on the similarity to well-characterized Swiss-Prot proteins (see Dataset S2). The number of characterized human enzymes significantly increased through this analysis. The most abundant enzymes in the H-Inv proteins were protein–tyrosine kinases (EC 2.7.1.112), which is consistent with the large number of kinases found in the InterPro assignments. The other major enzymes were small monomeric GTPase (EC 3.6.1.47), adenosinetriphosphatase (EC 3.6.1.3), phosphoprotein phosphatase (EC 3.1.3.16), ubiquitin thiolesterase (EC 3.1.2.15), and ubiquitin-protein ligase (EC 6.3.2.19). These enzymes are members of large multigene families that are important for the functions of higher organisms. Furthermore, we could assign 726 EC numbers to mouse representative transcripts and proteins (Okazaki et al. 2002), and most of them appeared to be shared between human and mouse (data not shown). The high similarity of the enzyme repertoire between these two species is not surprising if we consider the close evolutionary relatedness between them. It does, however, indicate the usefulness of the mouse as a model organism for studies concerning metabolism.

We then mapped all H-Inv proteins on the metabolic pathways of the KEGG database, a large collection of information on enzyme reactions (Kanehisa et al. 2002). In total, we mapped 963 H-Inv proteins on a total of 1,613 KEGG pathways, of which 641 were based on their EC number assignments (Figure S5). Those based on EC number assignments do not necessarily function as they are assigned because they have yet to be verified experimentally. However, if all other enzymes along the same pathway exist in humans, the functional assignment has a high probability of being correct. Using this method, we discovered a total of 32 newly assigned human enzymes from the H-Inv proteins with the support of KEGG pathways (Table S6). For example, we identified (1) pyridoxamine-phosphate oxidase (EC 1.4.3.5; AK001397), an enzyme in the “salvage pathway,” the function of which is the reutilization of the coenzyme pyridoxal-5′-phosphate (its role in epileptogenesis was recently reported [Bahn et al. 2002]), (2) ATP-hydrolysing 5-oxoprolinase (EC 3.5.2.9; AL096750) that cleaves 5-oxo-L-proline to form L-glutamate (whose deficiency is described in the Online Mendelian Inheritance in Man [OMIM] database [ID=260005]), and (3) N-acetylglucosamine-6-phosphate deacetylase (EC 3.5.1.25; BC018734), which catalyzes N-acetylglucosamine at the second step of its catabolism, the activity of which in human erythrocytes was detected by a biochemical study (Weidanz et al. 1996). Many of the newly identified enzymes were supported by currently available experimental and genomic data. An example is a putative urocanase (EC 4.2.1.49; AK055862) that mapped onto the “histidine metabolism” that urocanic acid catabolises. A ^14^C Histidine tracer study unexpectedly revealed that NEUT2 mice deficient in 10-formyltetrahydrofolate dehydrogenase (FTHFD) excrete urocanic acid in the urine and lack urocanase activity in their hepatic cytosol (Cook 2001). We then found that both the FTHFD and AK055862 genes were located within the same NCBI human contig (NT005588) on Chromosome 3. Moreover, the distance between the two genes was consistent with the genetic deletion of NEUT2 (> 30 kb). We thus assumed that FTHFD and urocanase might be coincidentally defective in mice. This analysis could confirm that the AK055862 protein is a true urocanase. This example demonstrates that this kind of in silico analysis is a powerful method in defining the functions of proteins.

### Polymorphism in the Transcriptome

#### Sites of potential polymorphism in cDNAs

Due to the rapidly increasing accumulation of genetic polymorphism data, it is necessary to classify the polymorphism data with respect to gene structure in order to elucidate potential biological effects (Gaudieri et al. 2000; Sachidanandam et al. 2001; Akey et al. 2002; Bamshad and Wooding 2003). For this purpose, we examined the relationship between publicly available polymorphism data and the structure of our H-Inv cDNA sequences. A total of 4 million single nucleotide polymorphisms (SNPs) and insertion/deletion length variations (indels) with mapping information from the Single Nucleotide Polymorphism Database (dbSNP; http://www.ncbi.nlm.nih.gov/SNP/, build 117) (Sherry et al. 1999) were used for the search. We could identify 72,027 uniquely mapped SNPs and indels in the representative H-Inv cDNAs and observed an average SNP density of 1/689 bp. To classify SNPs and indels with respect to gene structure, the genomic coordinates of SNPs were converted into the corresponding nucleotide positions within the mapped cDNAs. The SNPs and indels were classified into three categories according to their positions: 5′ UTR, ORF, and 3′ UTR (Table 5). The density of indels was higher in 5′ UTRs (1/15,999 bp) and 3′ UTRs (1/12,553 bp) than in ORFs (1/45,490 bp). This is possibly due to different levels of functional constraints. We also examined the length of indels and found a higher frequency of indels in those ORFs that had a length divisible by three and that did not change their reading frames. We observed that the density of SNPs was higher in both the 5′ and 3′ UTRs (1/569 bp and 1/536 bp, respectively) than in ORFs (1/833 bp).

**Table 5:**
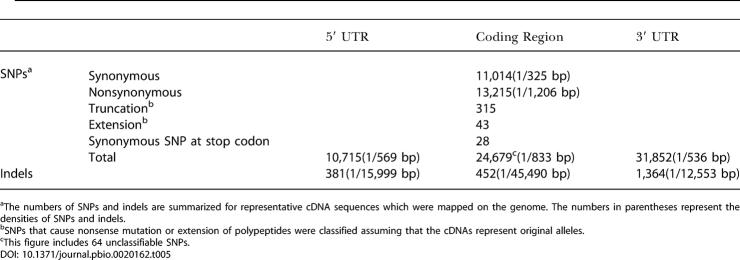
The Numbers of SNPs and indels Occurring in the Representative cDNAs

^a^The numbers of SNPs and indels are summarized for representative cDNA sequences which were mapped on the genome. The numbers in parentheses represent the densities of SNPs and indels

^b^SNPs that cause nonsense mutation or extension of polypeptides were classified assuming that the cDNAs represent original alleles

^c^This figure includes 64 unclassifiable SNPs

SNPs located in ORFs were classified as either synonymous, nonsynonymous, or nonsense substitutions (Table 5). We identified 13,215 nonsynonymous SNPs that affect the amino acid sequence of a gene product. At least 4,998 of these nonsynonymous SNPs are “validated” SNPs (as defined by dbSNP). This data can be used to predict SNPs that affect gene function. SNPs that create stop codons can cause polymorphisms that may critically alter gene function. We identified 358 SNPs that caused either a nonsense mutation or an extension of the polypeptide. We classified these 358 SNPs into these two types based on the alleles of the cDNA. Most of these SNPs (315/358) were predicted to cause truncation of the gene products and produce a shorter polypeptide compared with the alleles of H-Inv cDNAs. For example, Reissner's fiber glycoprotein I (AK093431) contains a nonsense SNP that results in the loss of the last 277 amino acids of the protein, and consequently the loss of a thrombospondin type I domain located in its C-terminal end. This SNP is highly polymorphic in the Japanese population, the frequencies of *G* (normal) and *T* (termination) being 0.43 and 0.57, respectively. As seen in this example, the identification of SNPs within cDNAs provides important insights into the potential diversity of the human transcriptome. Thus, polymorphism data crossreferenced to a comprehensively annotated human transcriptome might prove to be a valuable tool in the hands of researchers investigating genetic diseases.

#### Sites of microsatellite repeats

Among the 19,442 representative protein-coding cDNAs, we identified a total of 2,934 di-, tri-, tetra-, and penta-nucleotide microsatellite repeat motifs (Table 6). Interestingly, 1,090 (37.2%) of these were found in coding regions, the majority of which (86.9%) were tri-nucleotide repeats. Di-, tetra-, and penta-nucleotide repeats made up the greatest proportion of repeats in 5′ UTRs and 3′ UTRs. Coding regions contained mostly tri-nucleotide repeats. This result is consistent with the idea that microsatellites are prone to mutations that cause changes in numbers of repeats. Only tri-nucleotide repeats can conserve original reading frames when extended or shortened by mutations. A previous study showed that many of the microsatellite motifs identified in human genomic sequences, including those in coding regions, are highly polymorphic in human populations (Matsuzaka et al. 2001). We found this to be the case in our study: 36 of the microsatellite repeats we detected were found to be polymorphic in human populations according to dbSNP records (data not shown). We identified 216 microsatellite repeats in 213 genes that showed contradictory numbers of repeats between cDNA and genome sequences (see Dataset S3). This figure includes 25 microsatellites in ORFs that have the potential to alter the protein sequences. Individual cases need to be verified by further experimental studies, but many of these microsatellites may really be polymorphic in human populations and have marked phenotypic effects.

**Table 6:**
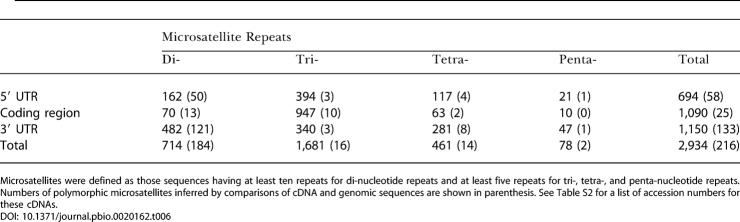
The Numbers of Microsatellite Repeat Motifs That Occurred in the Representative cDNAs

Microsatellites were defined as those sequences having at least ten repeats for di-nucleotide repeats and at least five repeats for tri-, tetra-, and penta-nucleotide repeats. Numbers of polymorphic microsatellites inferred by comparisons of cDNA and genomic sequences are shown in parenthesis. See Table S2 for a list of accession numbers for these cDNAs

There were 382 cDNAs that possessed two or more microsatellites in their nucleotide sequences. This is illustrated in RBMS1 (BC018951), a cDNA which encodes an RNA-binding motif. This cDNA has four microsatellites, (GGA)_7_, (GAG)_9_, (GAG)_6_, and (GCC)_6_, in its 5′ UTR. These microsatellites are all located at least 98 bp upstream of the start codon, but they could still have pronounced regulatory effects on gene expression. Another example is the cDNA that encodes CAGH3 (AB058719). This cDNA has four microsatellites, (CAG)_8_, (CAG)_6_, (CAG)_8_, and (CAG)_8,_ all of which are located within the ORF. These microsatellites all encode stretches of poly-glutamine, which are known to have transcription factor activity (Gerber et al. 1994) and often cause neurodegenerative diseases when the number of repeats exceeds a certain limit. A typical example of a disorder caused by these repeats is Huntington's disease (Andrew et al. 1993; Duyao et al. 1993; Snell et al. 1993).

We also searched for repeat motifs containing the same amino acid residue in the encoded protein sequences. We located a total of 3,869 separate positions where the same amino acid was repeated at least five times. The most frequent repetitive amino acids are glutamic acid, proline, serine, alanine, leucine, and glycine. The glutamine repeats of this nature were found in 160 different locations.

### Evolution of the Human Transcriptome

Beyond the study of individual genes, the comparison of numerous complete genome sequences facilitates the elucidation of evolutionary processes of whole gene sets. Moreover, the FLcDNA datasets of humans and mice give us an opportunity to investigate the genome-wide evolution of these two mammals by using the sequences supported by physical clones. Here we compared our human cDNA sequences with all proteins available in the public databases. Focusing on our results, we discuss when and how the human proteome may have been established during evolution. Furthermore, the evolution of UTRs is examined through comparisons with cDNAs from both primates and rodents.

#### Conserved and derived protein-coding genes in humans

**Figure 6:**
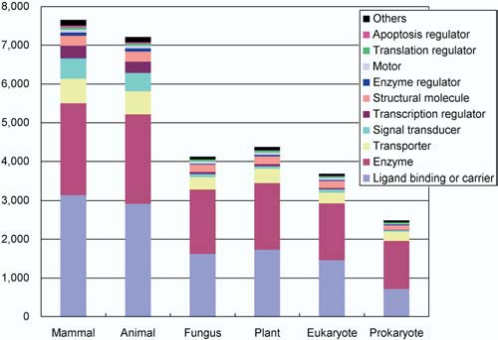
The Functional Classification of H-Inv Proteins That Are Homologous to Proteins in Each Taxonomic Group The numbers of representative H-Inv cDNAs with sequence homology to other species' proteins (*E* < 10^−5^) were calculated. The cDNAs for which we could not assign any functions were discarded. Mammalian species were excluded from the “animal” group. “Eukaryote” represents eukaryotic species other than those included in the mammal, animal, fungi, and plant groups. See also Table S7.

An advantage of large-scale cDNA sequencing is that it can generate a nearly complete gene set with good evidence for transcription. The human proteome deduced from the FLcDNA sequences gives us an opportunity to decipher the evolution of the entire proteome. Here we compare the representative H-Inv cDNAs with the Swiss-Prot and TrEMBL protein databases using FASTY (Pearson 2000), and we describe the distributions of the homologs among taxonomic groups at two different similarity levels. The number of representative H-Inv cDNAs that have homolog(s) in a given taxon was counted (Figure S6), and the cDNAs were classified into functional categories (Figure 6). These results indicated that homologs of the human proteins were probably conserved much more in the animal kingdom than in the others at both moderate (*E* <10^−10^) and weak (*E* < 10^−5^) similarity levels (see Figure S6). Moreover, human sequences had as many nonmammalian animal homologs as mammalian homologs, with seemingly little bias to any one function (see Figure 6). This suggests that the genetic background of humans may have already been established in an early stage of animal evolution and that many parts of the whole genetic system have probably been stable throughout animal evolution despite the seemingly drastic morphological differences between various animal species. This result is consistent with our previous observation that the distribution of the functional domains is highly conserved among animal species (see Table S4). The number of homologs may have been inflated by recent gene duplication events within the human lineage. Hence we counted the number of paralog clusters instead of cDNAs that had homologs in the databases, and obtained essentially the same results (Figure S7).

This analysis also revealed a number of potential human-specific proteins, which did not have any homologs in the current sequence databases. In this case the creation of lineage-specific genes through speciation is not completely excluded. However, most ORFs with no similarity to known proteins would not be genuine for the reasons discussed above. Therefore, the number of “true” human-specific proteins is expected to be relatively small.

We conducted further BLASTP searches matching entries from the Swiss-Prot database against the H-Inv dataset itself. As a result, 12,813 (45.3%) of 28,263 vertebrate proteins had homologs in nonvertebrates at *E* < 10^–30^. Taking into account that the dataset is relatively small (approximately 12,000 sequences) and as a result may be biased, animal species may conceivably share a similar protein-coding gene set.

Ohno (1996) proposed that the emergence of a large number of animal phyla in a short period of time would endow them with almost identical genomes. These were collectively referred to as the pananimalia genome. Our data support Ohno's hypothesis from the perspective that the basic gene repertoires of animals are essentially highly similar among diverse species that have evolved separately since the Cambrian explosion. Subsequently, morphological evolution seems to have been brought about mainly by changes in gene regulation. The number of transcription regulator homologs is different between animals and other phyla (Table S7). In this analysis it was not possible to examine the genes recently deleted from the human lineage. However, the similarity of the proteome sets between distantly related mammals such as human and mouse (Waterston et al. 2002) suggests that not many genes have been deleted specifically from humans since humans and mice diverged.

A unique feature of the Animalia proteome is, for example, the presence of apoptosis regulator homologs, which are found widely in the animal kingdom, whilst they are rare in the other phyla (Table S7). Since apoptosis plays an important role during the development of multicellular animals, this observation indicates that apoptosis was established independently of both plants and fungi during the early evolution of multicellularization in the kingdom Animalia. Likewise, signal transducers and cell-adhesion proteins are distinctive. In contrast, enzymes, translation regulators, molecular chaperones, etc. were highly conserved among all taxonomic groups. These proteins may have played such essential roles that any alterations were eliminated by strong purifying selection. It is assumed some functions were presumably derived from ancient endocellular symbionts (mitochondria and chloroplasts) (Martin 2002).

#### Evolution of untranslated regions

**Figure 7:**
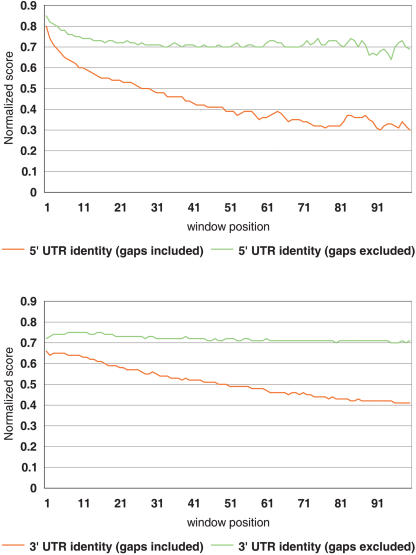
Window Analysis of Similarity between Human and Mouse UTRs Results for 5′ UTRs presented above and for 3′ UTRs below. The whole mRNA sequences were aligned using a semiglobal algorithm as implemented in the map program (Huang 1994) with the following parameters: match 10, mismatch −3, gap opening penalty −50, gap extension penalty −5, and longest penalized gap 10; the terminal gaps are not penalized at all. A window size of 20 bp was used with a step of 10 bp. The analysis window was moved upstream and downstream of start and stop codons, respectively. The normalized score for a given window is calculated as a fraction of an average score for all UTRs in a given window over the maximum score observed in all 5′ or 3′ UTRs, respectively.

The UTRs of mRNA are known to be involved in the regulation of gene expression at the posttranscriptional level through control of translation efficiency (Kozak 1989; Geballe and Morris 1994; Sonenberg 1994), mRNA stability (Zaidi and Malter 1994; McCarthy and Kollmus 1995), and mRNA localization (Curtis et al. 1995; Lithgow et al. 1997). Only a few studies on very limited datasets have been carried out so far to describe quantitatively either the evolutionary dynamics of mRNA UTRs (Larizza et al. 2002), or their general structural and compositional features (Pesole et al. 1997). The human transcriptome presented here along with the murid data obtained mainly from the FANTOM2 project enables us to stabilize a mammalian genome perspective on the subject (Table S8). A sliding window analysis of UTR sequence identities between humans and mice revealed a positive correlation between the number of indels in an untranslated region and the distance from the coding sequence (Figure 7). Unlike indels, mismatches are distributed equally along whole untranslated regions. In other words, indels seem to be less tolerated in close proximity to a coding sequence, while substitutions are evenly distributed along the untranslated regions of the mRNAs. This seems to be a general pattern observed similarly in other species (data not shown). Indels in UTRs may have been avoided so that the distance between the coding region and a signal sequence for regulation in the UTR could be conserved throughout evolution, while purifying selection against substitutions appeared to be relatively weak.

#### Untranslated region replacement

A replacement of the entire UTR may lead to drastic changes in gene expression, especially if a UTR having a posttranscriptional signal is replaced by another. We compared the evolutionary distances of UTRs between primate and rodent orthologous sequences. We based our analysis on the UTR sequence distances that contradicted the expected phylogenetic tree of relatedness. We could detect 149 UTR replacements distributed among different species. Some of the observed replacements may result from selection of different AS isoforms of a single locus in different species. This is particularly likely if an AS event involves an alternative first or last exon. It seems that UTR replacements are more frequent in rodents than in primates, but the difference is not statistically significant at the 5% significance level (Table S9). We detected a UTR replacement in less than 2% of the analyzed sequences. The evolutionary consequences could be significant because the UTR replacement might result in changes in expression level or the loss of an mRNA localization signal.

### The H-Invitational Database

All the results of the mapping of the FLcDNA sequences onto the human genome, the clustering of FLcDNA sequences, sequence alignments, detection of AS transcripts, sequence similarity searches, functional annotation, protein structure prediction, subcellular localization prediction, SNP mapping, and evolutionary analysis, as well as the basic features of FLcDNA sequences, are stored in the H-InvDB (Figure S8). The H-InvDB is a unique database that integrates annotation of sequences, structure, function, expression, and diversity of human genes into a single entity. It is useful as a platform for conducting in silico data mining. The database has functions such as a keyword search, a sequence similarity search, a cDNA search, and a searchable genome browser. It is hoped that the H-InvDB will become a vital resource in the support of both basic and applied studies in the fields of biology and medicine.

We constructed two kinds of specialized subdatabases within the H-InvDB. The first is the Human Anatomic Gene Expression Library (H-Angel), a database of expression patterns that we constructed to obtain a broad outline of the expression patterns of human genes. We collected gene expression data from normal and diseased adult human tissues. The results were generated using three methods on seven different platforms. These included iAFLP (Kawamoto et al. 1999; Sese et al. 2001), DNA arrays (long oligomers, short oligomers [Haverty et al. 2002], cDNA nylon microarrays [Pietu et al. 1999], and cDNA glass slide microarrays [Arrays/IMAGE-Genexpress]), and cDNA sequence tags (SAGE [Velculescu et al. 1995; Boon et al. 2002], EST data [Boguski et al. 1993; Kawamoto et al. 2000], and MPSS [Brenner et al. 2000]). By normalizing levels of gene expression in experiments conducted with different methods, we determined the gene expression patterns of 19,276 H-Inv loci in ten major categories of tissues. This analysis allowed us to clearly distinguish broadly and evenly expressed housekeeping genes from those expressed in a more restricted set of tissues (details will be published elsewhere). The H-Angel database comprises the largest and most comprehensive collection of gene expression patterns currently available. Also provided is a classification of human genes by expression pattern.

The second subdatabase of the H-InvDB is DiseaseInfo Viewer. This is a database of known and orphan genetic diseases. We tried to relate H-Inv loci with disease information in two ways. Firstly, 613 H-Inv loci that correspond with known, characterized disease-related genes were identified by creating links to entries in both LocusLink (http://www.ncbi.nlm.nih.gov/LocusLink/) and OMIM (Hamosh et al. 2002). To explore the possibility that cDNAs encoding unknown proteins may be related to “orphan pathologies” (diseases that have been mapped to chromosomal regions, but for which associated genes have not yet been described), we generated a list of H-Inv loci that co-localized with these cytogenetic regions. The nonredundant orphan disease dataset we created consists of 586 diseases identified through OMIM (http://www.ncbi.nlm.nih.gov/Omim/, ver. Jan. 2003), with an additional 108 identified from GenAtlas (http://www.dsi.univ-paris5.fr/genatlas/, ver. Jan. 2003). Using the OMIM and GenAtlas databases in conjunction with the annotation results from the H-InvDB may accelerate the process of identifying candidate genes for human genetic diseases.

### Concluding Remarks

There are a number of established collections of nonhuman cDNAs, such as those of Drosophila melanogaster (Stapleton et al. 2002), Danio rerio (Clark et al. 2001), Arabidopsis thaliana (Seki et al. 2002), Plasmodium falciparum (Watanabe et al. 2002), and Trypansoma cruzi (Urmenyi et al. 1999). The most extensive collection of mammalian cDNAs so far has been that of the RIKEN/FANTOM mouse cDNA project (Kawai et al. 2001; Okazaki et al. 2002). This wealth of information has spurred a wide variety of research in the areas of both gene expression profiling (Miki et al. 2001) and protein–protein interactions (Suzuki et al. 2001). The H-InvDB provides an integrative means of performing many more such analyses based on human cDNAs.

The most important findings that have resulted from the cDNA annotation are summarized here.

(1) The 41,118 H-Inv cDNAs were found to cluster into 21,037 human gene candidates. Comparison with known and previously predicted human gene sets revealed that these 21,037 hypothesized gene clusters contain 5,155 new gene candidates.

(2) The primary structure of 21,037 human gene candidates was precisely described. For the majority of them we observed that both first introns and last exons tended to be longer than the other introns and exons, respectively, implying the possible existence of intriguing mechanisms of transcriptional control in first introns.

(3) We discovered the existence of 847 human gene candidates that could not be convincingly mapped to the human genome. This result suggested that up to 3.7%–4.0% of the human genome sequences (NCBI build 34 assembly) may be incomplete, containing either unsequenced regions or regions where sequence assembly has been performed in error.

(4) Based on H-Inv cDNAs, we were able to define an experimentally validated AS dataset. The dataset was composed of 3,181 loci that encoded a total of 8,553 AS isoforms. In the 55% of ORFs containing AS isoforms, the pattern of alternative exon usage was found to encode different functional domains at the same loci.

(5) A standardized method of human curation for the H-Inv cDNAs was created under the tacit consensus of international collaborations. Using this method, we classified 19,574 H-Inv proteins into five categories based on sequence similarity and structural information. We were able to assign functional definitions to 9,139 proteins, to locate function- or family-defining InterPro domains in 2,503 further proteins, and to identify 7,800 transcripts as good candidates for hypothetical proteins.

(6) A total of 1,892 H-Inv proteins were assigned identities as one of 656 different EC-numbered enzymes. This enzyme library includes 32 newly identified human enzymes on known metabolic pathway maps and comprises the largest collection of computationally validated human enzymes.

(7) Based on a variety of supporting evidence, 6.5% of H-Inv loci (1,377 loci) do not have a good protein-coding ORF, of which 296 loci are strong candidates for ncRNA genes.

(8) We identified and mapped 72,027 SNPs and indels to unique positions on 16,861 loci. Of these, 13,215 nonsynonymous SNPs, 358 nonsense SNPs, and 452 indels were found in coding regions and may alter protein sequences, cause phenotypic effects, or be associated with disease. In addition, we identified 216 polymorphic microsatellite repeats on 213 loci, 25 of which were located in coding regions.

(9) During human proteome analysis, it was suggested that the basic gene set of humans might have been established in the early stage of animal evolution. Our analysis of UTRs revealed that insertions or deletions near coding regions were rare when compared with substitutions, though in some cases drastic changes such as UTR replacements occurred.

(10) A consequence of the annotation process and our related research was the development of the H-InvDB to contain our annotation work. H-InvDB is a comprehensive database of human FLcDNA annotations that stores all information produced in this project. As a subdivision of H-InvDB, we developed two other specialized subdatabases: H-Angel and DiseaseInfo Viewer. H-Angel is a database of gene expression patterns for 19,276 loci. DiseaseInfo Viewer is a database of known disease-related genes and loci co-localized with 694 orphan pathologies. These pathologies were mapped onto the genome but were not identified experimentally.

In the H-Inv project, we collected as many FLcDNAs as possible and conducted extensive analyses concerning the quality of cDNAs, such as detection of frameshift errors, retained introns, and internal poly-A priming, under a unified criterion. Although these analyses are still in an elementary state, we store these results in H-InvDB to share this information with the biological community. We believe that this is an important contribution of our project, because it will provide a reliable way to control the quality of the cDNA clones. In the future, this information will be useful for improving the methods of clone library construction.

It has been suggested that the human genome encodes 30,000 to 40,000 genes. In this study we comprehensively evaluated more than 21,000 human gene candidates (up to 70% of the total). Thus, efforts should be continued by the H-Inv consortium and others to “fully” characterize the human transcriptome. For this purpose new technologies should be implemented that are more sensitive in detecting rarely expressed genes and AS transcripts. Nevertheless, there are unavoidable limitations for human cDNA collections, such the identification of embryo-specific genes, for which other approaches should be employed. One alternative is the use of ab initio predictions from genomic sequences, in conjunction with expression profiling studies, to identify rarely expressed genes that share structural similarity to known genes. Additionally, a better characterization of *cis*-regulatory element units may help to define the boundary of other genes that are undetected by current gene prediction programs. Another area that remains to be explored is the identification of potential hidden RNA gene families that may play vital roles, such as the recently uncovered family of microRNA genes, which is involved in the regulation of expression of other genes (for review see Ambros 2001; Moss 2002).

The proteome determination aspects of this project, including the identification of new enzymes and hypothetical proteins, should stimulate more focused biochemical studies. The functional classifications may allow definition of subproteomes that are related to different physiological processes. The H-Inv transcriptome based on the definition of a consensus proteome (the H-Inv proteins) links both the analysis of genomic DNA and direct proteome analysis with the study of expressed mRNA analysis from different tissues, cells, and disease states. It creates a standard for the comparison of disease-related alterations of the human proteome. Moreover, comparison with pathogen proteomes may yield many possible drug target proteins. Also, the annotation of ncRNAs raises the possibility of novel “smart” therapeutics that could either inhibit or mimic the mechanisms of these RNAs.

The H-Inv project is the first ever comprehensive compilation of curated and annotated human FLcDNAs. The project may lead to a more complete understanding of the human transcriptome and, as a result, of the human proteome. The preceding examples of the importance of the H-Inv data in understanding human physiology and evolution represent just a small fraction of the research potential of the H-InvDB.

In conclusion, the H-InvDB platform constructed to hold the results of the comprehensive annotations performed by our international team of collaborators represents a substantial contribution to resources that are needed for further exploration of both human biology and pathology.

## Materials and Methods

### 

#### cDNA resources

41,118 H-Inv cDNAs were sequenced by the Human Full-Length cDNA Sequencing Project (Ota et al. 1997; Yudate et al. 2001; Ota et al. 2004) at the Helix Research Institute, the Institute of Medical Science at the University of Tokyo, and the Kazusa DNA Research Institute (20,999 sequences in total); the Kazusa cDNA Sequencing Project (Kikuno et al. 2002) at the Kazusa DNA Research Institute (2,000 sequences); the Mammalian Gene Collection (Strausberg et al. 1999) at the National Institutes of Health in the United States (11,806 sequences); the German Human cDNA Project (Wiemann et al. 2001) coordinated by the Deutsches Krebsforschungszentrum in Heidelberg (5,555 sequences); and the Chinese National Human Genome Center at Shanghai (Hu et al. 2000) (758 sequences).

#### Mapping human cDNAs to the human genome and the comparison of the mapped H-Inv cDNAs with other annotated datasets

We have mapped human cDNA sequences to the human genome sequence corresponding to the NCBI build 34 assembly. The datasets we used were a set of 41,118 H-Inv cDNAs and a set of 37,488 human RefSeq sequences available on 15 July 2002 and on the 1 September 2003, respectively. All the revisions for H-Inv cDNA sequences until August 2003 were applied in the datasets. Before performing the mapping procedure, all the repetitive and low-complexity sequences in all the cDNA sequences were masked using RepeatMasker (http://ftp.genome.washington.edu/RM/RepeatMasker.html) and Repbase 7.5. Then we used the cross_match program to mask the remaining vector sequences in each cDNA sequence. Any poly-A tails were also masked by using a custom-made Perl script. In the first step of the mapping procedure, we conducted BLASTN (ver.2.2.6) searches of all the sequences against the human genome sequence and extracted the corresponding genomic regions for each query sequence. Then we used est2genome (EMBOSS package ver.2.7.1) to align each sequence to the genomic region with a threshold of 95% identity and 90% coverage. Coverage of each cDNA sequence was calculated excluding those from the vector and poly-A tails that were masked in the previous step. If the sequences were mapped to multiple positions on the human genome, then we selected their best locus based on the identity, length coverage, and number of exons of those sequences. As a result, 77,315 sequences (including 40,140 cDNAs from the H-Inv project) were successfully mapped onto the human genome and were clustered into 38,587 clusters based on sharing at least 1 bp of an exon on the same chromosome strand. We used all the mapped sequences, including human RefSeq sequences, to compare the clusters that included H-Inv cDNAs with those that consisted of only human RefSeq sequences. 20,190 clusters out of 38,587 consisted of only H-Inv cDNAs or both H-Inv cDNAs and human RefSeq sequences. The rest of the clusters consisted of RefSeq sequences only. All of the mapped cDNAs and the overlap with the RefSeq sequences can be viewed using G-integra in the H-InvDB (http://www.jbirc.aist.go.jp/hinv/g-integra/html/). The mapping procedure for all the unmapped cDNAs against the mouse genome was also performed, using a threshold of 60% identity and 90% coverage.

#### Clustering of unmapped sequences

The sequences that were not mapped onto the human genome were clustered by a single linkage clustering method. The similarity search was performed among all the unmapped sequences. The program used was MegaBLAST version 2.2.6 (Zhang et al. 2000). As with to the mapping strategy, some distinctive sequences (repetitive regions, contaminations from cloning vectors and poly-A tails) were excluded from the queries of the similarity search. The similarity was evaluated using the expected value (*E-*value) between two sequences. Only when the *E-*value of the two sequences was calculated to be 0, did we assume that a significant level of similarity was detected between the two sequences.

#### Identification of gene structure

In order to identify gene structure, we used only the representative H-Inv cDNAs. When detecting repetitive elements in cDNAs, RepeatMasker was conducted in a similar manner to the previous phase. We used curated cDNAs in which frameshift errors and remaining introns were removed.

#### Prediction of ORFs

We predicted ORFs in all 41,118 H-Inv cDNAs, as illustrated in Figure S1, based on the alignment of similarity searches by FASTY (Pearson 2000; Mackey et al. 2002) (ver. 3.4t11) and BLASTX (Altschul et al. 1990) (ver. 2.0.11), and gene prediction by GeneMark (McIninch et al. 1996) (http://opal.biology.gatech.edu/GeneMark/) (Table S10). Prior to the prediction of ORFs, we judged if the sequence had any frameshift errors or remaining introns (see Figure S1). During ORF prediction, we corrected the aforementioned sequence irregularities computationally.

#### Procedure of computational and human annotation

Prior to the human curation, we performed two computational automated annotation processes to select the representative clone for each locus and to predict function of H-Inv proteins (see Figure S2). We then assigned the most suitable data source ID to each H-Inv protein following a scheme illustrated in Figure S2 and referring to the information using newly developed annotation viewers, named SOUP location viewer, SOUP annotation viewer, and Similarity Motif ORF (SMO) Viewer (Figure S9). Questionable transcripts were determined by human curation based upon evidence such as the following: sequences with no similarity to a known protein or domain, sequences with a very short ORF, cDNAs with only a single exon, and sequences with no EST support. Only 959 (4.9%) of the computationally selected 19,574 representative H-Inv proteins had to be manually corrected. Another 3,142 (16.1%) of the H-Inv proteins had their functional assignment altered by manual curation.

#### Assignment of functional motifs

Nonredundant proteome datasets were obtained for fly (http://flybase.bio.indiana.edu/), worm (http://www.wormbase.org/), budding yeast (http://www.pasteur.fr/externe), fission yeast (http://www.sanger.ac.uk/), plant (http://mips.gsf.de/proj/thal/index.html), and a bacteria (ftp://ftp.ncbi.nih.gov/genbank/genomes/Bacteria/Escherichia_coli_K12/). The H-Inv proteins and other nonredundant proteome datasets were assigned InterPro codes by InterProScan ver. 3.1 (Mulder et al. 2003). The codes corresponded to families, domains, and repeats. GO terms were also assigned (see Table S5).

#### Evolutionary relationship of proteomes

The top 40 InterPro entries for the human proteome were compared with their equivalents from the fly, worm, yeasts, plant, and bacteria proteomes (see Table S4).

#### Protein domains and low-complexity inserted sequences

Folds were assigned by reverse PSI-BLAST (Altschul et al. 1997) searches of the amino acid sequences derived from the H-Inv cDNA against the SCOP database (Lo Conte et al. 2000). Information on protein and gene structures, with the exception of mouse and puffer fish, was obtained from the individual genome projects (Blattner et al. 1997; Kunst et al. 1997; CESC 1998; Adams et al. 2000; AGI 2000; Wood et al. 2002). The data for mouse and puffer fish were obtained from the Ensembl database (Hubbard et al. 2002).

#### Subcellular localization

Subcellular localization targeting signals and transmembrane helices of 40,352 H-Inv proteins were predicted using the PSORT II (Nakai and Horton 1999), TargetP (Emanuelsson et al. 2000), TMHMM, and SOSUI (Hirokawa et al. 1998) computer programs.

#### UTR sequences

We obtained the UTR sequences from three primates (*Pan troglodytes,* chimpanzee; *Macaca fascicularis,* crab-eating macaque; and *Macaca mulatta,* rhesus monkey) and two rodents (*Mus musculus,* house mouse; and *Rattus norvegicus,* Norwegian rat) that corresponded to UTRs from Homo sapiens. In order to do this, we mapped the cDNAs to the human or mouse genome. The corresponding rodent cDNAs were determined by using a human–mouse genome alignment provided by Ensembl. cDNAs of the primates and rodents were retrieved from the DDBJ/EMBL/GenBank databases using the cut off date of 15 July 2002. Additionally, we used the FANTOM2 mouse sequences released on 5 December 2002, and 4,063 5′ ESTs of chimpanzees (Sakate et al. 2003). Corresponding UTRs between human and other species were identified by aligning 5′ and 3′ ends of the human ORFs. To compare evolutionary distances, we analyzed 3,061 and 5,277 orthologous groups that consisted of at least three species' information for the 5′ and 3′ UTR sequences, respectively.

## Supporting Information

Dataset S1 List of Library Origins of H-Inv cDNAs (182 Libraries) The dataset consists of 41,118 H-Inv cDNAs that were cloned from cDNA libraries derived from 182 varieties of cell and tissue.
(33 KB XLS).

Dataset S2 List of H-Inv Proteins with Potential EC Numbers (1,892 H-Inv Proteins) The allotted EC numbers are based on the corresponding DNA databank records, UniProt/Swiss-Prot and TrEMBL records that show sequence similarity to the proteins, and InterPro records that the proteins hit.
(247 KB XLS).

Dataset S3 List of Polymorphic Microsatellites Inferred by Comparisons between the H-Inv cDNAs and Genomic Sequences (56 KB XLS).

Figure S1 Prediction of ORFs (A) Schematic diagram for the prediction of ORFs. This diagram illustrates the ORF prediction method used on all H-Inv cDNAs. The method was based upon the alignment of similarity searches using FASTY and BLASTX. Gene prediction was carried out using GeneMark. Prior to the prediction of ORFs, we judged if a sequence had any frameshift errors or remaining introns. During ORF prediction, we corrected those sequence irregularities computationally. Details of how sequence irregularities were predicted are described in (B) and (C).
(B) Schematic diagram for prediction of unspliced introns. This schematic diagram illustrates the prediction method used for unspliced introns.
(C) Schematic diagram for prediction of frameshift errors. Frameshift errors were inferred from cDNA–genome pairwise alignment gaps due to insertion or deletion, exception of multiple of 3 bp, or over 10 bp in either the query cDNA or genome.
(D) The statistics for the predicted frameshifts and unspliced introns.
(49 KB PDF).

Figure S2 Scheme of Prediction for Functional Annotation (A) Schematic diagram for determining a representative transcript for each locus. The procedure of computational autoannotation is illustrated. Prior to the human curation of the representative transcript of each H-Inv cluster, we performed computational autoannotation.
(B) Schematic diagram for functional prediction of H-Inv proteins. This schematic diagram illustrates the H-Inv autofunctional annotation pipeline that can determine the most appropriate data source ID, avoiding the following keywords that suggest proteins without experimental verification in the description; (1) hypothetical, (2) similar to, (3) names of cDNA clones (Rik, KIAA, FLJ, DKFZ, HSPC, MGC, CHGC, and IMAGE) and (4) names of InterPro domain frequent hitters.
(34 KB PDF).

Figure S3 Size Distribution of Predicted ORFs The size distribution of all H-Inv proteins among the five similarity categories.
(24 KB PDF).

Figure S4 Features of Category II Proteins A total of 4,104 H-Inv proteins were classified as Category II based on sequence similarity to functionally validated proteins. The table and figure show source species of proteins in public databases to which the Category II proteins were similar.
(9 KB PDF).

Figure S5 H-Inv KEGG Analysis Results (Images of KEGG Pathways) The images illustrate the metabolic pathways of KEGG database based on the EC number assignments to H-Inv proteins.
(47 KB PDF).

Figure S6 Numbers of Representative H-Inv cDNAs That Are Homologous to Proteins in Each Taxonomic Group Two thresholds (E < 10^−5^, white bars, and E < 10^−10^, black bars) were employed. The “animal” group does not include mammalian species. The “eukaryote” group represents eukaryotic species other than animals, fungi, and plants.
(9 KB PDF).

Figure S7 A Functional Classification of H-Inv Protein Families That Have Homologs in Each Taxonomic Group H-Inv protein families were identified by clustering H-Inv proteins using the single-linkage clustering method. Then, the number of homologs for each H-Inv protein family was calculated. Mammalian species are excluded from the “animal” group. “eukaryote” represents eukaryotic species other than animals, fungi, and plants.
**Single-linkage clustering**. All of the H-Inv proteins were compared with themselves by BLASTP and clustered with the thresholds of E-values of 10^−30^ and 10^−50^. The numbers of singleton families detected were 11,890 and 13,938 at the E-value of 10^−30^ and 10^−50^, respectively.
(49 KB PDF).

Figure S8 A Sample View of the H-Invitational Database (H-InvDB; http://www.h-invitational.jp/) A FLcDNA (BC003551) is shown with its detailed annotations, e.g., gene structure, functional annotation, ORF predictions, protein structure prediction by GTOP, etc. The H-InvDB has links to other internal databases (red boxes) such as a genome map viewer (G-integra) and gene expression library (H-Angel). Green boxes show internal viewers for the results of clustering (Clustering Viewer showing results by H-Inv, STACK, TIGR, UniGene, etc.), the prediction of subcellular localization (TOPOViewer showing results of TMHMM, SOSUI, TargetP, and PsortII), and the disease-related information (DiseaseInfo Viewer linking to OMIM and GenAtlas). The H-InvDB also has links to many external public databases (black boxes), including DDBJ/EMBL/GenBank, RefSeq, UniProt/Swiss-Prot and TrEMBL, Genew, InterPro, 3D Keynote, Ensembl, GeneLynx, LocusLink, PubMed, LIFEdb, dbSNP, GO, and GTOP, and to homepages by original data producers of FLcDNA clones and sequences (blue boxes), including the Chinese National Human Genome Center (CHGC), the Deutsches Krebsforschungszentrum (DKFZ/MIPS), Helix Research Institute (HRI), the Institute of Medical Science at the University of Tokyo (IMSUT), the Kazusa DNA Research Institute (KDRI), the Mammalian Gene Collection (MGC/NIH), and the FLJ project.
(2,650 KB PDF).

Figure S9 H-Inv Annotation Viewers (A) G-integra: A genome mapping viewer.
(B) SOUP Locus annotation viewer.
(C) SOUP cDNA annotation viewer.
(D) SMO Viewer: The similarity, motif, and ORF information viewer.
(2,022 KB PDF).

Table S1 Gene Structure (A) Gene structure of the cDNAs.
(B) The frequencies and varieties of repetitive sequences found in the cDNAs. A list of the 20,899 loci representing cDNAs that RepeatMasker showed contained repetitive elements.
(C) The positions (5′ UTR, ORF, and 3′ UTR) of repetitive sequences in the protein-coding cDNAs. A total of 1,863 cDNAs contained repetitive sequences in their ORF, of which 549 had repetitive sequences within their most probable ORF. Repetitive sequences appeared in 2,240 and 5,401 cDNAs in their 5′ UTRs and 3′ UTRs, respectively.
(20 KB PDF).

Table S2 CAI and Codon Usage (A) CAI was measured for all H-Inv proteins. CAI is a measure of biased patterns for synonymous codon usage (http://biobase.dk/embossdocs/cai.html).
(B) Codon usage in predicted ORFs of H-Inv proteins. Total tri-nucleotide frequencies (forward strand) for the sequences of each species are shown. Nonredundant proteome datasets for nonhuman species were obtained from the following sites: fly (Drosophila melanogaster; http://flybase.bio.indiana.edu/), worm (Caenorhabditis elegans; http://www.wormbase.org/), budding yeast (Saccharomyces cerevisiae; http://www.pasteur.fr/externe), fission yeast (Schizosaccharomyces pombe; http://www.sanger.ac.uk/), plant (Arabidopsis thaliana; http://mips.gsf.de/proj/thal/index.html), and bacteria (Escherichia coli K12; ftp://ftp.ncbi.nih.gov/genbank/genomes/Bacteria/Escherichia_coli_K12/).
(20 KB PDF).

Table S3 Tissue Library Origins of H-Inv Proteins The results of classification into five similarity categories for each of ten tissue classes.
(A) Numbers of H-Inv proteins.
(B) Histogram.
(10 KB PDF).

Table S4 The InterPro IDs Identified in H-Inv Proteins The top 40 InterPro IDs identified in H-Inv proteins and proteins from other species are listed for all types (A) and for each type of family, domain, and repeat (B–D). Analyses were conducted by InterPro ver. 3.1. Nonredundant proteome datasets of other species were obtained from the following sites: fly (Drosophila melanogaster; http://flybase.bio.indiana.edu/), worm (Caenorhabditis elegans; http://www.wormbase.org/), budding yeast (Saccharomyces cerevisiae; http://www.pasteur.fr/externe), fission yeast (Schizosaccharomyces pombe; http://www.sanger.ac.uk/), plant (Arabidopsis thaliana; http://mips.gsf.de/proj/thal/index.html), and bacteria (Escherichia coli K12; ftp://ftp.ncbi.nih.gov/genbank/genomes/Bacteria/Escherichia_coli_K12/).
(36 KB PDF).

Table S5 GO Term Assignment to H-Inv Proteins (A) Molecular function.
(B) Cellular component.
(C) Biological process.
(74 KB PDF).

Table S6 List of Newly Assigned Human Enzymes (32 H-Inv Proteins) All these 32 H-Inv proteins were newly assigned enzyme numbers with the support of the KEGG pathway. These enzyme assignments were previously unrepresented in *Homo sapiens.*
(33 KB PDF).

Table S7 A Functional Classification of Representative H-Inv cDNAs That Have Homologs in Other Species (See also Figure 6.)
(9 KB PDF).

Table S8 Basic Statistics for UTR Sequences Analyzed (8 KB PDF).

Table S9 UTR Replacements in Primates and Rodents One hundred and forty-seven UTR replacements distributed among different species were detected.
(9 KB PDF).

Table S10 List of the Databases and Software Used in the H-Inv Project (31 KB PDF).

Protocol S1 A Detailed Functional Annotation Based on Protein Modules (25 KB PDF).

## Abbreviations

3D: three-dimensional
AS: alternative splicing
CAI: codon adaptation index
dbSNP: Single Nucleotide Polymorphism Database
DDBJ: DNA Data Bank of Japan
EC: Enzyme Commission
EMBL: European Molecular Biology Laboratories
EST: expressed sequence tag
FANTOM: Functional Annotation of Mouse
FLcDNA: full-length cDNA
FLJ: Full-Length Long Japan
FTHFD: formyltetrahydrofolate dehydrogenase
GO: Gene Ontology
GTOP: Genomes TO Protein structures and functions database
H-Angel: Human Anatomic Gene Expression Library
H-Inv or H-Invitational: Human Full-Length cDNA Annotation Invitational
H-InvDB: H-Invitational Database
iAFLP: introduced amplified fragment length polymorphism
NCBI: National Center for Biotechnology Information
ncRNAs: non-protein-coding RNAs
OMIM: Online Mendelian Inheritance in Man
ORF: open reading frame
PDB: Protein Data Bank
RefSeq: Reference Sequence Collection
SMO: Similarity
SNP: single nucleotide polymorphism
