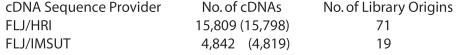# Correction: Integrative Annotation of 21,037 Human Genes Validated by Full-Length cDNA Clones

**DOI:** 10.1371/journal.pbio.0020256

**Published:** 2004-07-13

**In *PLoS Biology*, volume 2, issue 6:**

Integrative Annotation of 21,037 Human Genes Validated by Full-Length cDNA Clones

**Tadashi Imanishi, Takeshi Itoh, Yutaka Suzuki, Claire O'Donovan, Satoshi Fukuchi, et al.**

DOI: 10.1371/journal.pbio.0020162

1. The abbreviation FLJ was expanded incorrectly (as full-length long Japan) in the abbreviations list and in the first paragraph of the Results/Discussion section. FLJ stands for full-length cDNA Japan.

2. In Table 1, the number of cDNAs and the number of library origins for two of the cDNA sources were incorrect. The correct numbers are as follows:

**Table 1:**