# Supplementary material for: Integrative Annotation of 21,037 Human Genes Validated by Full-Length cDNA Clones
Source: PLoS Biol. 2004 Apr 20;2(6):e162. doi: 10.1371/journal.pbio.0020162 (PMC393292; doi:10.1371/journal.pbio.0020162)
Supplement: Figure S1 — (A) Schematic diagram for the prediction of ORFs. This diagram illustrates the ORF prediction method used on all H-Inv cDNAs. The method was based upon the alignment of similarity searches using FASTY and BLASTX. Gene prediction was carried out using GeneMark. Prior to the prediction of ORFs, we judged if a sequence had any frameshift errors or remaining introns. During ORF prediction, we corrected those sequence irregularities computationally. Details of how sequence irregularities were predicted are described in (B) and (C). (B) Schematic diagram for prediction of unspliced introns. This schematic diagram illustrates the prediction method used for unspliced introns. (C) Schematic diagram for prediction of frameshift errors. Frameshift errors were inferred from cDNA–genome pairwise alignment gaps due to insertion or deletion, exception of multiple of 3 bp, or over 10 bp in either the query cDNA or genome. (D) The statistics for the predicted frameshifts and unspliced introns. (49 KB PDF). [file pbio.0020162.sg001.pdf]

Figure S1. Prediction of ORFs

(A) Schematic diagram for the prediction of ORFs.

This diagram illustrates the ORF prediction method used on all H-Inv cDNAs. The method was based upon the alignment of similarity searches using FASTY and BLASTX. Gene prediction was carried out using GeneMark. Prior to the prediction of ORFs, we judged if a sequence had any frameshift errors or remaining introns. During ORF prediction, we corrected those sequence irregularities computationally. Details of how sequence irregularities were predicted are described in (B) and (C).

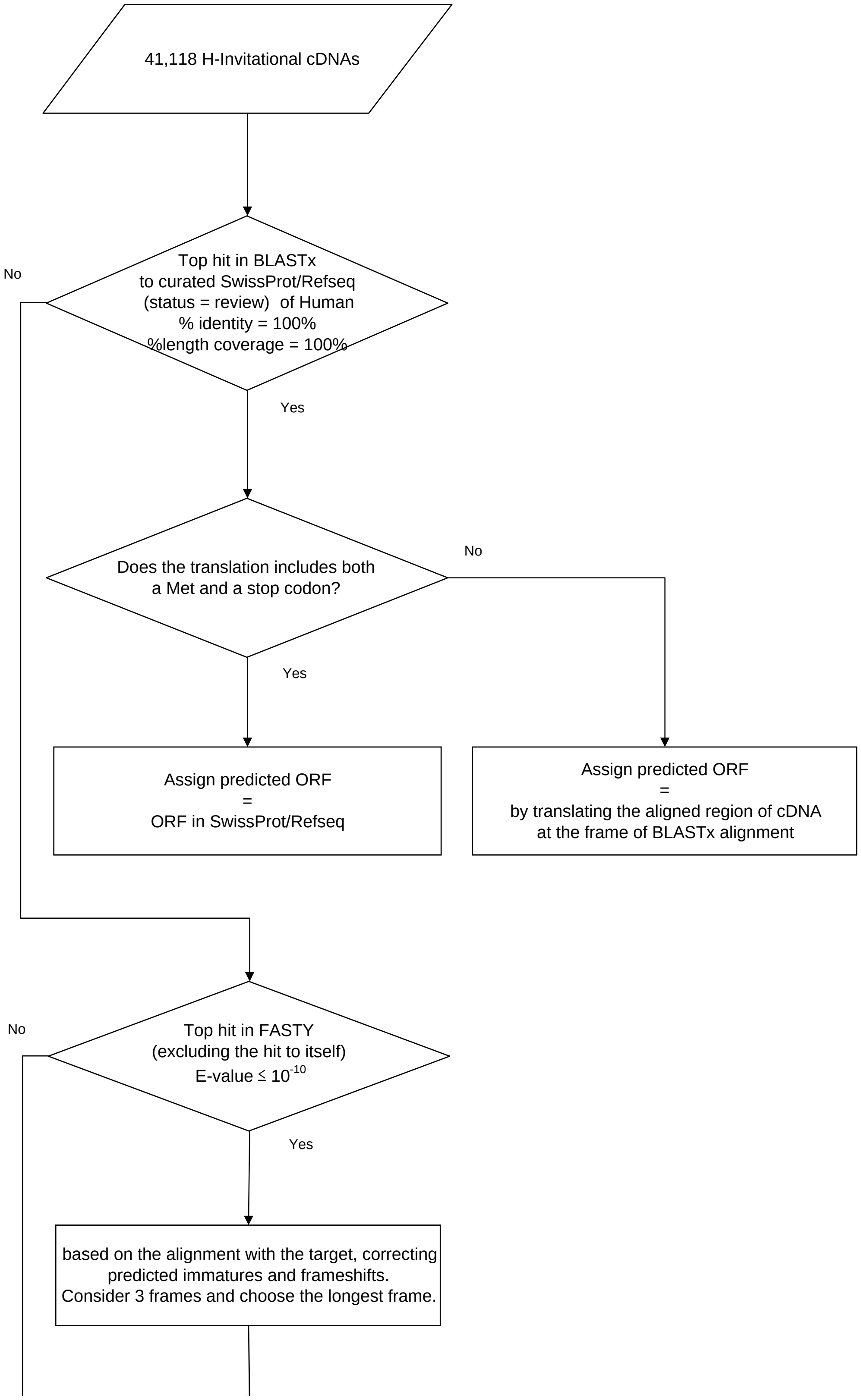

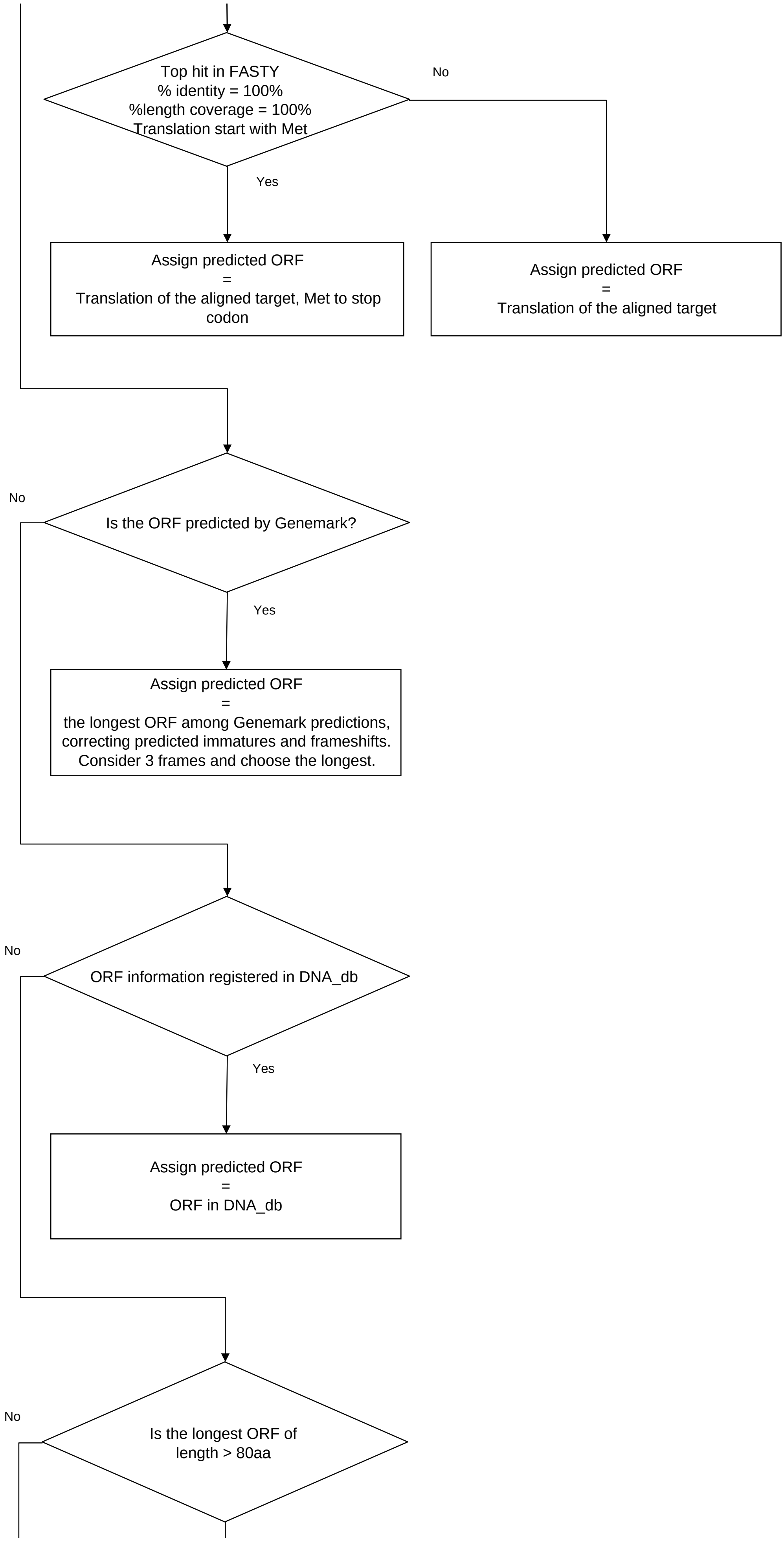
Top hit in FASTY
% identity = 100%
%length coverage = 100%
Translation start with Met
No
Yes
Assign predicted ORF
=
Translation of the aligned target, Met to stop codon
Assign predicted ORF
=
Translation of the aligned target
No
Is the ORF predicted by Genemark?
Yes
Assign predicted ORF
=
the longest ORF among Genemark predictions, correcting predicted immatures and frameshifts. Consider 3 frames and choose the longest.
No
ORF information registered in DNA_db
Yes
Assign predicted ORF
=
ORF in DNA_db
No
Is the longest ORF of length > 80aa

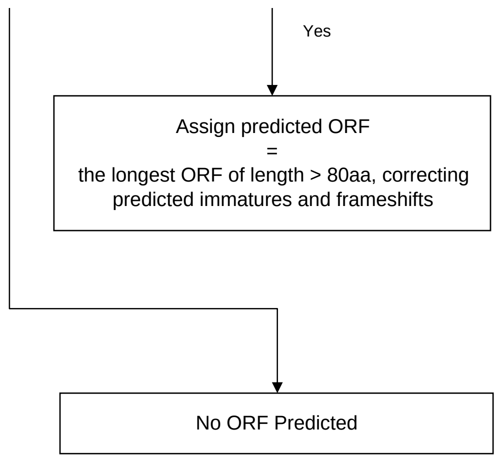
Yes
Assign predicted ORF
=
the longest ORF of length > 80aa, correcting predicted immatures and frameshifts
No ORF Predicted

Figure S1. Prediction of ORFs
(B) Schematic diagram for prediction of unspliced introns.

This schematic diagram illustrates the prediction method used for unspliced introns.

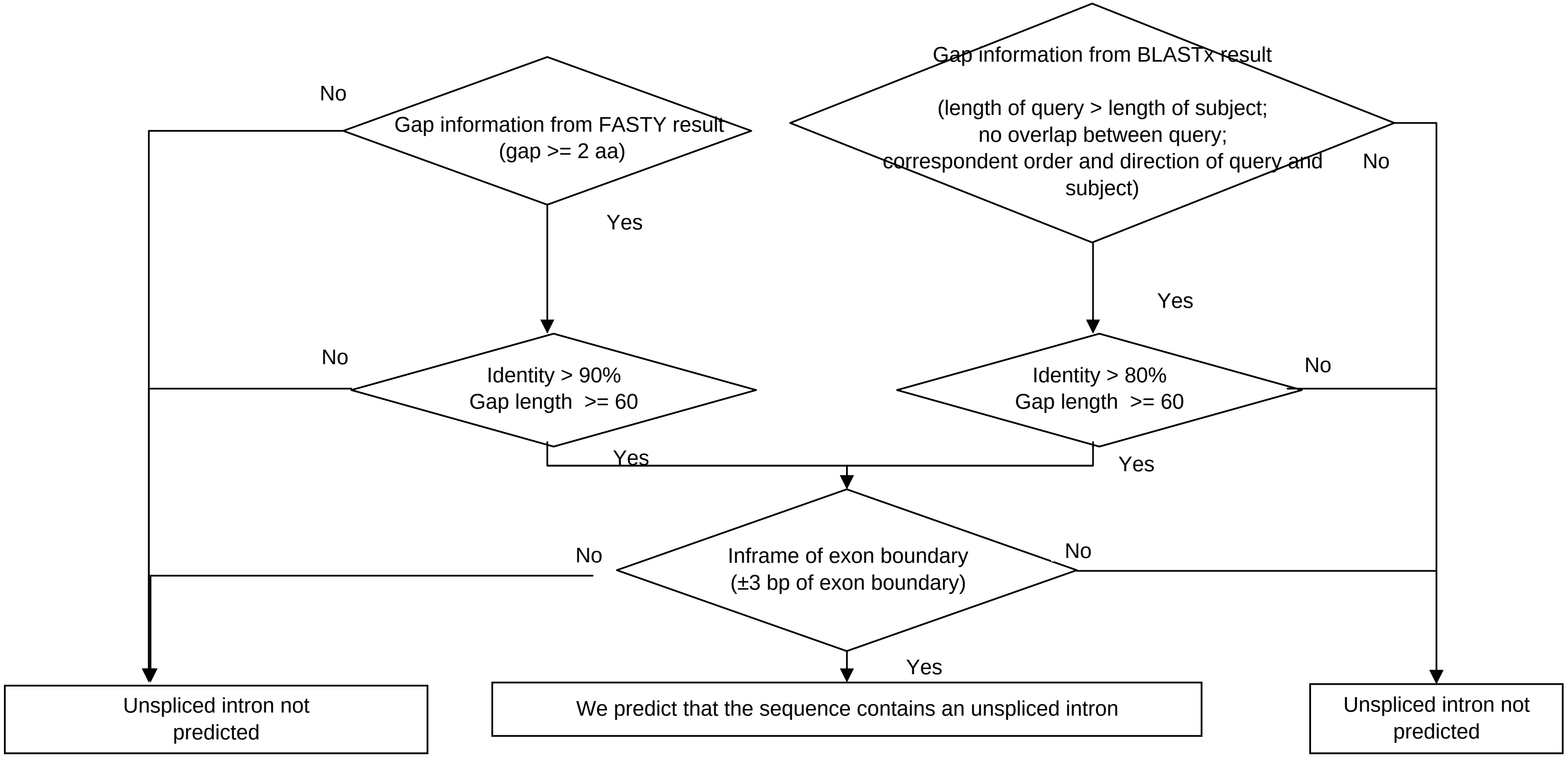

Figure S1. Prediction of ORFs

(C) Schematic diagram for prediction of frameshift errors.

Frameshift errors were inferred from cDNA–genome pairwise alignment gaps due to insertion or deletion, exception of multiple of 3 bp, or over 10 bp in either the query cDNA or genome.

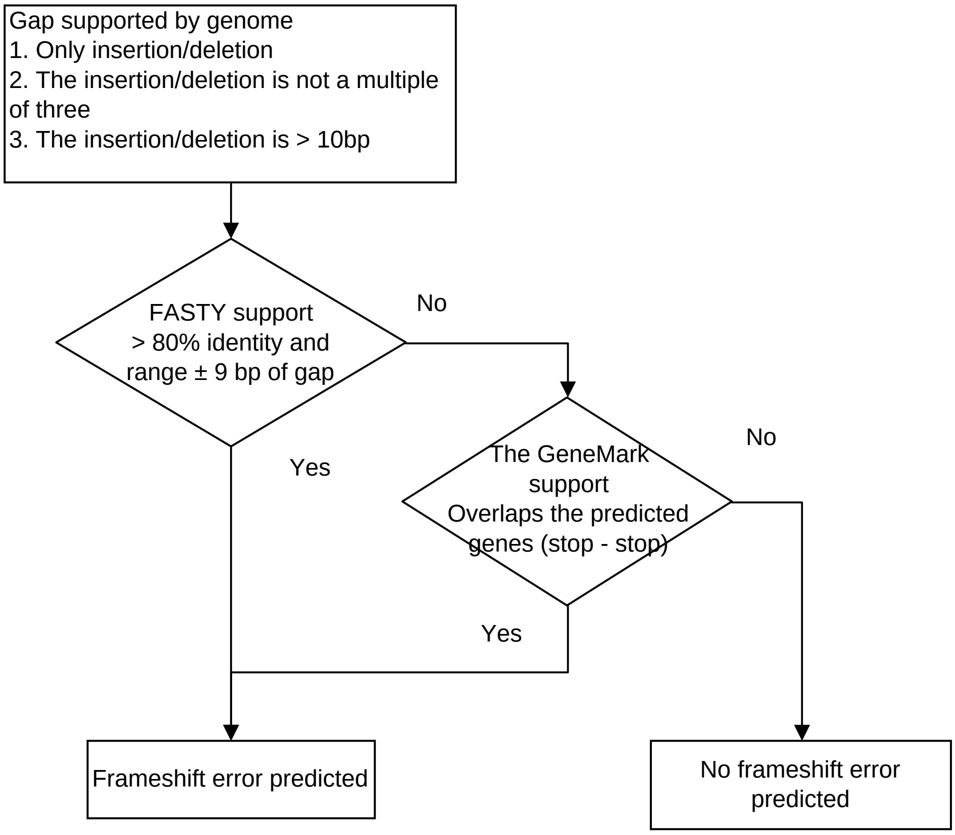

Figure S1. Prediction of ORFs

(D) The statistics for the predicted frameshifts and unspliced introns.

Irregularity	Category I protein	Category II protein	Category III protein	Category IV protein	Category V protein	Total
Frame shifts	48	117	71	60	38	372
Unspliced intron	290	456	131	87	46	1056
