# Supplementary material for: Integrative Annotation of 21,037 Human Genes Validated by Full-Length cDNA Clones
Source: PLoS Biol. 2004 Apr 20;2(6):e162. doi: 10.1371/journal.pbio.0020162 (PMC393292; doi:10.1371/journal.pbio.0020162)

Figure S2. Scheme of Prediction for Functional Annotation

(A) Schematic diagram for determining a representative transcript for each locus.

The procedure of computational autoannotation is illustrated. Prior to the human curation of the representative transcript of each H-Inv cluster, we performed computational autoannotation.

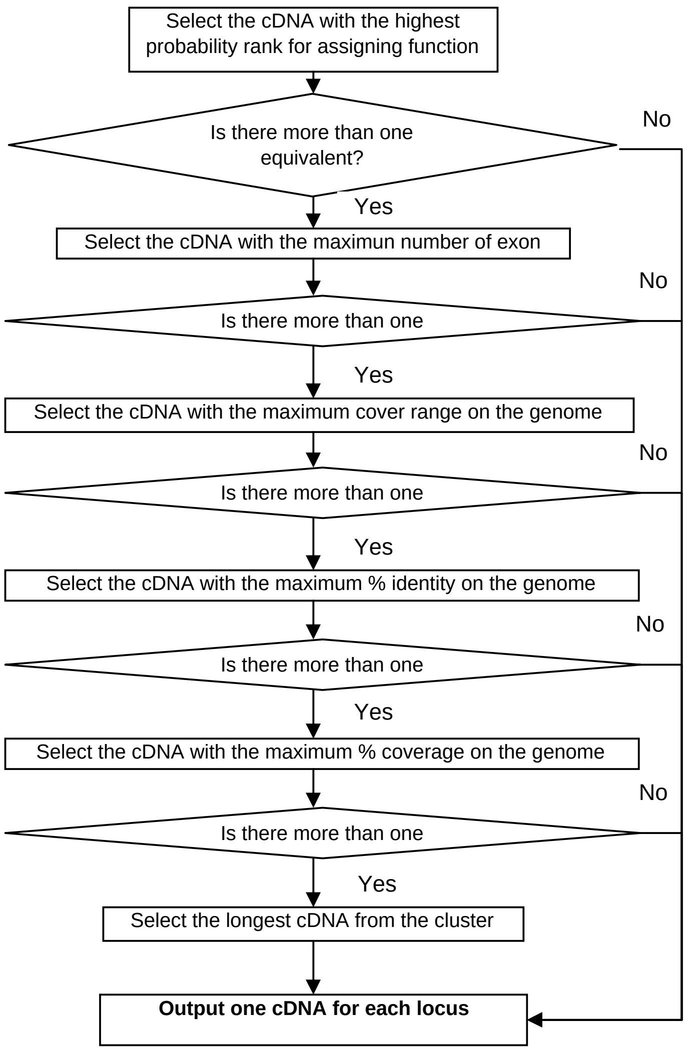

Figure S2. Scheme of Prediction for Functional Annotation

(B) Schematic diagram for functional prediction of H-Inv proteins.

This schematic diagram illustrates the H-Inv autofunctional annotation pipeline that can determine the most appropriate data source ID, avoiding the following keywords that suggest proteins without experimental verification in the description; (1) hypothetical, (2) similar to, (3) names of cDNA clones (Rik, KIAA, FLJ, DKFZ, HSPC, MGC, CHGC, and IMAGE) and (4) names of InterPro domain frequent hitters.

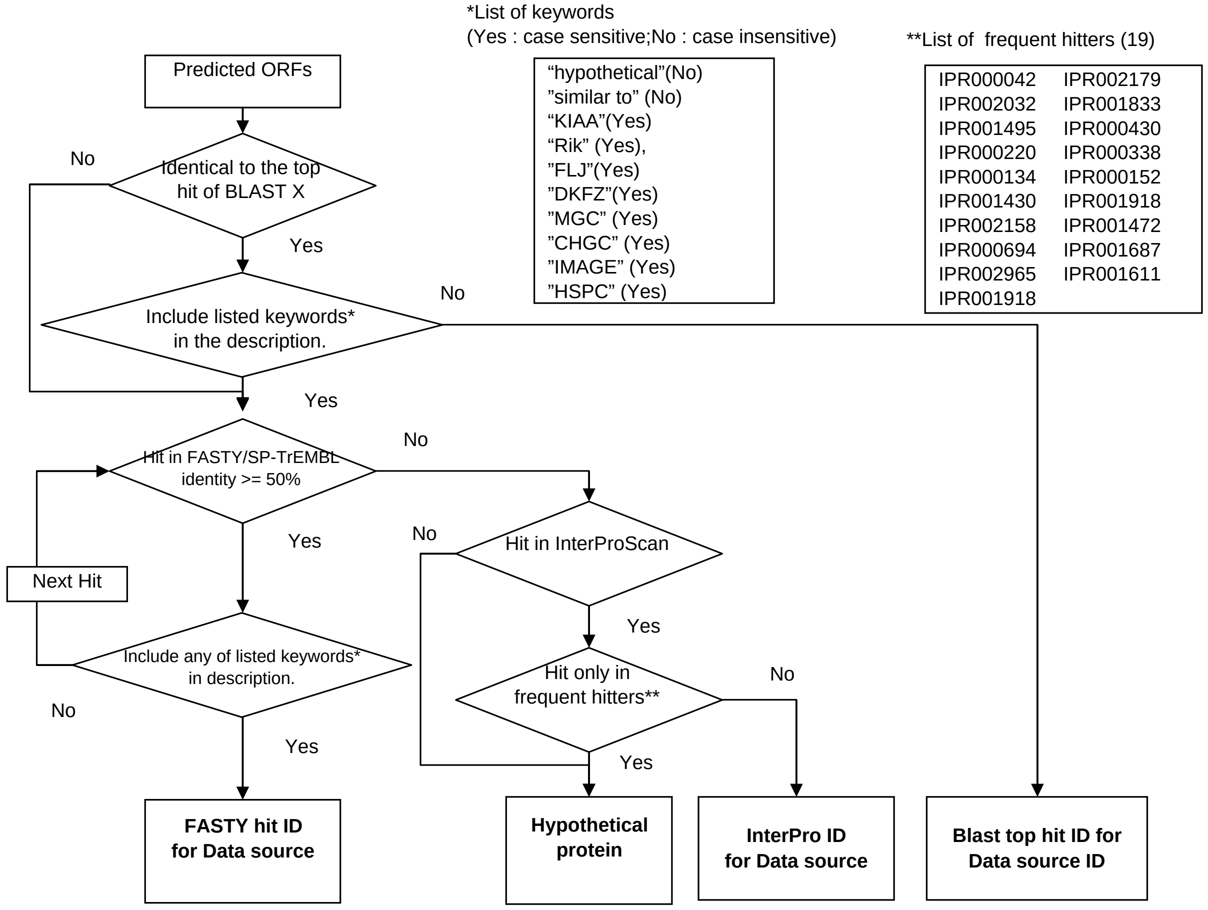

Supplement: Figure S2 — (A) Schematic diagram for determining a representative transcript for each locus. The procedure of computational autoannotation is illustrated. Prior to the human curation of the representative transcript of each H-Inv cluster, we performed computational autoannotation. (B) Schematic diagram for functional prediction of H-Inv proteins. This schematic diagram illustrates the H-Inv autofunctional annotation pipeline that can determine the most appropriate data source ID, avoiding the following keywords that suggest proteins without experimental verification in the description; (1) hypothetical, (2) similar to, (3) names of cDNA clones (Rik, KIAA, FLJ, DKFZ, HSPC, MGC, CHGC, and IMAGE) and (4) names of InterPro domain frequent hitters. (34 KB PDF). [file pbio.0020162.sg002.pdf]
