# Supplementary material for: Integrative Annotation of 21,037 Human Genes Validated by Full-Length cDNA Clones
Source: PLoS Biol. 2004 Apr 20;2(6):e162. doi: 10.1371/journal.pbio.0020162 (PMC393292; doi:10.1371/journal.pbio.0020162)
Supplement: Figure S3 — The size distribution of all H-Inv proteins among the five similarity categories. (24 KB PDF). [file pbio.0020162.sg003.pdf]

Figure S3. Size Distribution of Predicted ORFs

The size distribution of all H-Inv proteins among the five similarity categories.

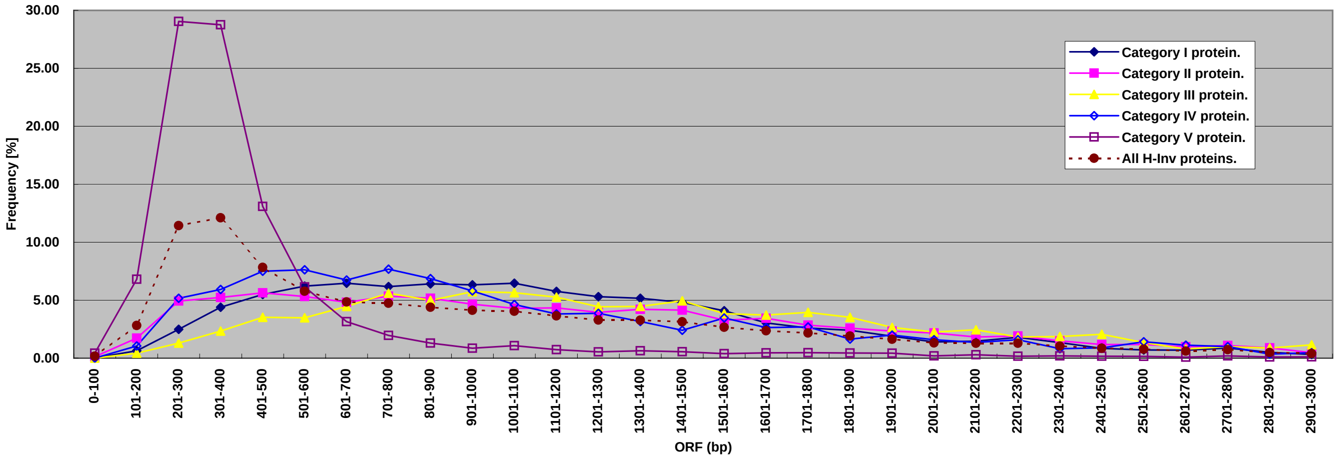

Size distribution of ORFs

No. of predicted ORFs						
Size of ORF (bp	Category I protei	Category II protei	ategory III prote	ategory IV prote	ategory V protei	All H-Inv protein
0-100	1	5	0	0	26	32
101-200	32	71	11	18	419	551
201-300	126	202	33	88	1789	2238
301-400	223	215	59	101	1771	2369
401-500	279	231	89	128	806	1533
501-600	315	218	88	130	378	1129
601-700	328	198	113	115	194	948
701-800	313	219	142	131	121	926
801-900	325	212	126	117	80	860
901-1000	321	191	145	99	53	809
1001-1100	328	176	143	79	66	792
1101-1200	292	178	133	65	45	713
1201-1300	269	162	112	66	33	642
1301-1400	262	173	113	54	39	641
1401-1500	244	170	125	41	34	614
1501-1600	207	134	97	59	24	521
1601-1700	154	141	94	45	28	462
1701-1800	132	117	100	46	29	424
1801-1900	122	106	89	28	27	372
1901-2000	96	96	67	34	26	319
2001-2100	71	89	57	27	12	256
2101-2200	74	75	62	23	18	252
2201-2300	91	78	46	27	10	252
2301-2400	68	61	47	14	12	202
2401-2500	43	48	52	15	10	168
2501-2600	36	48	34	24	9	151
2601-2700	35	40	20	19	5	119
2701-2800	44	45	25	17	12	143
2801-2900	25	36	22	6	6	95
2901-3000	16	19	29	8	7	79
3001-3100	17	32	23	10	7	89
3101-3200	22	36	16	6	4	84
3201-3300	21	20	20	7	5	73
3301-3400	17	32	17	2	4	72
3401-3500	12	24	12	4	5	57
3501-3600	12	13	18	0	6	49

% distribution of predicted ORFs						
Size of ORF (bp	Category I protei	Category II protei	ategory III prote	ategory IV prote	ategory V protei	All H-Inv protein
0-100	0.02	0.12	0.00	0.00	0.42	0.16
101-200	0.63	1.73	0.43	1.06	6.80	2.81
201-300	2.48	4.92	1.30	5.16	29.05	11.43
301-400	4.39	5.24	2.33	5.92	28.75	12.10
401-500	5.50	5.63	3.52	7.50	13.09	7.83
501-600	6.21	5.31	3.48	7.62	6.14	5.77
601-700	6.46	4.82	4.46	6.74	3.15	4.84
701-800	6.17	5.34	5.61	7.68	1.96	4.73
801-900	6.41	5.17	4.98	6.86	1.30	4.39
901-1000	6.33	4.65	5.73	5.80	0.86	4.13
1001-1100	6.46	4.29	5.65	4.63	1.07	4.05
1101-1200	5.75	4.34	5.25	3.81	0.73	3.64
1201-1300	5.30	3.95	4.43	3.87	0.54	3.28
1301-1400	5.16	4.22	4.46	3.17	0.63	3.27
1401-1500	4.81	4.14	4.94	2.40	0.55	3.14
1501-1600	4.08	3.27	3.83	3.46	0.39	2.66
1601-1700	3.04	3.44	3.71	2.64	0.45	2.36
1701-1800	2.60	2.85	3.95	2.70	0.47	2.17
1801-1900	2.40	2.58	3.52	1.64	0.44	1.90
1901-2000	1.89	2.34	2.65	1.99	0.42	1.63
2001-2100	1.40	2.17	2.25	1.58	0.19	1.31
2101-2200	1.46	1.83	2.45	1.35	0.29	1.29
2201-2300	1.79	1.90	1.82	1.58	0.16	1.29
2301-2400	1.34	1.49	1.86	0.82	0.19	1.03
2401-2500	0.85	1.17	2.05	0.88	0.16	0.86
2501-2600	0.71	1.17	1.34	1.41	0.15	0.77
2601-2700	0.69	0.97	0.79	1.11	0.08	0.61
2701-2800	0.87	1.10	0.99	1.00	0.19	0.73
2801-2900	0.49	0.88	0.87	0.35	0.10	0.49
2901-3000	0.32	0.46	1.15	0.47	0.11	0.40
3001-3100	0.34	0.78	0.91	0.59	0.11	0.45
3101-3200	0.43	0.88	0.63	0.35	0.06	0.43
3201-3300	0.41	0.49	0.79	0.41	0.08	0.37
3301-3400	0.34	0.78	0.67	0.12	0.06	0.37
3401-3500	0.24	0.58	0.47	0.23	0.08	0.29
3501-3600	0.24	0.32	0.71	0.00	0.10	0.25

3601-3700	16	16	15	4	5	56
3701-3800	10	8	10	4	1	33
3801-3900	10	15	10	5	4	44
3901-4000	6	15	15	4	1	41
4001-4100	2	10	10	5	2	29
4101-4200	7	18	4	3	3	35
4201-4300	3	6	5	3	2	19
4301-4400	4	10	12	2	3	31
4401-4500	4	9	8	2	2	25
4501-4600	4	7	10	1	2	24
4601-4700	2	1	10	3	0	16
4701-4800	2	4	9	2	1	18
4801-4900	2	10	0	2	0	14
4901-5000	2	7	4	0	1	14
5001-5100	3	3	5	1	1	13
5101-5200	0	3	2	0	3	8
5201-5300	1	6	2	2	0	11
5301-5400	1	10	3	1	1	16
5401-5500	2	3	1	1	1	8
5501-5600	1	2	1	1	0	5
5601-5700	1	2	3	0	1	7
5701-5800	2	3	0	2	0	7
5801-5900	0	5	3	1	0	9
5901-6000	3	2	1	0	1	7
6001-6100	2	1	0	1	0	4
6101-6200	2	3	2	0	3	10
6201-6300	0	5	2	0	0	7
6301-6400	0	0	1	0	0	1
6401-6500	0	1	0	1	0	2
6501-6600	0	0	0	0	0	0
6601-6700	2	1	0	1	0	4
6701-6800	0	1	0	0	0	1
6801-6900	0	1	0	0	0	1
6901-7000	1	0	0	0	0	1
7001-7100	0	0	0	0	0	0
7101-7200	1	0	0	0	0	1
7201-7300	0	1	1	0	0	2
7301-7400	0	0	0	0	0	0
7401-7500	0	0	1	0	0	1
7501-7600	0	1	0	0	0	1
7601-7700	1	0	0	0	0	1
7701-7800	1	0	1	0	0	2
7801-7900	0	0	0	0	1	1
7901-8000	2	0	0	0	0	2
8001-8100	0	0	0	0	0	0
8101-8200	0	0	0	0	0	0
8201-8300	0	0	0	0	0	0
8301-8400	0	1	0	0	0	1
8401-8500	0	1	0	0	0	1
8501-8600	0	0	0	0	0	0
8601-8700	0	0	0	0	0	0
8701-8800	0	0	0	0	0	0
8801-8900	0	0	0	0	0	0
8901-9000	0	0	0	0	0	0
9001-9100	0	0	0	0	0	0
9101-9200	0	0	0	0	0	0
9201-9300	0	0	0	1	0	1
9301-9400	0	0	0	0	0	0
9401-9500	0	0	0	0	0	0
9501-9600	0	0	0	0	0	0
9601-9700	0	0	0	0	0	0
9701-9800	0	0	0	0	0	0
9801-9900	1	0	0	0	0	1
9901-10000	0	1	0	0	0	1
10001-10100	0	0	0	0	0	0
10101-10200	0	0	1	0	0	1
Total	5074	4104	2531	1706	6159	19574

3601-3700	0.32	0.39	0.59	0.23	0.08	0.29
3701-3800	0.20	0.19	0.40	0.23	0.02	0.17
3801-3900	0.20	0.37	0.40	0.29	0.06	0.22
3901-4000	0.12	0.37	0.59	0.23	0.02	0.21
4001-4100	0.04	0.24	0.40	0.29	0.03	0.15
4101-4200	0.14	0.44	0.16	0.18	0.05	0.18
4201-4300	0.06	0.15	0.20	0.18	0.03	0.10
4301-4400	0.08	0.24	0.47	0.12	0.05	0.16
4401-4500	0.08	0.22	0.32	0.12	0.03	0.13
4501-4600	0.08	0.17	0.40	0.06	0.03	0.12
4601-4700	0.04	0.02	0.40	0.18	0.00	0.08
4701-4800	0.04	0.10	0.36	0.12	0.02	0.09
4801-4900	0.04	0.24	0.00	0.12	0.00	0.07
4901-5000	0.04	0.17	0.16	0.00	0.02	0.07
5001-5100	0.06	0.07	0.20	0.06	0.02	0.07
5101-5200	0.00	0.07	0.08	0.00	0.05	0.04
5201-5300	0.02	0.15	0.08	0.12	0.00	0.06
5301-5400	0.02	0.24	0.12	0.06	0.02	0.08
5401-5500	0.04	0.07	0.04	0.06	0.02	0.04
5501-5600	0.02	0.05	0.04	0.06	0.00	0.03
5601-5700	0.02	0.05	0.12	0.00	0.02	0.04
5701-5800	0.04	0.07	0.00	0.12	0.00	0.04
5801-5900	0.00	0.12	0.12	0.06	0.00	0.05
5901-6000	0.06	0.05	0.04	0.00	0.02	0.04
6001-6100	0.04	0.02	0.00	0.06	0.00	0.02
6101-6200	0.04	0.07	0.08	0.00	0.05	0.05
6201-6300	0.00	0.12	0.08	0.00	0.00	0.04
6301-6400	0.00	0.00	0.04	0.00	0.00	0.01
6401-6500	0.00	0.02	0.00	0.06	0.00	0.01
6501-6600	0.00	0.00	0.00	0.00	0.00	0.00
6601-6700	0.04	0.02	0.00	0.06	0.00	0.02
6701-6800	0.00	0.02	0.00	0.00	0.00	0.01
6801-6900	0.00	0.02	0.00	0.00	0.00	0.01
6901-7000	0.02	0.00	0.00	0.00	0.00	0.01
7001-7100	0.00	0.00	0.00	0.00	0.00	0.00
7101-7200	0.02	0.00	0.00	0.00	0.00	0.01
7201-7300	0.00	0.02	0.04	0.00	0.00	0.01
7301-7400	0.00	0.00	0.00	0.00	0.00	0.00
7401-7500	0.00	0.00	0.04	0.00	0.00	0.01
7501-7600	0.00	0.02	0.00	0.00	0.00	0.01
7601-7700	0.02	0.00	0.00	0.00	0.00	0.01
7701-7800	0.02	0.00	0.04	0.00	0.00	0.01
7801-7900	0.00	0.00	0.00	0.00	0.02	0.01
7901-8000	0.04	0.00	0.00	0.00	0.00	0.01
8001-8100	0.00	0.00	0.00	0.00	0.00	0.00
8101-8200	0.00	0.00	0.00	0.00	0.00	0.00
8201-8300	0.00	0.00	0.00	0.00	0.00	0.00
8301-8400	0.00	0.02	0.00	0.00	0.00	0.01
8401-8500	0.00	0.02	0.00	0.00	0.00	0.01
8501-8600	0.00	0.00	0.00	0.00	0.00	0.00
8601-8700	0.00	0.00	0.00	0.00	0.00	0.00
8701-8800	0.00	0.00	0.00	0.00	0.00	0.00
8801-8900	0.00	0.00	0.00	0.00	0.00	0.00
8901-9000	0.00	0.00	0.00	0.00	0.00	0.00
9001-9100	0.00	0.00	0.00	0.00	0.00	0.00
9101-9200	0.00	0.00	0.00	0.00	0.00	0.00
9201-9300	0.00	0.00	0.00	0.06	0.00	0.01
9301-9400	0.00	0.00	0.00	0.00	0.00	0.00
9401-9500	0.00	0.00	0.00	0.00	0.00	0.00
9501-9600	0.00	0.00	0.00	0.00	0.00	0.00
9601-9700	0.00	0.00	0.00	0.00	0.00	0.00
9701-9800	0.00	0.00	0.00	0.00	0.00	0.00
9801-9900	0.02	0.00	0.00	0.00	0.00	0.01
9901-10000	0.00	0.02	0.00	0.00	0.00	0.01
10001-10100	0.00	0.00	0.00	0.00	0.00	0.00
10101-10200	0.00	0.00	0.04	0.00	0.00	0.01
Total	100.00	100.00	100.00	100.00	100.00	100.00
