# Supplementary material for: Integrative Annotation of 21,037 Human Genes Validated by Full-Length cDNA Clones
Source: PLoS Biol. 2004 Apr 20;2(6):e162. doi: 10.1371/journal.pbio.0020162 (PMC393292; doi:10.1371/journal.pbio.0020162)

Figure S4. Features of Category II Proteins

A total of 4,104 H-Inv proteins were classified as Category II based on sequence similarity to functionally validated proteins. The table and figure show source species of proteins in public databases to which the Category II proteins were similar.

Category	No of cDNA
Mammal : Eukaryota & Metazoa & Mammalia	3,948
Animal : Eukaryota & Metazoa	124
Fungi : Eukaryota & Fungi	5
Plant : Eukaryota & Viridiplantae	7
Eukaryote : Eukaryota	7
Eubacteria : Bacteria	8
Archaebacteria : Archaea	0
Others	5
Total	4,104

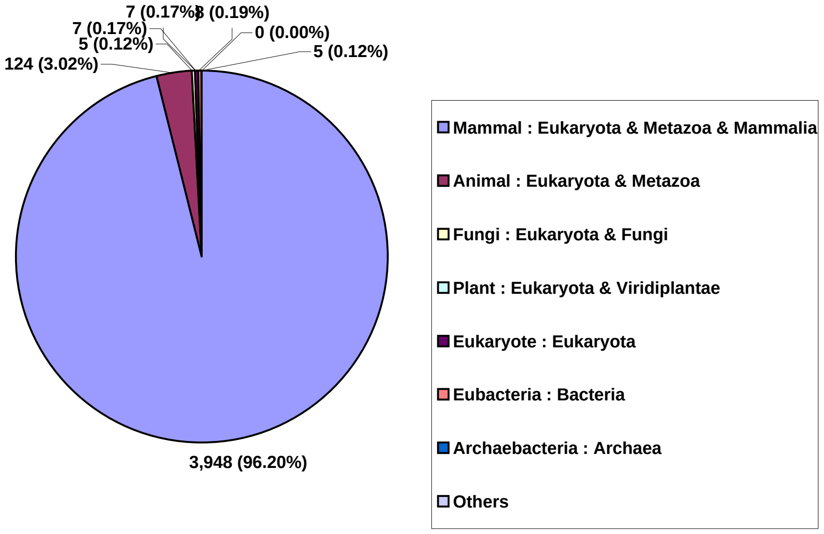

Supplement: Figure S4 — A total of 4,104 H-Inv proteins were classified as Category II based on sequence similarity to functionally validated proteins. The table and figure show source species of proteins in public databases to which the Category II proteins were similar. (9 KB PDF). [file pbio.0020162.sg004.pdf]
