# Supplementary material for: Integrative Annotation of 21,037 Human Genes Validated by Full-Length cDNA Clones
Source: PLoS Biol. 2004 Apr 20;2(6):e162. doi: 10.1371/journal.pbio.0020162 (PMC393292; doi:10.1371/journal.pbio.0020162)
Supplement: Figure S5 — The images illustrate the metabolic pathways of KEGG database based on the EC number assignments to H-Inv proteins. (47 KB PDF). [file pbio.0020162.sg005.pdf]

Figure S5.
H-Inv KEGG Analysis Results (Images of KEGG Pathways)

The images illustrate the metabolic pathways of KEGG database based on the EC number assignments to H-Inv proteins.

#1 Carbohydrate Metabolism

- 00010 Glycolysis / Gluconeogenesis
- 00020 Citrate cycle (TCA cycle)
- 00030 Pentose phosphate pathway
- 00031 Inositol metabolism
- 00040 Pentose and glucuronate interconversions
- 00051 Fructose and mannose metabolism
- 00052 Galactose metabolism
- 00053 Ascorbate and aldarate metabolism
- 00620 Pyruvate metabolism
- 00630 Glyoxylate and dicarboxylate metabolism
- 00640 Propanoate metabolism
- 00650 Butanoate metabolism
- 00660 C5-Branched dibasic acid metabolism

#2 Energy Metabolism

- 00190 Oxidative phosphorylation
- 00193 ATP synthesis
- 00195 Photosynthesis
- 00680 Methane metabolism
- 00710 Carbon fixation
- 00720 Reductive carboxylate cycle (CO2 fixation)
- 00910 Nitrogen metabolism
- 00920 Sulfur metabolism

#3 Lipid Metabolism

- 00061 Fatty acid biosynthesis (path 1)
- 00062 Fatty acid biosynthesis (path 2)
- 00071 Fatty acid metabolism
- 00072 Synthesis and degradation of ketone bodies
- 00100 Sterol biosynthesis
- 00120 Bile acid biosynthesis
- 00140 C21-Steroid hormone metabolism
- 00150 Androgen and estrogen metabolism

#4 Nucleotide Metabolism

- 00230 Purine metabolism
- 00240 Pyrimidine metabolism
- 00520 Nucleotide sugars metabolism

#5 Amino Acid Metabolism

- 00220 Urea cycle and metabolism of amino groups
- 00251 Glutamate metabolism
- 00252 Alanine and aspartate metabolism
- 00260 "Glycine, serine and threonine metabolism"
- 00271 Methionine metabolism
- 00272 Cysteine metabolism
- 00280 "Valine, le ucine and isoleucine degradation"
- 00290 "Valine, leucine and isoleucine biosynthesis"
- 00300 Lysine biosynthesis
- 00310 Lysine degradation
- 00330 Arginine and proline metabolism
- 00340 Histidine metabolism
- 00350 Tyrosine metabolism
- 00360 Phenylalanine metabolism
- 00380 Tryptophan metabolism
- 00400 "Phenylalanine, tyrosine and tryptophan biosynthesis"

#6 Metabolism of Other Amino Acids

- 00410 beta-Alanine metabolism
- 00430 Taurine and hypotaurine metabolism
- 00440 Aminophosphonate metabolism
- 00450 Selenoamino acid metabolism
- 00460 Cyanoamino acid metabolism
- 00471 D-Glutamine and D-glutamate metabolism
- 00472 D-Arginine and D-ornithine metabolism
- 00480 Glutathione metabolism

#7 Metabolism of Complex Carbohydrates

- 00500 Starch and sucrose metabolism
- 00510 N-Glycans biosynthesis
- 00512 O-Glycans biosynthesis
- 00530 Aminosugars metabolism
- 00531 Glycosaminoglycan degradation

- 00532 Chondroitin / Heparan sulfate biosynthesis
- 00533 Keratan sulfate biosynthesis
- 00550 Peptidoglycan biosynthesis

#8 Metabolism of Complex Lipids

- 00561 Glycerolipid metabolism
- 00562 Inositol phosphate metabolism
- 00563 Glycosylphosphatidylinositol(GPI)-anchor biosynthesis
- 00570 Sphingophospholipid biosynthesis
- 00580 Phospholipid degradation
- 00590 Prostaglandin and leukotriene metabolism
- 00600 Sphingoglycolipid metabolism
- 00601 Blood group glycolipid biosynthesis - lact series
- 00602 Blood group glycolipid biosynthesis - neolact series
- 00603 Globoside metabolism
- 00604 Ganglioside biosynthesis

#9 Metabolism of Cofactors and Vitamins

- 00130 Ubiquinone biosynthesis
- 00670 One carbon pool by folate
- 00730 Thiamine metabolism
- 00740 Riboflavin metabolism
- 00750 Vitamin B6 metabolism
- 00760 Nicotinate and nicotinamide metabolism
- 00770 Pantothenate and CoA biosynthesis
- 00780 Biotin metabolism
- 00790 Folate biosynthesis
- 00830 Retinol metabolism
- 00860 Porphyrin and chlorophyll metabolism

#10 Biosynthesis of Secondary Metabolites

- 00521 Streptomycin biosynthesis
- 00522 Erythromycin biosynthesis
- 00900 Terpenoid biosynthesis
- 00940 "Flavonoids, stilbene and lignin biosynthesis"
- 00950 Alkaloid biosynthesis I
- 00960 Alkaloid biosynthesis II

#11 Biodegradation of Xenobiotics

- 00351 "1,1,1-Trichloro-2,2-bis(4-chlorophenyl)ethane (DDT) degradation"

- 00361 gamma-Hexachlorocyclohexane degradation
- 00362 Benzoate degradation via hydroxylation
- 00625 Tetrachloroethene degradation
- 00626 Nitrobenzene degradation
- 00627 "1,4-Dichlorobenzene degradation"
- 00628 Fluorene degradation
- 00632 Benzoate degradation via CoA ligation
- 00642 Ethylbenzene degradation
- 00643 Styrene degradation

#12 Transcription

- 03020 RNA polymerase
- 03022 Transcription factors

#13 Translation

- 00970 Aminoacyl-tRNA biosynthesis
- 03010 Ribosome

#14 Sorting and Degradation

- 03050 Proteasome
- 03060 Protein export
- 03070 Type III secretion system
- 04120 Ubiquitin mediated proteolysis

#15 Replication and Repair

- 03030 DNA polymerase

#17 Signal Transduction

- 04010 MAPK signaling pathway
- 04070 Phosphatidylinositol signaling system

#18 Cell Growth and Death

- 04110 Cell cycle
- 04210 Apoptosis

#19 Behavior

- 04710 Circadian rhythm

#21 Cell Motility

- 02040 Flagellar assembly

#24 Neurodegenerative Disorders

- 01510 Neurodegenerative Disorders
- 05010 Alzheimer's disease
- 05020 Parkinson's disease
- 05030 Amyotrophic lateral sclerosis (ALS)
- 05040 Huntington's disease
- 05050 Dentatorubropallidoluysian atrophy (DRPLA)
- 05060 Prion disease
