# Supplementary material for: Integrative Annotation of 21,037 Human Genes Validated by Full-Length cDNA Clones
Source: PLoS Biol. 2004 Apr 20;2(6):e162. doi: 10.1371/journal.pbio.0020162 (PMC393292; doi:10.1371/journal.pbio.0020162)

Figure S6.

Numbers of Representative H-Inv cDNAs That Are Homologous to Proteins in Each Taxonomic Group

Two thresholds ($E < 10^{-5}$, white bars, and $E < 10^{-10}$, black bars) were employed. The "animal" group does not include mammalian species. The "eukaryote" group represents eukaryotic species other than animals, fungi, and plants.

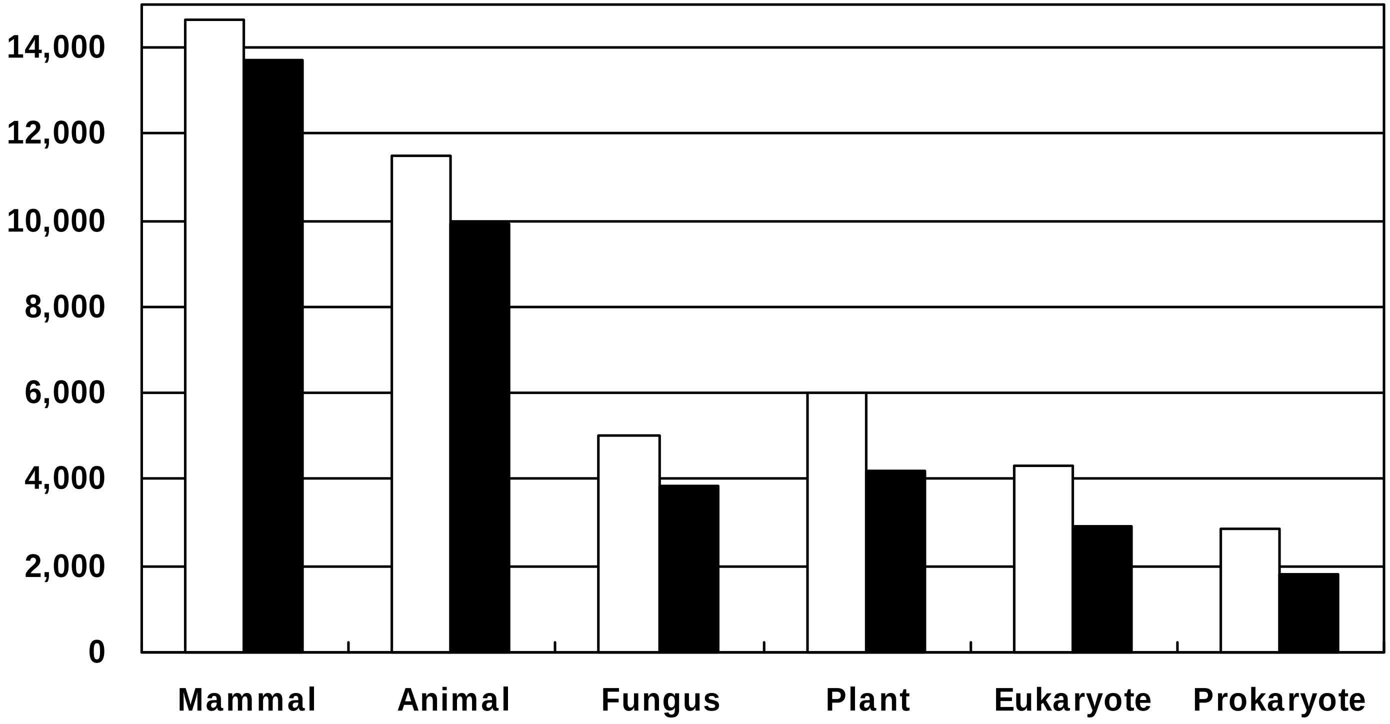

Supplement: Figure S6 — Two thresholds (E < 10−5, white bars, and E < 10−10, black bars) were employed. The “animal” group does not include mammalian species. The “eukaryote” group represents eukaryotic species other than animals, fungi, and plants. (9 KB PDF). [file pbio.0020162.sg006.pdf]
