# Supplementary material for: Integrative Annotation of 21,037 Human Genes Validated by Full-Length cDNA Clones
Source: PLoS Biol. 2004 Apr 20;2(6):e162. doi: 10.1371/journal.pbio.0020162 (PMC393292; doi:10.1371/journal.pbio.0020162)

Figure S7.

A Functional Classification of H-Inv Protein Families That Have Homologs in Each Taxonomic Group

H-Inv protein families were identified by clustering H-Inv proteins using the single-linkage clustering method. Then, the number of homologs for each H-Inv protein family was calculated. Mammalian species are excluded from the "animal" group. "eukaryote" represents eukaryotic species other than animals, fungi, and plants.

Single-linkage clustering. All of the H-Inv proteins were compared with themselves by BLASTP and clustered with the thresholds of E-values of 10^{-30} and 10^{-50}. The numbers of singleton families detected were 11,890 and 13,938 at the E-value of 10^{-30} and 10^{-50}, respectively.

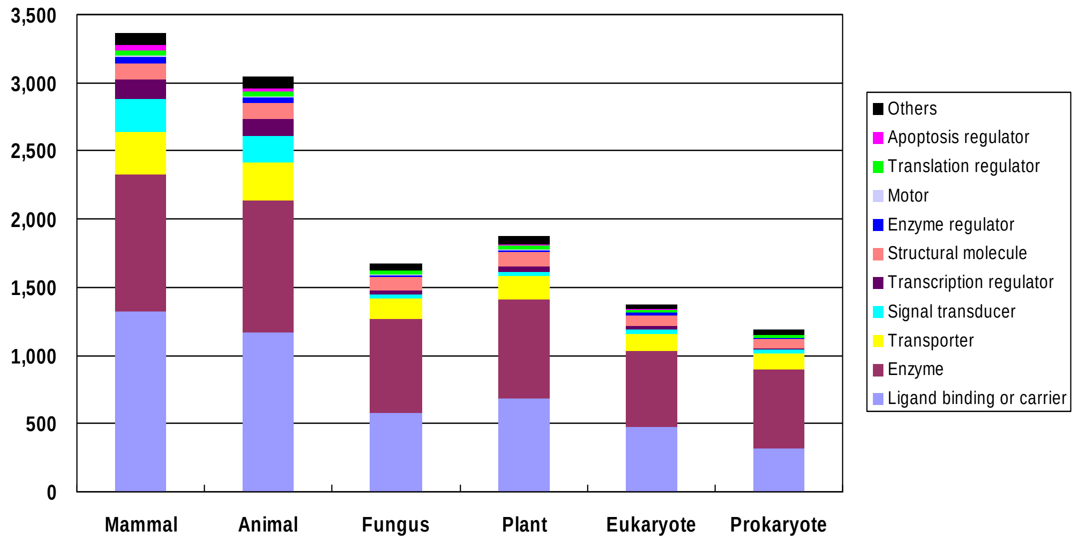

Supplement: Figure S7 — H-Inv protein families were identified by clustering H-Inv proteins using the single-linkage clustering method. Then, the number of homologs for each H-Inv protein family was calculated. Mammalian species are excluded from the “animal” group. “eukaryote” represents eukaryotic species other than animals, fungi, and plants. Single-linkage clustering. All of the H-Inv proteins were compared with themselves by BLASTP and clustered with the thresholds of E-values of 10−30 and 10−50. The numbers of singleton families detected were 11,890 and 13,938 at the E-value of 10−30 and 10−50, respectively. (49 KB PDF). [file pbio.0020162.sg007.pdf]
