# Supplementary material for: Integrative Annotation of 21,037 Human Genes Validated by Full-Length cDNA Clones
Source: PLoS Biol. 2004 Apr 20;2(6):e162. doi: 10.1371/journal.pbio.0020162 (PMC393292; doi:10.1371/journal.pbio.0020162)

Figure S8.
A Sample View of the H-Invitational Database (H-InvDB; http://www.h-invitational.jp/)

A FLcDNA (BC003551) is shown with its detailed annotations, e.g., gene structure, functional annotation, ORF predictions, protein structure prediction by GTOP, etc. The H-InvDB has links to other internal databases (red boxes) such as a genome map viewer (G-integra) and gene expression library (H-Angel). Green boxes show internal viewers for the results of clustering (Clustering Viewer showing results by H-Inv, STACK, TIGR, UniGene, etc.), the prediction of subcellular localization (TOPOViewer showing results of TMHMM, SOSUI, TargetP, and PsortII), and the disease-related information (DiseaseInfo Viewer linking to OMIM and GenAtlas). The H-InvDB also has links to many external public databases (black boxes), including DDBJ/EMBL/GenBank, RefSeq, UniProt/Swiss-Prot and TrEMBL, Genew, InterPro, 3D Keynote, Ensembl, GeneLynx, LocusLink, PubMed, LIFEdb, dbSNP, GO, and GTOP, and to homepages by original data producers of FLcDNA clones and sequences (blue boxes), including the Chinese National Human Genome Center (CHGC), the Deutsches Krebsforschungszentrum (DKFZ/MIPS), Helix Research Institute (HRI), the Institute of Medical Science at the University of Tokyo (IMSUT), the Kazusa DNA Research Institute (KDRI), the Mammalian Gene Collection (MGC/NIH), and the FLJ project.

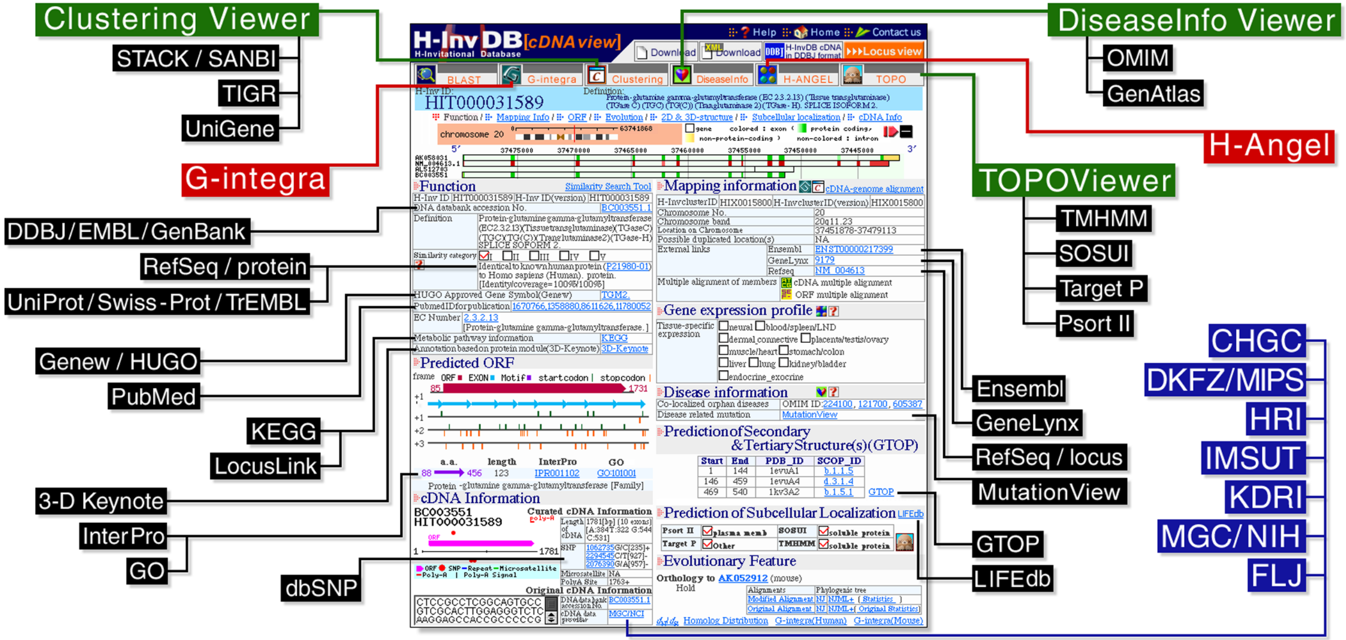

Supplement: Figure S8 — A FLcDNA (BC003551) is shown with its detailed annotations, e.g., gene structure, functional annotation, ORF predictions, protein structure prediction by GTOP, etc. The H-InvDB has links to other internal databases (red boxes) such as a genome map viewer (G-integra) and gene expression library (H-Angel). Green boxes show internal viewers for the results of clustering (Clustering Viewer showing results by H-Inv, STACK, TIGR, UniGene, etc.), the prediction of subcellular localization (TOPOViewer showing results of TMHMM, SOSUI, TargetP, and PsortII), and the disease-related information (DiseaseInfo Viewer linking to OMIM and GenAtlas). The H-InvDB also has links to many external public databases (black boxes), including DDBJ/EMBL/GenBank, RefSeq, UniProt/Swiss-Prot and TrEMBL, Genew, InterPro, 3D Keynote, Ensembl, GeneLynx, LocusLink, PubMed, LIFEdb, dbSNP, GO, and GTOP, and to homepages by original data producers of FLcDNA clones and sequences (blue boxes), including the Chinese National Human Genome Center (CHGC), the Deutsches Krebsforschungszentrum (DKFZ/MIPS), Helix Research Institute (HRI), the Institute of Medical Science at the University of Tokyo (IMSUT), the Kazusa DNA Research Institute (KDRI), the Mammalian Gene Collection (MGC/NIH), and the FLJ project. (2,650 KB PDF). [file pbio.0020162.sg008.pdf]
