# Supplementary material for: Integrative Annotation of 21,037 Human Genes Validated by Full-Length cDNA Clones
Source: PLoS Biol. 2004 Apr 20;2(6):e162. doi: 10.1371/journal.pbio.0020162 (PMC393292; doi:10.1371/journal.pbio.0020162)

Figure S9. H-Inv Annotation Viewers

(A) G-integra: A gemone mapping viewer.

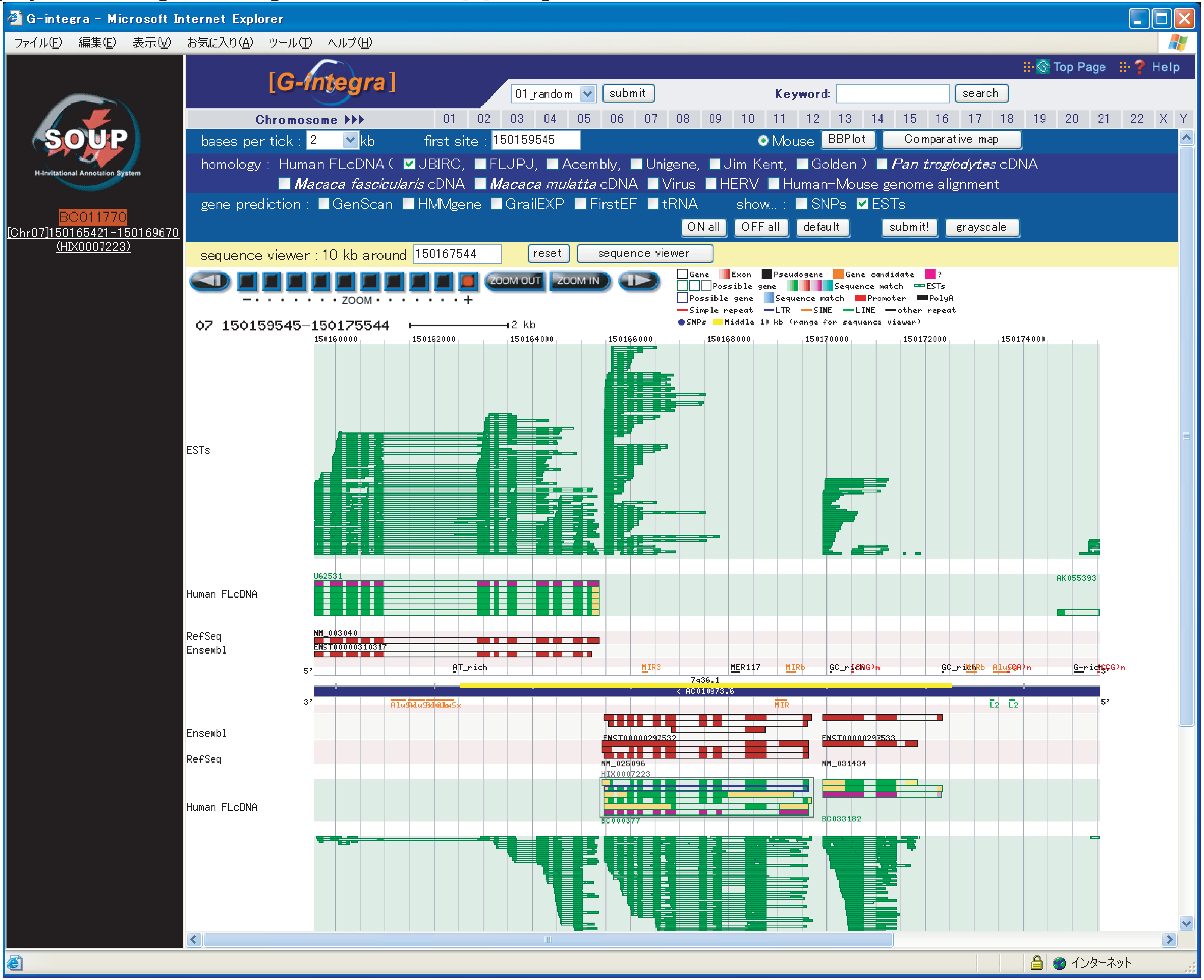

Figure S9. H-Inv Annotation Viewers

(B) SOUP Locus annotation Viewer.

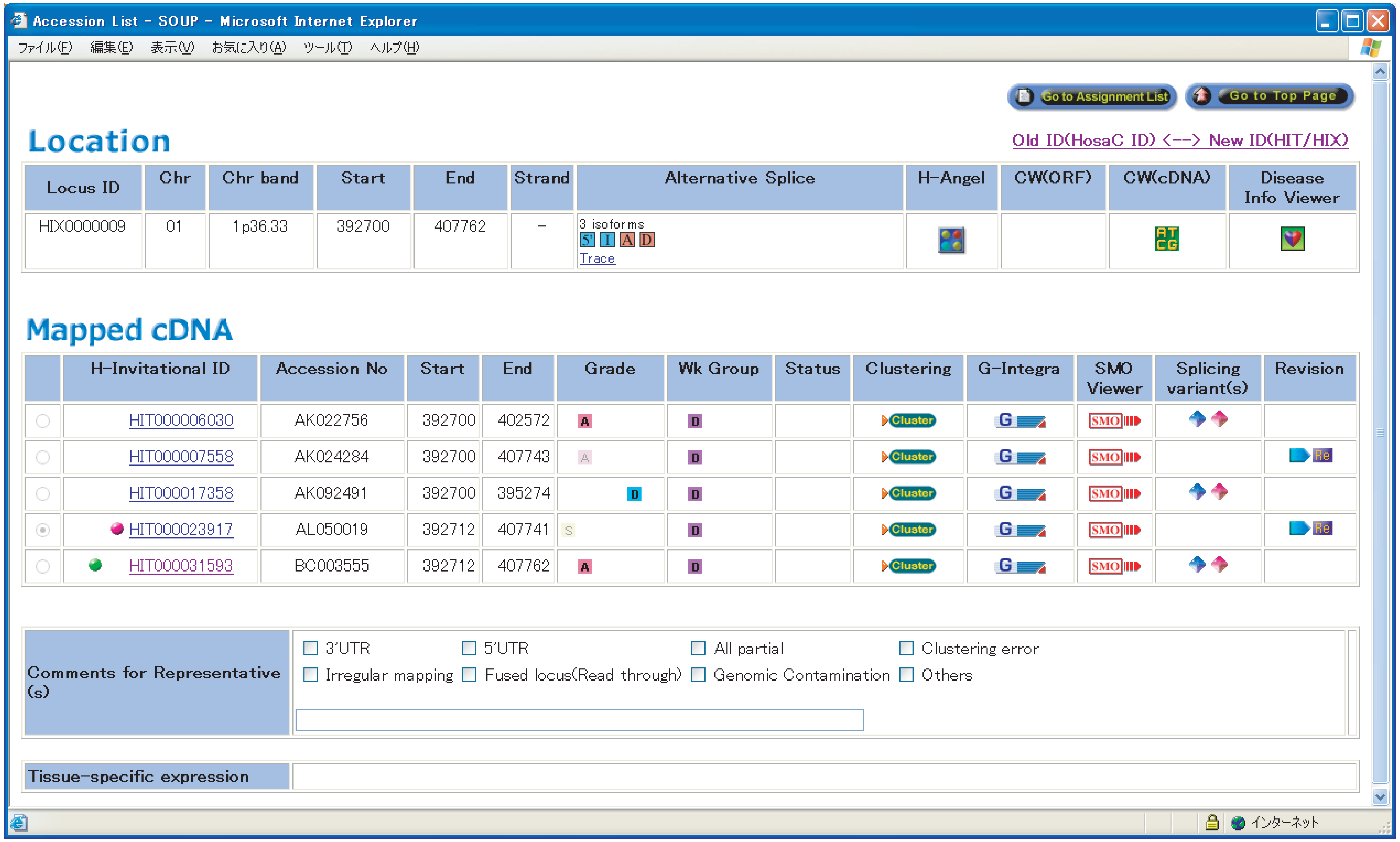

Figure S9. H-Inv Annotation Viewers

(C) SOUP cDNA annotation Viewer.

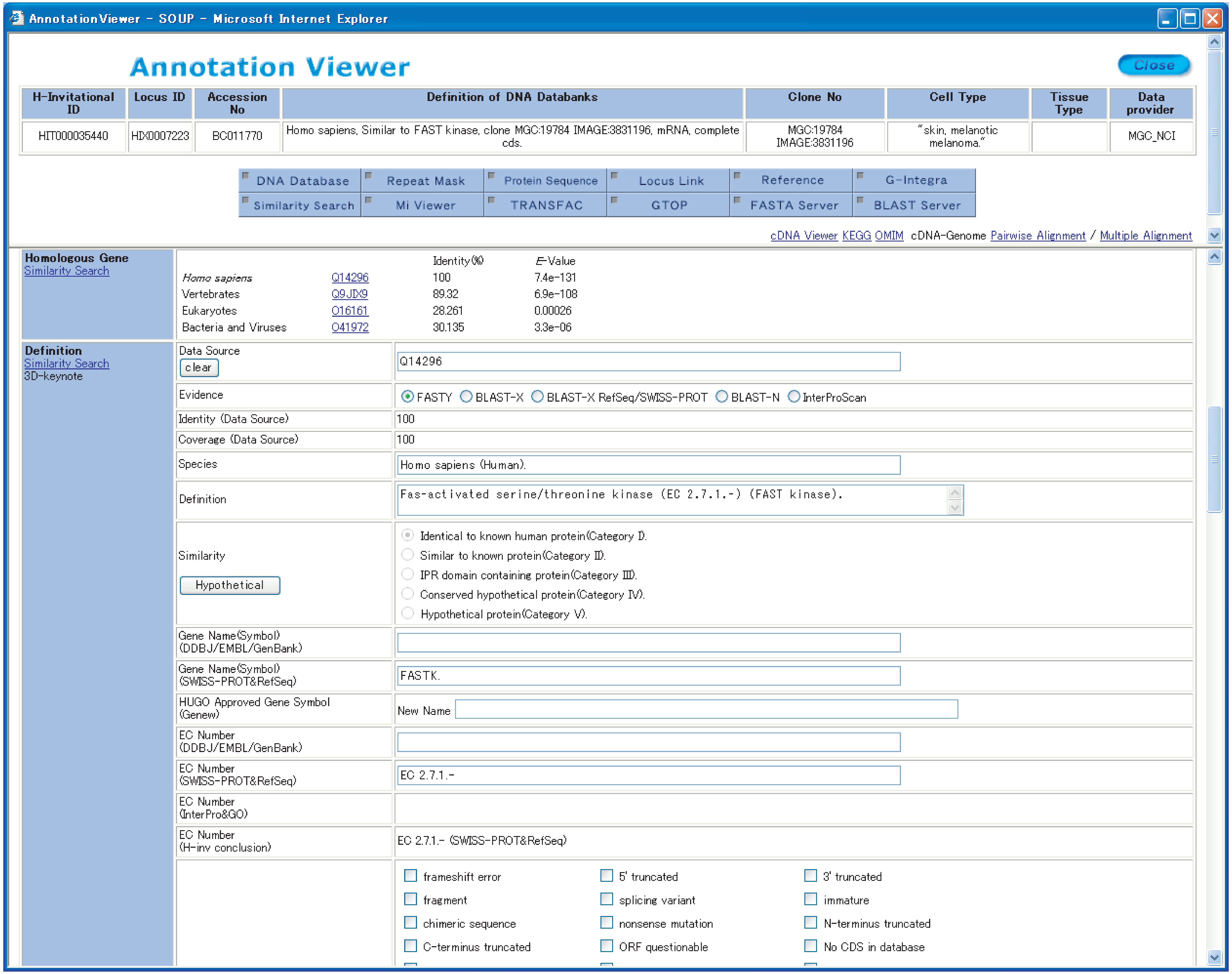

Figure S9. H-Inv Annotation Viewers

(D) SMOViewer: The similarity, motif and ORF information viewer.

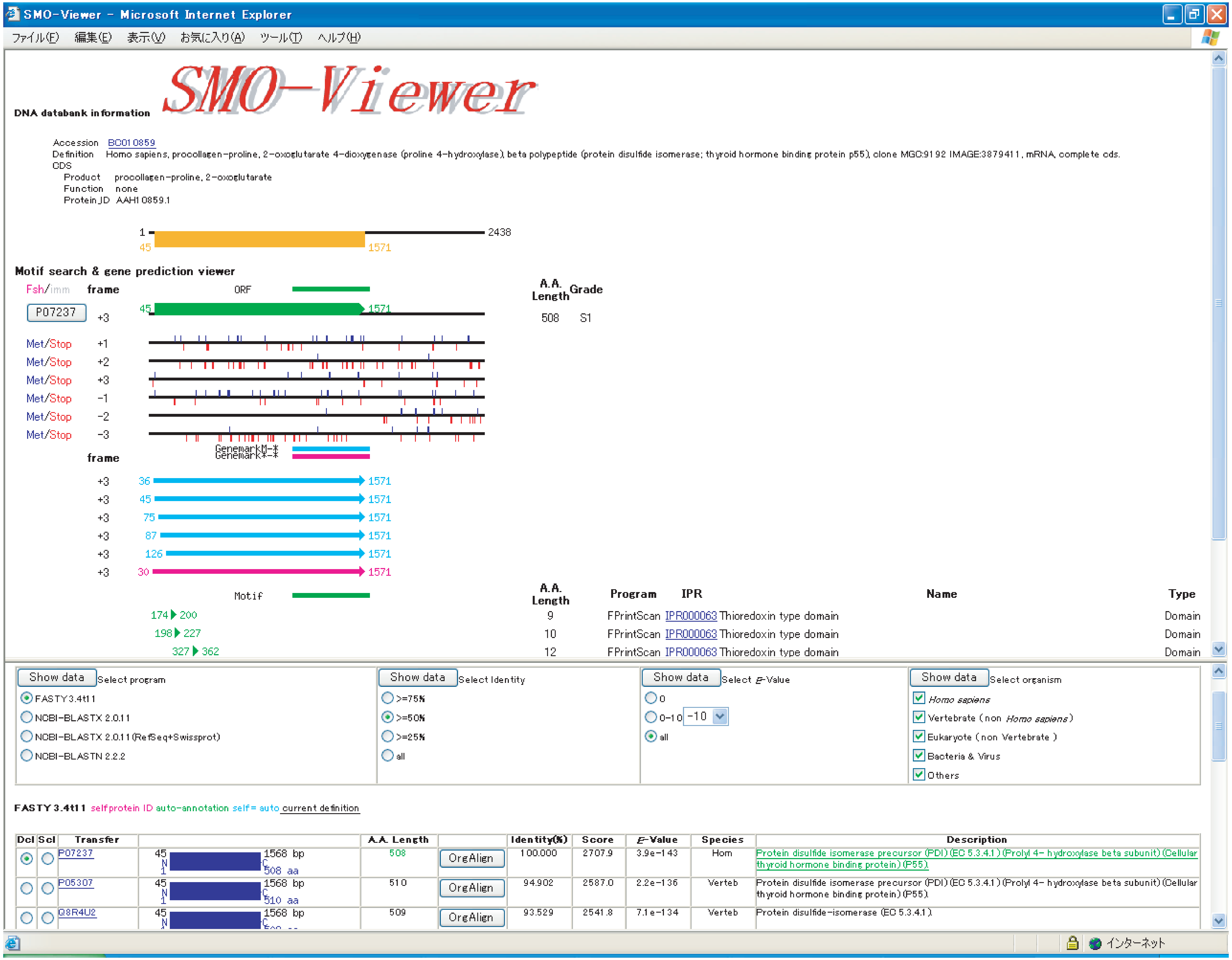

Supplement: Figure S9 — (A) G-integra: A genome mapping viewer. (B) SOUP Locus annotation viewer. (C) SOUP cDNA annotation viewer. (D) SMO Viewer: The similarity, motif, and ORF information viewer. (2,022 KB PDF). [file pbio.0020162.sg009.pdf]
