# Supplementary material for: Integrative Annotation of 21,037 Human Genes Validated by Full-Length cDNA Clones
Source: PLoS Biol. 2004 Apr 20;2(6):e162. doi: 10.1371/journal.pbio.0020162 (PMC393292; doi:10.1371/journal.pbio.0020162)
Supplement: Table S1 — (A) Gene structure of the cDNAs. (B) The frequencies and varieties of repetitive sequences found in the cDNAs. A list of the 20,899 loci representing cDNAs that RepeatMasker showed contained repetitive elements. (C) The positions (5′ UTR, ORF, and 3′ UTR) of repetitive sequences in the protein-coding cDNAs. A total of 1,863 cDNAs contained repetitive sequences in their ORF, of which 549 had repetitive sequences within their most probable ORF. Repetitive sequences appeared in 2,240 and 5,401 cDNAs in their 5′ UTRs and 3′ UTRs, respectively. (20 KB PDF). [file pbio.0020162.st001.pdf]

Table S1. Gene Structure

(A) Gene structure of the cDNAs.

	Median	Mean ± s.d.
Number of exons	5	7 ± 7
Extent of the genome (bp)	10,813	35,071 ± 72,869
The length of the first exons (bp)	148	251 ± 360
The length of the internal exons (bp)	122	153 ± 182
The length of the last exons (bp)	786	1,076 ± 946
The length of the first introns (bp)	3,152	11,891 ± 27,543
The length of the internal introns (bp)	1,436	4,676 ± 14,417
The length of the last introns (bp)	1,352	4,127 ± 12,651

Table S1. Gene Structure

(B) The frequencies and varieties of repetitive sequences found in the cDNAs.

A list of the 20,899 loci representing cDNAs that RepeatMasker showed contained repetitive elements.

Repeats	Number of cDNAs
SINE/Alu	6,093
SINE/MIR	2,826
LINE/L1	2,465
LINE/L2	1,827
LTR/MaLR	1,411
DNA/MER1_type	1,279
LTR/ERV1	934
LTR/ERVL	503
DNA/MER2_type	434
LINE/CR1	270
DNA/AcHobo	136
DNA/Tip100	105
LTR/ERVK	95
DNA/Mariner	95
DNA	85
DNA/MER1_type?	54
snRNA	35
DNA/Tc2	33
Satellite	27
Other	27
tRNA	23
Satellite/centr	17
scRNA	16
DNA/T2_type	14
srpRNA	12
LTR/ERV	9
rRNA	8
Satellite/telo	8
Unknown	6
RNA	4

Table S1. Gene Structure

(C) The positions (5' UTR, ORF, 3' UTR) of repetitive sequences in the protein-coding cDNAs.

A total of 1,863 cDNAs contained repetitive sequences in their ORF, of which 549 had repetitive sequences within their most probable ORF. Repetitive sequences appeared in 2,240 and 5,401 cDNAs in their 5' UTRs and 3' UTRs, respectively.

5' UTR			ORF			3' UTR		
Repeats	Number of repeats	%	Repeats	Number of repeats	%	Repeats	Number of repeats	%
SINE/Alu	428	21.5	LINE/L1	223	20.3	SINE/Alu	2,824	42.9
SINE/MIR	322	16.2	SINE/Alu	217	19.8	SINE/MIR	819	12.5
LINE/L1	304	15.3	LTR/MaLR	122	11.1	LINE/L1	758	11.5
LTR/MaLR	200	10.1	LTR/ERV1	118	10.8	LINE/L2	469	7.1
LINE/L2	189	9.5	LINE/L2	107	9.8	LTR/MaLR	407	6.2
LTR/ERV1	175	8.8	SINE/MIR	105	9.6	LTR/ERV1	371	5.6
DNA/MER1_type	114	5.7	LTR/ERVL	50	4.6	DNA/MER1_type	310	4.7
LTR/ERVL	70	3.5	DNA/MER1_type	44	4.0	LTR/ERVL	159	2.4
DNA/MER2_type	49	2.5	LTR/ERVK	30	2.7	DNA/MER2_type	134	2.0
LTR/ERVK	34	1.7	DNA/MER2_type	28	2.6	LINE/CR1	62	0.9
LINE/CR1	18	0.9	LINE/CR1	10	0.9	LTR/ERVK	59	0.9
DNA/AcHobo	16	0.8	Other	6	0.5	DNA/AcHobo	32	0.5
Satellite	8	0.4	DNA/Tip100	5	0.5	DNA/Tip100	31	0.5
DNA/Mariner	8	0.4	DNA/Mariner	5	0.5	DNA/Mariner	29	0.4
DNA/MER1_type?	7	0.4	DNA/AcHobo	5	0.5	DNA	25	0.4
DNA	7	0.4	Satellite/centr	4	0.4	DNA/MER1_type?	22	0.3
snRNA	6	0.3	Satellite	4	0.4	snRNA	15	0.2
Satellite/centr	5	0.3	DNA/T2_type	3	0.3	Other	13	0.2
Satellite/telo	4	0.2	snRNA	2	0.2	DNA/Tc2	11	0.2
Other	4	0.2	Satellite/telo	2	0.2	tRNA	5	0.1
DNA/Tc2	4	0.2	tRNA	1	0.1	Satellite	4	0.1
DNA/T2_type	4	0.2	srpRNA	1	0.1	srpRNA	3	0.0
tRNA	3	0.2	scRNA	1	0.1	scRNA	3	0.0
DNA/Tip100	3	0.2	DNA/Tc2	1	0.1	Satellite/centr	3	0.0
Unknown	2	0.1	DNA/MER1_type?	1	0.1	RNA	2	0.0
srpRNA	1	0.1	DNA	1	0.1	LTR/ERV	2	0.0
scRNA	1	0.1				DNA/T2_type	2	0.0
rRNA	1	0.1				rRNA	1	0.0
						Satellite/telo	1	0.0
