# Supplementary material for: Integrative Annotation of 21,037 Human Genes Validated by Full-Length cDNA Clones
Source: PLoS Biol. 2004 Apr 20;2(6):e162. doi: 10.1371/journal.pbio.0020162 (PMC393292; doi:10.1371/journal.pbio.0020162)
Supplement: Table S2 — (A) CAI was measured for all H-Inv proteins. CAI is a measure of biased patterns for synonymous codon usage (http://biobase.dk/embossdocs/cai.html). (B) Codon usage in predicted ORFs of H-Inv proteins. Total tri-nucleotide frequencies (forward strand) for the sequences of each species are shown. Nonredundant proteome datasets for nonhuman species were obtained from the following sites: fly (Drosophila melanogaster; http://flybase.bio.indiana.edu/), worm (Caenorhabditis elegans; http://www.wormbase.org/), budding yeast (Saccharomyces cerevisiae; http://www.pasteur.fr/externe), fission yeast (Schizosaccharomyces pombe; http://www.sanger.ac.uk/), plant (Arabidopsis thaliana; http://mips.gsf.de/proj/thal/index.html), and bacteria (Escherichia coli K12; ftp://ftp.ncbi.nih.gov/genbank/genomes/Bacteria/Escherichia_coli_K12/). (20 KB PDF). [file pbio.0020162.st002.pdf]

Table S2. CAI and Codon Usage

(A) CAI was measured for all H-Inv proteins.

CAI is a measure of biased patterns for synonymous codon usage (http://biobase.dk/embossdocs/cai.html).

Distribution in similarity categories.

	Median	Mean	Min	Max	Number of ORFs
Category I proteins	0.783	0.780	0.648	0.923	5,074
Category II proteins	0.770	0.770	0.604	0.918	4,104
Category III proteins	0.770	0.769	0.638	0.954	2,531
Category IV proteins	0.760	0.760	0.610	0.900	1,706
Category V proteins	0.741	0.742	0.586	0.933	6,159
Total	0.761	0.763	0.586	0.954	19,574

Distribution in 10 tissue classes for genes showing tissue-specific expression.

Tissues	Median	Mean	Min	Max	Number of ORFs
Neural	0.791	0.792	0.693	0.887	61
Blood/spleen/LND	0.795	0.786	0.652	0.954	261
Dermal-connective	0.791	0.782	0.682	0.871	93
Placenta/testis/ovary	0.782	0.785	0.690	0.887	46
Muscle/heart	0.776	0.771	0.650	0.864	135
Stomach/colon	0.789	0.789	0.648	0.896	110
Liver	0.790	0.784	0.709	0.846	32
Lung	0.781	0.781	0.683	0.848	97
Kidney/bladder	0.767	0.762	0.624	0.857	17
Endocrine-exocrine	0.783	0.778	0.663	0.849	84
Total	0.788	0.782	0.624	0.954	936

Table S2. CAI and Codon Usage

(B) Codon usage in predicted ORFs of H-Inv proteins.

Total tri-nucleotide frequencies (forward strand) for the sequences of each species are shown. Nonredundant proteome datasets for nonhuman species were obtained from the following sites: fly (*Drosophila melanogaster* ; http://flybase.bio.indiana.edu/), worm (*Caenorhabditis elegans* ; http://www.wormbase.org/), budding yeast (*Saccharomyces cerevisiae* ; http://www.pasteur.fr/externe), fission yeast (*Schizosaccharomyces pombe* ; http://www.sanger.ac.uk/), plant (*Arabidopsis thaliana* ; http://mips.gsf.de/proj/thal/index.html), and bacteria (*Escherichia coli* K12; ftp://ftp.ncbi.nih.gov/genbank/genomes/Bacteria/Escherichia_coli_K12/).

Total tri-nucleotide frequencies (forward strand) for the sequences of each specie.

	All H-Inv proteins	52.4	H-Inv I-IV proteins	52.3	Budding yeast	39.6	Fission yeast	39.6	Worm	42.8	Bacteria	51.8	Plant	44.1	Fly	53.8
	**frequency**	%	**frequency**	**%**	frequency	**%**	**frequency**	%	**frequency**	**%**	frequency	**%**	**frequency**	%	**frequency**	**%**
ttt	252,900	1.37891	319,595	1.49287	254,287	2.83314	246,228	3.50041	690,695	2.46172	75,058	1.83909	754,094	2.18091	323,198	1.14554
ttc	279,754	1.52533	333,100	1.55595	174,054	1.93923	162,147	2.30510	668,555	2.38281	58,440	1.43191	742,906	2.14855	424,079	1.50310
tta	146,954	0.80125	176,396	0.82397	180,840	2.01483	160,242	2.27802	330,589	1.17826	61,634	1.51017	480,724	1.39030	191,909	0.68020
ttg	248,996	1.35762	293,214	1.36964	190,564	2.12317	174,973	2.48744	615,918	2.19520	68,800	1.68575	810,654	2.34448	368,023	1.30441
ctt	279,975	1.52653	333,128	1.55608	146,060	1.62733	159,093	2.26168	440,100	1.56857	44,422	1.08844	721,202	2.08578	338,327	1.19916
ctc	320,451	1.74722	389,334	1.81862	146,060	1.62733	93,003	1.32214	420,827	1.49988	37,300	0.91393	591,562	1.71085	394,391	1.39787
cta	165,400	0.90183	192,448	0.89895	125,575	1.39910	94,847	1.34836	261,329	0.93141	27,833	0.68197	398,669	1.15299	266,856	0.94584
ctg	516,409	2.81566	601,124	2.80792	117,220	1.30601	99,257	1.41105	407,190	1.45127	113,319	2.77657	518,425	1.49933	610,805	2.16492
att	216,682	1.18143	256,804	1.19956	250,265	2.78833	202,408	2.87746	730,683	2.60424	74,581	1.82740	634,968	1.83638	347,647	1.23219
atc	242,361	1.32145	279,011	1.30329	154,714	1.72375	118,201	1.68036	565,319	2.01486	69,666	1.70697	619,584	1.79189	524,688	1.85969
ata	146,936	0.80115	172,576	0.80612	182,395	2.03216	117,786	1.67446	371,272	1.32326	40,028	0.98078	438,863	1.26923	246,268	0.87287
atg	340,663	1.85743	391,498	1.82873	188,228	2.09715	152,483	2.16772	638,611	2.27608	79,746	1.95396	779,807	2.25527	498,988	1.76860
gtt	178,746	0.97459	212,280	0.99158	149,777	1.66874	135,861	1.93142	444,283	1.58348	69,741	1.70881	678,115	1.96117	299,365	1.06106
gtc	199,746	1.08909	235,634	1.10067	83,712	0.93268	73,300	1.04204	314,935	1.12247	48,804	1.19581	394,085	1.13973	351,592	1.24617
gta	121,917	0.66474	143,021	0.66807	103,579	1.15403	79,782	1.13419	229,535	0.81809	44,195	1.08288	325,055	0.94009	188,112	0.66674
gtg	334,510	1.82388	387,873	1.8118	95,023	1.05870	73,678	1.04742	355,515	1.26710	70,408	1.72515	502,956	1.45459	475,590	1.68567
tct	283,647	1.54655	343,475	1.60441	140,325	1.56343	133,465	1.89735	488,462	1.74094	51,552	1.26314	768,134	2.22151	349,698	1.23946
tcc	320,039	1.74498	382,918	1.78866	112,584	1.25436	98,405	1.39894	394,070	1.40451	42,500	1.04135	486,742	1.40770	476,928	1.69041
tca	326,069	1.77786	383,194	1.78994	177,855	1.98158	134,779	1.91603	668,610	2.38300	58,888	1.44289	722,915	2.09074	442,826	1.56954
tcg	112,756	0.61479	127,744	0.59671	74,946	0.83501	80,000	1.13729	418,493	1.49156	61,270	1.50125	370,345	1.07107	423,860	1.50232
cct	394,194	2.1493	467,812	2.1852	91,838	1.02321	89,830	1.27703	216,203	0.77057	44,044	1.07918	399,422	1.15516	393,861	1.39599
ccc	379,519	2.06929	451,139	2.10732	61,171	0.68154	50,367	0.71602	165,833	0.59105	34,098	0.83548	200,487	0.57983	369,717	1.31042
cca	437,419	2.38498	513,546	2.39883	148,100	1.65006	88,427	1.25709	466,527	1.66276	54,372	1.33224	474,036	1.37095	688,949	2.44190
ccg	194,944	1.06291	223,062	1.04195	55,015	0.61295	47,118	0.66983	272,227	0.97025	82,049	2.01038	277,914	0.80375	470,445	1.66743
act	244,657	1.33397	286,604	1.33876	143,608	1.60001	112,219	1.59532	435,211	1.55114	46,731	1.14501	491,521	1.42152	366,726	1.29982
acc	305,753	1.66708	353,337	1.65048	108,227	1.20581	72,513	1.03085	297,857	1.06160	62,735	1.53715	387,048	1.11938	443,846	1.57316
aca	328,721	1.79232	383,382	1.79082	179,555	2.00052	110,817	1.57539	532,090	1.89643	47,993	1.17594	593,575	1.71667	484,815	1.71837
acg	126,996	0.69243	143,226	0.66903	90,539	1.00874	63,970	0.90940	319,547	1.13890	63,839	1.56420	307,842	0.89031	417,996	1.48154
gct	359,425	1.95973	417,726	1.95125	106,925	1.19131	110,686	1.57353	389,570	1.38847	80,546	1.97356	570,781	1.65075	500,744	1.77483
gcc	400,463	2.18348	467,782	2.18506	74,308	0.82790	54,457	0.77417	263,030	0.93747	75,230	1.84330	277,581	0.80279	633,567	2.24560
gca	356,479	1.94366	413,668	1.93229	117,085	1.30450	88,463	1.25760	392,083	1.39743	84,489	2.07017	462,267	1.33692	669,420	2.37268
gcg	166,912	0.91007	190,130	0.88812	47,255	0.52649	40,925	0.58179	181,443	0.64668	111,848	2.74053	220,429	0.63750	488,760	1.73235
tat	163,975	0.89406	193,190	0.90241	187,637	2.09056	152,398	2.16651	450,008	1.60388	62,158	1.52301	494,645	1.43056	253,391	0.89811
tac	177,850	0.96971	204,551	0.95548	137,773	1.53500	108,141	1.53735	310,641	1.10716	47,464	1.16297	390,845	1.13036	307,843	1.09111
taa	138,410	0.75467	164,195	0.76697	171,311	1.90866	123,267	1.75238	283,959	1.01206	47,914	1.17400	426,845	1.23447	206,604	0.73228
tag	101,057	0.551	122,601	0.57268	95,046	1.05896	68,851	0.97879	148,119	0.52791	16,154	0.39581	330,977	0.95722	124,657	0.44183
cat	280,215	1.52784	325,791	1.52181	148,190	1.65106	111,500	1.58510	460,242	1.64036	54,530	1.33611	536,614	1.55194	450,100	1.59532
cac	299,047	1.63052	355,533	1.66074	100,098	1.11524	61,473	0.87391	382,479	1.36320	49,452	1.21168	384,347	1.11157	492,562	1.74583
caa	343,848	1.87479	397,142	1.8551	247,428	2.75672	168,646	2.39749	801,827	2.85780	65,016	1.59304	806,214	2.33164	682,554	2.41923
cag	526,027	2.8681	615,909	2.87698	126,510	1.40951	80,867	1.14961	414,760	1.47825	76,744	1.88040	525,618	1.52013	660,032	2.33940
aat	233,534	1.27332	272,064	1.27084	254,783	2.83867	187,873	2.67082	758,451	2.70321	60,156	1.47396	626,032	1.81054	394,100	1.39684
aac	249,657	1.36123	287,506	1.34297	179,034	1.99471	120,925	1.71908	533,534	1.90158	75,172	1.84188	589,386	1.70456	481,225	1.70564
aaa	395,987	2.15908	464,032	2.16755	372,463	4.14980	275,759	3.92022	1,019,223	3.63263	102,910	2.52153	906,492	2.62166	479,384	1.69912
aag	428,835	2.33818	490,034	2.28901	244,694	2.72626	166,182	2.36246	657,942	2.34498	63,756	1.56217	968,132	2.79992	560,029	1.98495
gat	261,154	1.42391	296,924	1.38697	179,306	1.99774	134,170	1.90738	616,059	2.19571	83,632	2.04917	788,652	2.28085	503,782	1.78559
gac	279,312	1.52292	318,632	1.48837	105,530	1.17576	68,980	0.98063	358,051	1.27614	49,210	1.20576	415,408	1.20140	433,758	1.53740
gaa	433,140	2.36165	493,058	2.30313	260,793	2.90563	185,963	2.64367	874,491	3.11679	88,860	2.17727	960,223	2.77705	547,797	1.94160
gag	431,410	2.35222	496,848	2.32084	115,998	1.29239	84,743	1.20472	475,371	1.69428	35,533	0.87064	758,843	2.19464	590,803	2.09403
tgt	272,240	1.48436	322,914	1.50837	123,309	1.37385	111,779	1.58906	448,962	1.60015	51,018	1.25006	587,261	1.69841	323,338	1.14603
tgc	351,712	1.91767	410,929	1.9195	112,607	1.25461	107,695	1.53100	419,823	1.49630	88,599	2.17087	483,465	1.39822	581,787	2.06207
tga	365,365	1.99211	416,281	1.9445	201,979	2.25035	155,824	2.21521	621,065	2.21355	88,021	2.15671	802,551	2.32105	408,902	1.44930
tgg	451,416	2.4613	523,801	2.44674	152,733	1.70168	125,093	1.77834	527,384	1.87966	104,635	2.56379	738,564	2.13599	638,004	2.26133
cgt	104,255	0.56844	118,973	0.55574	67,824	0.75566	71,705	1.01937	277,201	0.98798	69,128	1.69379	288,716	0.83499	291,393	1.03281
cgc	164,371	0.89621	188,497	0.88049	50,585	0.56359	42,046	0.59773	197,773	0.70489	102,245	2.50523	195,657	0.56586	498,354	1.76635
cga	137,149	0.74779	153,836	0.71859	96,028	1.06990	78,470	1.11554	463,845	1.65320	69,770	1.70952	393,846	1.13904	542,457	1.92267
cgg	196,143	1.06945	223,217	1.04267	53,191	0.59263	39,792	0.56569	252,894	0.90134	77,863	1.90782	298,311	0.86274	468,136	1.65925
agt	247,929	1.35181	289,007	1.34999	123,999	1.38154	95,639	1.35962	385,052	1.37237	39,143	0.95909	511,741	1.48000	358,778	1.27164
agc	396,576	2.16229	458,017	2.13945	106,451	1.18603	91,724	1.30396	384,161	1.36919	66,249	1.62325	528,238	1.52771	639,913	2.26809
aga	463,814	2.5289	532,417	2.48698	231,546	2.57977	135,265	1.92294	634,472	2.26133	44,793	1.09753	998,140	2.88671	454,900	1.61234
agg	376,542	2.05305	442,113	2.06516	119,850	1.33531	76,970	1.09421	288,770	1.02921	41,676	1.02116	539,682	1.56081	479,960	1.70116
ggt	210,214	1.14617	247,518	1.15619	117,257	1.30642	83,498	1.18702	233,053	0.83063	73,246	1.79469	512,491	1.48217	341,829	1.21157
ggc	370,026	2.01753	431,156	2.01398	76,070	0.84754	53,066	0.75439	224,371	0.79968	95,020	2.32820	323,700	0.93617	573,399	2.03234
gga	444,575	2.424	511,315	2.38841	132,239	1.47334	105,292	1.49684	611,622	2.17989	55,908	1.36987	740,367	2.14121	670,354	2.37599
ggg	314,319	1.71379	374,069	1.74732	64,051	0.71363	40,944	0.58206	155,174	0.55306	49,125	1.20367	322,341	0.93224	297,344	1.05390
