# Supplementary material for: Integrative Annotation of 21,037 Human Genes Validated by Full-Length cDNA Clones
Source: PLoS Biol. 2004 Apr 20;2(6):e162. doi: 10.1371/journal.pbio.0020162 (PMC393292; doi:10.1371/journal.pbio.0020162)

Table S3. Tissue Library Origins of H-Inv Proteins

The results of classification into five similarity categories for each of ten tissue classes.

(A) Numbers of H-Inv proteins.

Tissue library origin	Category I protein	Category II protein	Category III protein	Category IV protein	Category V protein	total
Neural	1,076	1,061	724	413	1,390	4,664
Blood/spleen/LND	476	348	203	128	501	1,656
Dermal_connective	528	294	176	171	336	1,505
Placenta/testis/ovary	1,016	847	521	372	1,266	4,022
Muscle/heart	212	143	79	42	158	634
Stomach/colon	250	221	124	96	427	1,118
Liver	108	103	70	42	116	439
Lung	503	192	110	92	267	1,164
Kidney/bladder	257	147	71	46	158	679
Endocrine_exocrine	297	245	113	81	575	1,311
total	5,069	4,103	2,531	1,705	6,159	19,567

(B) Histogram.

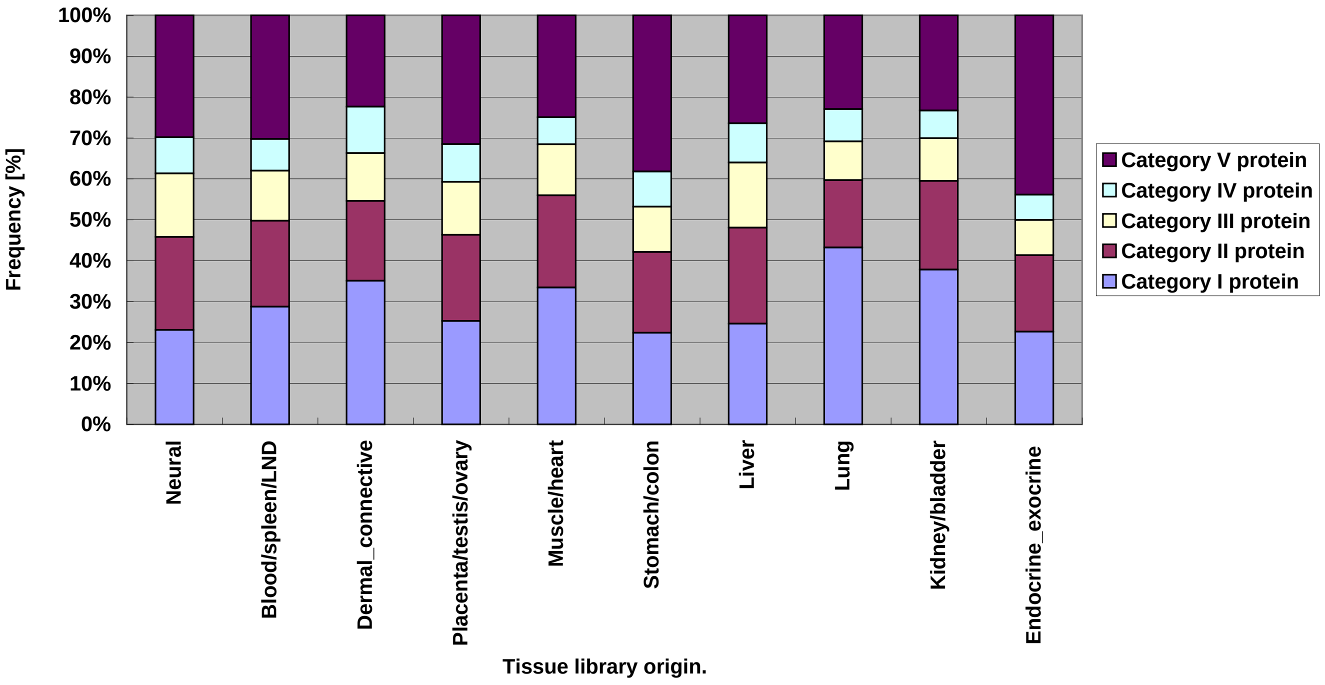

Supplement: Table S3 — The results of classification into five similarity categories for each of ten tissue classes. (A) Numbers of H-Inv proteins. (B) Histogram. (10 KB PDF). [file pbio.0020162.st003.pdf]
