# Supplementary material for: Integrative Annotation of 21,037 Human Genes Validated by Full-Length cDNA Clones
Source: PLoS Biol. 2004 Apr 20;2(6):e162. doi: 10.1371/journal.pbio.0020162 (PMC393292; doi:10.1371/journal.pbio.0020162)
Supplement: Table S4 — The top 40 InterPro IDs identified in H-Inv proteins and proteins from other species are listed for all types (A) and for each type of family, domain, and repeat (B–D). Analyses were conducted by InterPro ver. 3.1. Nonredundant proteome datasets of other species were obtained from the following sites: fly (Drosophila melanogaster; http://flybase.bio.indiana.edu/), worm (Caenorhabditis elegans; http://www.wormbase.org/), budding yeast (Saccharomyces cerevisiae; http://www.pasteur.fr/externe), fission yeast (Schizosaccharomyces pombe; http://www.sanger.ac.uk/), plant (Arabidopsis thaliana; http://mips.gsf.de/proj/thal/index.html), and bacteria (Escherichia coli K12; ftp://ftp.ncbi.nih.gov/genbank/genomes/Bacteria/Escherichia_coli_K12/). (36 KB PDF). [file pbio.0020162.st004.pdf]

Table S4. The InterPro IDs Identified in H-Inv Proteins

The top 40 InterPro IDs identified in H-Inv proteins and proteins from other species are listed for all types (A) and for each type of family, domain, and repeat (B–D). Analyses were conducted by InterPro ver. 3.1. Nonredundant proteome datasets of other species were obtained from the following sites: fly (*Drosophila melanogaster* ; http://flybase.bio.indiana.edu/), worm (*Caenorhabditis elegans* ; http://www.wormbase.org/), budding yeast (*Saccharomyces cerevisiae* ; http://www.pasteur.fr/externe), fission yeast (*Schizosaccharomyces pombe* ; http://www.sanger.ac.uk/), plant (*Arabidopsis thaliana* ; http://mips.gsf.de/proj/thal/index.html), and bacteria (*Escherichia coli* K12; ftp://ftp.ncbi.nih.gov/genbank/genomes/Bacteria/Escherichia_coli_K12/).

(A) The top 40 InterPro IDs identified in H-Inv proteins.

	Human		Fly		Worm		Budding Yeast		Fission Yeast		Plant		Bacteria			
InterPro ID	No. of proteins	Rank	No. of proteins	Rank	No. of proteins	Rank	No. of proteins	Rank	No. of proteins	Rank	No. of proteins	Rank	No. of proteins	Rank	Name	Type
IPR000822	549	1	393	2	184	13	47	13	25	15	139	21	0	NA	Zn-finger, C2H2 type	Domain
IPR000719	271	2	379	3	544	2	118	1	110	2	1040	1	1	710	Eukaryotic protein kinase	Family
IPR002290	251	3	359	4	516	3	114	2	107	3	1008	2	0	NA	Serine/Threonine protein kinase	Family
IPR001245	231	4	335	5	419	5	105	3	98	4	985	3	0	NA	Tyrosine protein kinase	Family
IPR003599	229	5	148	12	82	41	0	NA	0	NA	0	NA	0	NA	Immunoglobulin subtype	Domain
IPR001680	223	6	223	9	151	17	98	4	116	1	236	11	0	NA	G-protein beta WD-40 repeat	Repeat
IPR003006	220	7	146	13	83	40	0	NA	0	NA	0	NA	0	NA	Immunoglobulin/major histocompatibility complex	Domain
IPR001909	218	8	0	NA	0	NA	0	NA	0	NA	0	NA	0	NA	KRAB box	Family
IPR001841	192	9	142	14	153	15	33	17	44	9	432	6	0	NA	Zn-finger, RING	Domain
IPR001849	189	10	105	24	90	37	28	25	22	20	27	180	0	NA	Pleckstrin-like	Domain
IPR000504	162	11	246	7	139	21	54	9	72	5	263	8	0	NA	RNA-binding region RNP-1 (RNA recognition motif)	Domain
IPR002110	158	12	133	19	113	23	18	40	15	44	115	30	4	199	Ankyrin	Repeat
IPR002048	154	13	135	17	94	35	14	60	18	33	166	13	0	NA	Calcium-binding EF-hand	Domain
IPR001452	134	14	117	21	82	41	24	30	21	22	6	649	0	NA	SH3 domain	Domain
IPR003598	128	15	139	16	80	45	0	NA	0	NA	0	NA	0	NA	Immunoglobulin C-2 type	Domain
IPR003579	121	16	103	27	78	48	30	23	21	22	95	41	0	NA	Ras small GTPase, Rab type	Family
IPR000210	117	17	141	15	153	15	3	355	3	308	70	73	0	NA	BTB/POZ domain	Domain
IPR001440	112	18	106	23	64	59	33	17	38	11	146	18	12	55	TPR repeat	Repeat
IPR000561	112	18	113	22	144	19	2	501	0	NA	11	419	0	NA	EGF-like domain	Domain
IPR001356	109	20	134	18	103	30	9	98	2	450	94	43	0	NA	Homeobox	Domain
IPR003577	105	21	89	35	67	56	26	27	19	30	74	65	0	NA	Ras small GTPase, Ras type	Family
IPR000104	104	22	602	1	71	52	32	21	17	37	72	68	12	55	Antifreeze protein, type I	Family
IPR001478	103	23	97	30	82	41	2	501	1	763	15	315	6	129	PDZ/DHR/GLGF domain	Domain
IPR003591	101	24	104	25	45	86	4	260	6	134	95	41	0	NA	Leucine-rich repeat, typical subtype	Repeat
IPR003578	98	25	82	36	62	63	25	28	18	33	74	65	0	NA	Ras small GTPase, Rho type	Family
IPR001806	97	26	74	39	55	70	23	31	18	33	73	67	0	NA	Ras GTPase superfamily	Family
IPR000276	85	27	97	30	405	6	0	NA	0	NA	0	NA	0	NA	Rhodopsin-like GPCR superfamily	Family
IPR003593	84	28	160	11	118	22	82	6	70	6	307	7	118	1	AAA ATPase	Domain
IPR000008	82	29	62	46	66	58	11	71	13	52	100	38	0	NA	C2 domain	Domain
IPR003961	80	30	77	38	60	64	1	835	1	763	3	957	0	NA	Fibronectin, type III	Domain
IPR001650	79	31	101	28	94	35	76	8	66	8	146	18	18	30	Helicase, C-terminal	Domain
IPR001410	79	31	99	29	99	31	78	7	67	7	148	17	21	23	DEAD/DEAH box helicase	Domain
IPR000980	69	33	57	50	74	50	1	835	1	763	2	1172	0	NA	SH2 motif	Domain
IPR000379	68	34	165	10	140	20	37	14	25	15	221	12	22	20	Esterase/lipase/thioesterase, active site	Family
IPR002041	62	35	52	54	36	113	16	48	13	52	60	90	0	NA	GTP-binding nuclear protein Ran	Family
IPR001965	62	35	54	52	41	96	15	53	19	30	86	49	0	NA	Zn-finger-like, PHD finger	Domain
IPR001798	61	37	25	149	18	208	6	165	4	211	121	28	1	710	Kelch repeat	Repeat
IPR001092	60	38	67	42	45	86	8	111	4	211	128	24	0	NA	Basic helix-loop-helix dimerization domain bHLH	Domain
IPR000047	60	38	82	36	50	79	6	165	1	763	61	87	0	NA	Helix-turn-helix motif, lambda-like repressor	Domain
IPR001781	59	40	70	40	40	99	4	260	4	211	14	345	0	NA	Zn-binding protein, LIM	Domain

NA indicates not applicable.

Table S4. The InterPro IDs Identified in H-Inv Proteins

(B) The top 40 InterPro families identified in H-Inv proteins.

	Human		Fly		Worm		Budding yeast		Fission yeast		Plant		Bacteria		
InterPro ID	No. of proteins	Rank	No. of proteins	Rank	No. of proteins	Rank	No. of proteins	Rank	No. of proteins	Rank	No. of proteins	Rank	No. of proteins	Rank	Name
IPR000719	271	1	379	3	544	2	118	1	110	2	1040	1	1	710	Eukaryotic protein kinase
IPR002290	251	2	359	4	516	3	114	2	107	3	1008	2	0	NA	Serine/Threonine protein kinase
IPR001245	231	3	335	5	419	5	105	3	98	4	985	3	0	NA	Tyrosine protein kinase
IPR001909	218	4	0	NA	0	NA	0	NA	0	NA	0	NA	0	NA	KRAB box
IPR003579	121	5	103	27	78	48	30	23	21	22	95	41	0	NA	Ras small GTPase, Rab type
IPR003577	105	6	89	35	67	56	26	27	19	30	74	65	0	NA	Ras small GTPase, Ras type
IPR000104	104	7	602	1	71	52	32	21	17	37	72	68	12	55	Antifreeze protein, type I
IPR003578	98	8	82	36	62	63	25	28	18	33	74	65	0	NA	Ras small GTPase, Rho type
IPR001806	97	9	74	39	55	70	23	31	18	33	73	67	0	NA	Ras GTPase superfamily
IPR000276	85	10	97	30	405	6	0	NA	0	NA	0	NA	0	NA	Rhodopsin-like GPCR superfamily
IPR000379	68	11	165	10	140	20	37	14	25	15	221	12	22	20	Esterase/lipase/thioesterase, active site
IPR002041	62	12	52	54	36	113	16	48	13	52	60	90	0	NA	GTP-binding nuclear protein Ran
IPR001254	52	13	247	6	11	314	0	NA	1	763	13	365	4	199	Serine protease, trypsin family
IPR000387	51	14	59	49	110	25	10	80	8	91	18	254	1	710	Tyrosine specific protein phosphatase and dual specificity protein phosphatase
IPR001314	47	15	236	8	9	377	0	NA	0	NA	8	537	2	411	Chymotrypsin serine protease, family S1
IPR001993	45	16	69	41	38	106	34	16	23	19	60	90	0	NA	Mitochondrial substrate carrier
IPR001394	45	16	32	106	33	122	18	40	16	41	32	158	0	NA	Ubiquitin thiolesterase, family 2
IPR002198	40	18	62	46	79	46	13	64	20	27	90	47	18	30	Short-chain dehydrogenase/reductase SDR
IPR003959	36	19	50	57	40	99	33	17	31	12	110	31	9	78	AAA ATPase, central region
IPR002347	35	20	47	62	58	67	9	98	12	62	70	73	16	36	Glucose/ribitol dehydrogenase
IPR000251	35	20	34	95	20	190	8	111	4	211	42	118	0	NA	ADP-ribosylation factor family
IPR000910	34	22	33	101	18	208	7	132	8	91	16	294	0	NA	HMG1/2 (high mobility group) box
IPR005821	33	23	63	44	109	26	3	355	1	763	30	165	1	710	Ion transport protein
IPR001664	33	23	3	880	15	248	0	NA	0	NA	0	NA	0	NA	Intermediate filament protein
IPR005828	29	25	131	20	88	38	50	11	39	10	94	43	47	4	General substrate transporter
IPR000301	28	26	51	56	21	183	0	NA	0	NA	7	587	0	NA	CD9/CD37/CD63 antigen
IPR005834	27	27	50	57	43	90	27	26	22	20	72	68	22	20	haloacid dehalogenase-like hydrolase
IPR001164	27	27	15	238	9	377	6	165	6	134	17	272	0	NA	Human Rev interacting-like protein (hRIP)
IPR000832	27	27	32	106	12	296	0	NA	0	NA	1	1476	0	NA	G-protein coupled receptors family 2 (secretin-like)
IPR004000	26	30	17	217	13	278	10	80	10	71	18	254	0	NA	Actin/actin-like
IPR001878	26	30	21	171	33	122	11	71	7	109	158	14	0	NA	Zn-finger, CCHC type
IPR001723	25	32	36	90	34	117	0	NA	0	NA	0	NA	0	NA	Steroid hormone receptor
IPR003008	24	33	14	265	17	225	4	260	4	211	20	234	1	710	Tubulin/FtsZ protein
IPR002190	24	33	0	NA	0	NA	0	NA	0	NA	0	NA	0	NA	MAGE protein
IPR002046	24	33	24	155	13	278	7	132	4	211	25	191	0	NA	GTP-binding protein SAR1
IPR005820	23	36	28	122	43	90	1	835	1	763	8	537	0	NA	Cation channel, non-ligand gated
IPR002286	23	36	2	1080	0	NA	0	NA	0	NA	0	NA	0	NA	P2 purinoceptor
IPR001128	23	36	92	34	79	46	3	355	2	450	238	10	0	NA	Cytochrome P450
IPR004031	22	39	1	1405	0	NA	0	NA	0	NA	0	NA	0	NA	PMP-22/EMP/MP20 and claudin family
IPR001811	22	39	0	NA	0	NA	0	NA	0	NA	0	NA	0	NA	Small chemokine, interleukin-8 like

NA indicates not applicable.

Table S4. The InterPro IDs Identified in H-Inv Proteins

(C) The top 40 InterPro domains found in H-Inv proteins.

	Human		Fly		Worm		Budding yeast		Fission yeast		Plant		Bacteria		
InterPro ID	No. of proteins	Rank	No. of proteins	Rank	No. of proteins	Rank	No. of proteins	Rank	No. of proteins	Rank	No. of proteins	Rank	No. of proteins	Rank	Name
IPR000822	549	1	393	2	184	13	47	13	25	15	139	21	0	NA	Zn-finger, C2H2 type
IPR003599	229	2	148	12	82	41	0	NA	0	NA	0	NA	0	NA	Immunoglobulin subtype
IPR003006	220	3	146	13	83	40	0	NA	0	NA	0	NA	0	NA	Immunoglobulin/major histocompatibility complex
IPR001841	192	4	142	14	153	15	33	17	44	9	432	6	0	NA	Zn-finger, RING
IPR001849	189	5	105	24	90	37	28	25	22	20	27	180	0	NA	Pleckstrin-like
IPR000504	162	6	246	7	139	21	54	9	72	5	263	8	0	NA	RNA-binding region RNP-1 (RNA recognition motif)
IPR002048	154	7	135	17	94	35	14	60	18	33	166	13	0	NA	Calcium-binding EF-hand
IPR001452	134	8	117	21	82	41	24	30	21	22	6	649	0	NA	SH3 domain
IPR003598	128	9	139	16	80	45	0	NA	0	NA	0	NA	0	NA	Immunoglobulin C-2 type
IPR000210	117	10	141	15	153	15	3	355	3	308	70	73	0	NA	BTB/POZ domain
IPR000561	112	11	113	22	144	19	2	501	0	NA	11	419	0	NA	EGF-like domain
IPR001356	109	12	134	18	103	30	9	98	2	450	94	43	0	NA	Homeobox
IPR001478	103	13	97	30	82	41	2	501	1	763	15	315	6	129	PDZ/DHR/GLGF domain
IPR003593	84	14	160	11	118	22	82	6	70	6	307	7	118	1	AAA ATPase
IPR000008	82	15	62	46	66	58	11	71	13	52	100	38	0	NA	C2 domain
IPR003961	80	16	77	38	60	64	1	835	1	763	3	957	0	NA	Fibronectin, type III
IPR001650	79	17	101	28	94	35	76	8	66	8	146	18	18	30	Helicase, C-terminal
IPR001410	79	17	99	29	99	31	78	7	67	7	148	17	21	23	DEAD/DEAH box helicase
IPR000980	69	19	57	50	74	50	1	835	1	763	2	1172	0	NA	SH2 motif
IPR001965	62	20	54	52	41	96	15	53	19	30	86	49	0	NA	Zn-finger-like, PHD finger
IPR001092	60	21	67	42	45	86	8	111	4	211	128	24	0	NA	Basic helix-loop-helix dimerization domain bHLH
IPR000047	60	21	82	36	50	79	6	165	1	763	61	87	0	NA	Helix-turn-helix motif, lambda-like repressor
IPR001781	59	23	70	40	40	99	4	260	4	211	14	345	0	NA	Zn-binding protein, LIM
IPR000637	58	24	53	53	31	134	7	132	6	134	63	83	1	710	HMG-I and HMG-Y DNA-binding domain (A+T-hook)
IPR001881	56	25	45	70	40	99	0	NA	0	NA	17	272	0	NA	EGF-like calcium-binding
IPR001660	56	25	40	81	23	170	4	260	4	211	9	486	0	NA	Sterile alpha motif SAM
IPR003877	55	27	25	149	14	256	4	260	3	308	7	587	0	NA	SPla/RYanodine receptor SPRY
IPR003596	55	27	13	281	3	786	0	NA	0	NA	0	NA	0	NA	Immunoglobulin V-type
IPR000483	53	29	35	91	9	377	0	NA	0	NA	0	NA	0	NA	Cysteine-rich flanking region, C-terminal
IPR000198	51	30	24	155	25	156	11	71	10	71	10	443	0	NA	RhoGAP domain
IPR000219	50	31	46	64	27	149	6	165	8	91	0	NA	0	NA	Dbl domain (dbl/cdc24 rhoGEF family)
IPR001810	49	32	46	64	433	4	14	60	13	52	650	4	0	NA	Cyclin-like F-box
IPR001870	48	33	13	281	2	968	0	NA	0	NA	0	NA	0	NA	Gamma-carboxylase-like
IPR004822	47	34	30	112	88	38	17	45	20	27	77	59	0	NA	Histone-fold/TFIID-TAF/NF-Y domain
IPR000315	47	34	11	329	20	190	0	NA	0	NA	32	158	0	NA	Zn-finger, B-box
IPR003309	45	36	0	NA	0	NA	0	NA	0	NA	0	NA	0	NA	Transcriptional regulator SCAN
IPR000048	45	36	60	48	24	161	4	260	6	134	79	57	0	NA	IQ calmodulin-binding region
IPR000626	44	38	28	122	34	117	13	64	18	33	87	48	0	NA	Ubiquitin domain
IPR000051	43	39	46	64	53	74	21	34	31	12	107	33	29	12	SAM (and some other nucleotide) binding motif
IPR002035	41	40	11	329	55	70	3	355	3	308	23	207	4	199	Von Willebrand factor, type A

NA indicates not applicable.

Table S4. The InterPro IDs Identified in H-Inv Proteins

(D) The top 40 InterPro repeats found in H-Inv proteins.

	Human		Fly		Worm		Budding yeast		Fission yeast		Plant		Bacteria		
InterPro ID	No. of proteins	Rank	No. of proteins	Rank	No. of proteins	Rank	No. of proteins	Rank	No. of proteins	Rank	No. of proteins	Rank	No. of proteins	Rank	Name
IPR001680	223	1	223	9	151	17	98	4	116	1	236	11	0	NA	G-protein beta WD-40 repeat
IPR002110	158	2	133	19	113	23	18	40	15	44	115	30	4	199	Ankyrin
IPR001440	112	3	106	23	64	59	33	17	38	11	146	18	12	55	TPR repeat
IPR003591	101	4	104	25	45	86	4	260	6	134	95	41	0	NA	Leucine-rich repeat, typical subtype
IPR001798	61	5	25	149	18	208	6	165	4	211	121	28	1	710	Kelch repeat
IPR003962	40	6	42	75	27	149	0	NA	0	NA	0	NA	0	NA	Fibronectin, type III repeat
IPR000087	36	7	27	131	181	14	0	NA	0	NA	8	537	0	NA	Collagen triple helix repeat
IPR000225	34	8	23	163	11	314	2	501	6	134	78	58	0	NA	Armadillo repeat
IPR002017	33	9	37	87	16	237	2	501	1	763	3	957	2	411	Spectrin repeat
IPR000884	33	9	20	178	42	94	0	NA	0	NA	0	NA	0	NA	Thrombospondin, type I
IPR003885	19	11	28	122	10	342	1	835	2	450	17	272	0	NA	Leucine-rich repeat, cysteine-containing type
IPR003590	19	11	18	198	5	586	1	835	1	763	7	587	0	NA	Leucine rich repeat, ribonuclease inhibitor type
IPR002165	15	13	9	398	5	586	0	NA	0	NA	0	NA	0	NA	Plexin
IPR000357	15	13	11	329	14	256	10	80	7	109	17	272	0	NA	HEAT repeat
IPR004092	9	15	5	627	2	968	0	NA	0	NA	0	NA	0	NA	Mbt repeat
IPR003107	8	16	10	362	7	451	8	111	8	91	14	345	0	NA	RNA-processing protein, HAT helix
IPR001610	8	16	8	437	1	1251	0	NA	2	450	7	587	3	276	PAC motif
IPR001258	8	16	11	329	11	314	0	NA	0	NA	6	649	0	NA	NHL repeat
IPR004018	7	19	4	735	1	1251	0	NA	0	NA	0	NA	0	NA	RPEL repeat
IPR003409	7	19	10	362	1	1251	0	NA	0	NA	15	315	0	NA	MORN motif
IPR001298	7	19	12	304	8	415	0	NA	0	NA	2	1172	0	NA	Filamin/ABP280 repeat
IPR002860	6	22	0	NA	0	NA	7	132	1	763	2	1172	0	NA	Glycosyl hydrolase, BNR repeat
IPR000557	6	22	0	NA	6	505	0	NA	0	NA	0	NA	0	NA	Calponin repeat
IPR000033	6	22	8	437	8	415	0	NA	0	NA	1	1476	0	NA	Low-density lipoprotein receptor, YWTD repeat
IPR001451	5	25	7	488	6	505	4	260	5	176	24	199	15	41	Bacterial transferase hexapeptide repeat
IPR000861	5	25	4	735	1	1251	1	835	3	308	0	NA	0	NA	PKN/rhophilin/rhotekin rho-binding repeat
IPR002885	4	27	5	627	1	1251	2	501	4	211	459	5	0	NA	PPR repeat
IPR002151	4	27	1	1405	3	786	0	NA	0	NA	0	NA	0	NA	Kinesin light chain
IPR001370	4	27	6	546	2	968	1	835	1	763	0	NA	0	NA	Baculovirus inhibitor of apoptosis protein repeat (BIR)
IPR004212	3	30	0	NA	0	NA	0	NA	0	NA	0	NA	0	NA	GTF2I-like repeat
IPR004155	3	30	1	1405	1	1251	1	835	1	763	3	957	1	710	PBS lyase HEAT-like repeat
IPR003267	3	30	3	880	5	586	0	NA	0	NA	2	1172	0	NA	Small proline-rich
IPR002088	3	30	3	880	2	968	2	501	2	450	3	957	0	NA	Protein prenyltransferase, alpha subunit
IPR002015	3	30	3	880	4	668	3	355	3	308	5	728	0	NA	Proteasome/cyclosome, regulatory subunit
IPR000547	3	30	3	880	4	668	5	198	3	308	6	649	0	NA	7-Fold repeat in clathrin and VPS proteins
IPR004153	2	36	1	1405	1	1251	0	NA	0	NA	0	NA	0	NA	CXCXC repeat
IPR003822	2	36	4	735	1	1251	1	835	3	308	23	207	0	NA	Paired amphipathic helix
IPR003134	2	36	1	1405	0	NA	0	NA	0	NA	0	NA	0	NA	Hs1/Cortactin
IPR001330	2	36	3	880	4	668	4	260	4	211	18	254	0	NA	Prenyltransferase/squalene oxidase
IPR001101	2	36	4	735	1	1251	0	NA	0	NA	0	NA	0	NA	Plectin repeat

NA indicates not applicable.
