# Supplementary material for: Integrative Annotation of 21,037 Human Genes Validated by Full-Length cDNA Clones
Source: PLoS Biol. 2004 Apr 20;2(6):e162. doi: 10.1371/journal.pbio.0020162 (PMC393292; doi:10.1371/journal.pbio.0020162)

Table S5. GO Term Assignment to H-Inv Proteins

(A) Molecular function.

term	Human	Fly	Worm	Budding yeast	Fission yeast	Plant	Bacteria
	Frequency (%)						
ligand binding or carrier	41.2	35.8	32.1	39.0	39.2	36.6	30.8
enzyme	34.8	39.6	34.3	41.0	42.2	44.7	37.0
transporter	7.9	10.3	9.3	9.2	7.5	7.2	17.4
signal transducer	6.5	5.1	13.0	0.8	0.9	1.6	3.0
transcription regulator	3.5	2.9	5.6	2.5	1.9	2.6	6.1
structural molecule	2.4	1.7	1.8	3.8	4.6	1.9	1.6
enzyme regulator	0.9	1.0	0.6	0.2	0.3	0.8	0.0
motor	0.7	0.7	0.4	0.3	0.4	0.5	0.4
translation regulator	0.5	0.6	0.3	0.8	0.9	0.5	0.6
apoptosis regulator	0.4	0.1	0.0	0.0	0.1	0.0	0.0
cell adhesion molecule	0.2	0.1	0.0	0.0	0.0	0.0	0.0
chaperone	0.2	0.2	0.1	0.3	0.3	0.2	0.5
other terms	1.1	1.8	2.4	2.0	1.8	3.4	2.7

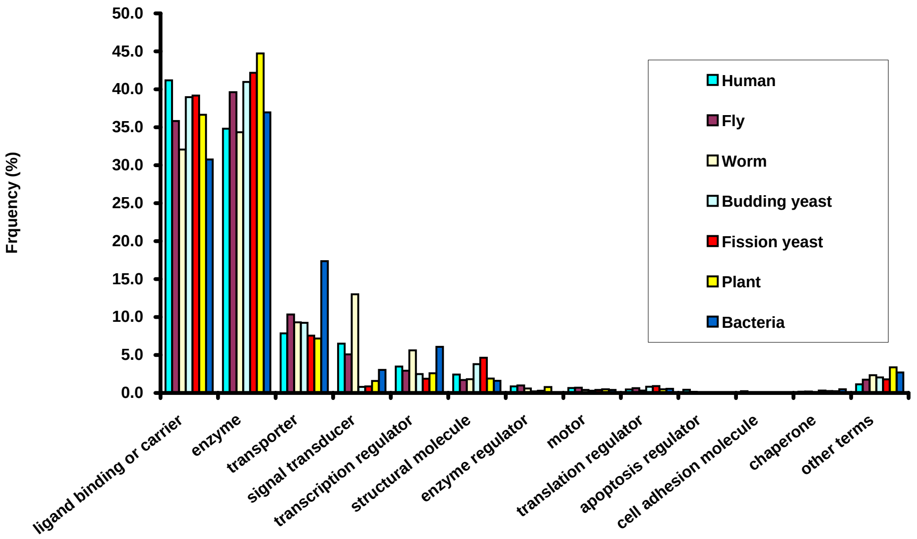

Table S5. GO Term Assignment to H-Inv Proteins

(B) Cellular component.

term	Human	Fly	Worm	Budding yeast	Fission yeast	Plant	Bacteria
	Frequency (%)						
INTRACELLULAR*							
nucleus	29.9	24.9	21.7	21.7	19.6	28.3	0.6
intracellular	14.7	8.3	6.1	13.1	16.2	11.1	15.7
cytoplasm	13.8	15.3	9.4	20.8	22.5	16.3	10.5
ribonucleoprotein complex	4.2	4.3	2.6	10.8	12.6	7.2	4.1
other terms	2.0	1.4	2.4	1.4	1.9	2.4	3.8
MEMBRANE*							
membrane	17.9	25.4	36.0	17.9	14.6	22.9	41.5
integral membrane protein	5.5	6.1	13.2	3.9	2.6	3.1	9.1
plasma membrane	2.5	1.9	1.8	0.5	0.5	0.4	0.6
mitochondrial membrane	1.9	2.6	1.3	3.2	2.8	2.1	1.8
other terms	2.6	3.4	1.6	3.9	3.5	2.6	2.4
EXTRACELLULAR*							
extracellular	2.9	3.3	1.5	0.2	0.1	0.4	0.1
extracellular matrix	0.6	0.4	0.3	0.0	0.1	0.1	0.0
extracellular space	0.0	0.0	0.0	0.0	0.0	0.0	0.0
OTHER COMPONENTS*	1.5	2.6	2.2	2.8	3.0	3.0	9.7

Asterisk indicates term of parental GO ID.

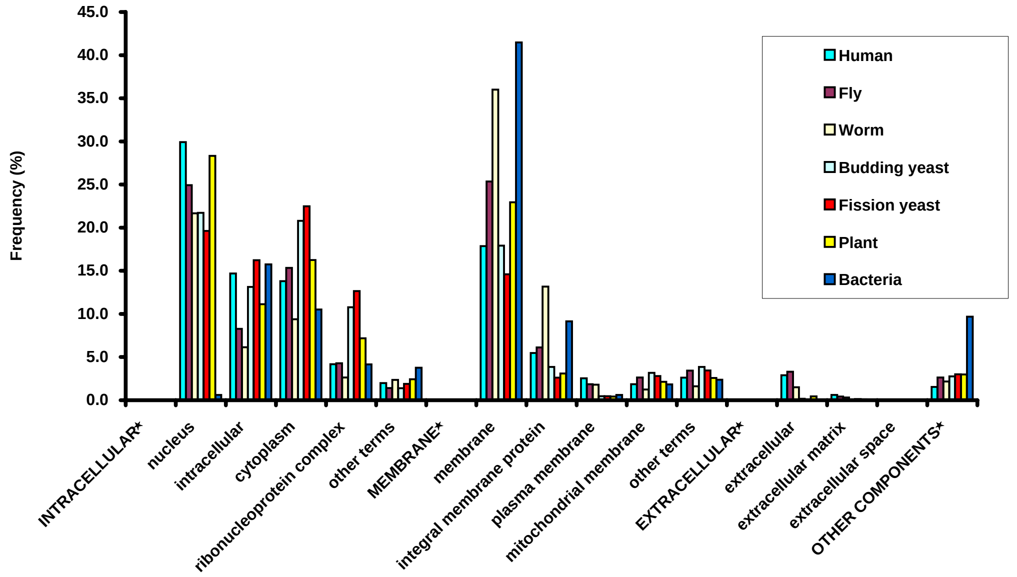

Table S5. GO Term Assignment to H-Inv Proteins

(C) Biological process.

term	Human	Fly	Worm	Budding yeast	Fission yeast	Plant	Bacteria
	Frequency (%)						
CELL COMMUNICATION*							
signal transduction	11.1	8.1	11.1	2.9	3.1	2.6	3.2
other terms	3.4	2.3	1.9	0.6	0.4	2.5	1.4
CELL GROWTH AND/OR MAINTENANCE*							
protein metabolism and modification	18.0	20.3	17.7	19.6	21.4	19.7	8.6
DNA and/or RNA metabolism	15.6	10.1	13.1	14.7	14.1	13.3	18.1
transport	12.9	15.6	14.9	13.1	10.8	10.1	17.5
biosynthesis	6.7	6.6	5.1	14.2	15.5	9.0	11.7
catabolism	6.3	8.8	5.4	4.8	4.9	5.3	4.1
phosphate metabolism	5.5	5.9	8.5	4.5	4.8	9.0	1.3
metabolism	3.6	4.9	4.8	5.0	5.0	4.9	6.2
cell organization and biogenesis	2.5	1.4	2.8	1.4	1.6	1.8	1.1
electron transport	2.5	3.4	2.9	3.1	2.8	4.8	6.0
other terms	9.4	11.1	10.6	16.0	15.0	15.2	20.5
DEVELOPMENTAL PROCESSES*	1.3	1.0	0.8	0.1	0.1	0.2	0.2
PHYSIOLOGICAL PROCESSES*	0.2	0.0	0.2	0.0	0.0	0.1	0.1
OTHER BIOLOGICAL PROCESSES*	0.7	0.3	0.1	0.1	0.3	1.4	0.1

Asterisk indicates term of parental GO ID.

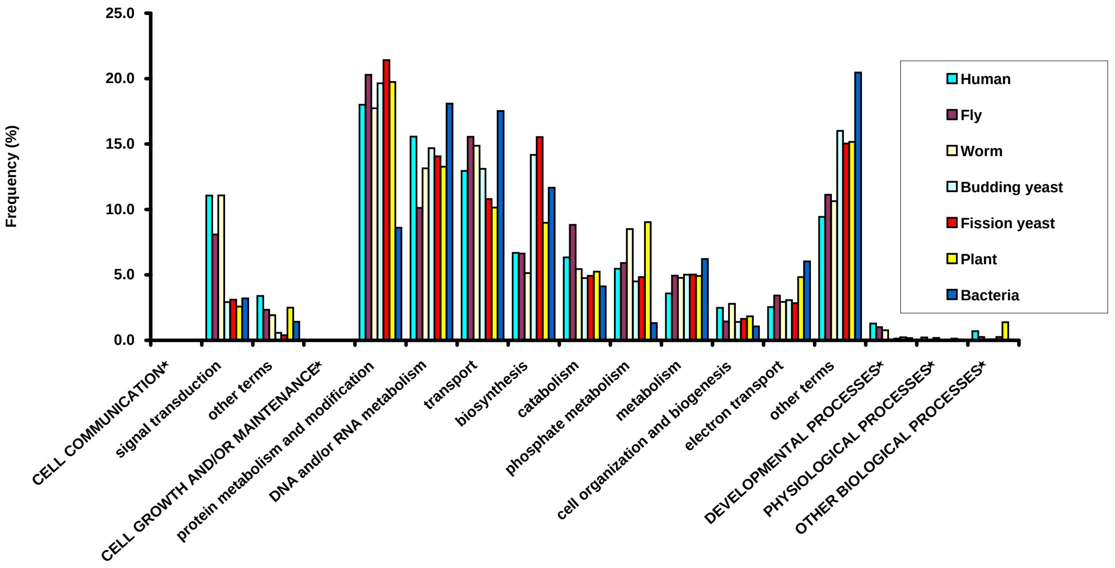

Supplement: Table S5 — (A) Molecular function. (B) Cellular component. (C) Biological process. (74 KB PDF). [file pbio.0020162.st005.pdf]
