# Supplementary material for: Integrative Annotation of 21,037 Human Genes Validated by Full-Length cDNA Clones
Source: PLoS Biol. 2004 Apr 20;2(6):e162. doi: 10.1371/journal.pbio.0020162 (PMC393292; doi:10.1371/journal.pbio.0020162)
Supplement: Table S6 — All these 32 H-Inv proteins were newly assigned enzyme numbers with the support of the KEGG pathway. These enzyme assignments were previously unrepresented in Homo sapiens. (33 KB PDF). [file pbio.0020162.st006.pdf]

Table S6.
List of Newly Assigned Human Enzymes (32 H-Inv Proteins)

All these 32 H-Inv proteins were newly assigned enzyme numbers with the support of the KEGG pathway. These enzyme assignments were previously unrepresented in *Homo sapiens*.

H-Inv ID	H-Inv cluster ID	Acc No.	EC num	KEGG pathway ID	KEGG pathway title
HIT000038588	HIX0001987	BC019263	EC 5.1.3.3	00010	Glycolysis / Gluconeogenesis
HIT000037168	HIX0002249	BC015529	EC 5.3.1.6	00030	Pentose phosphate pathway
HIT000000067	HIX0019361	AB002359	EC 6.3.5.3	00230	Purine metabolism
HIT000004430	HIX0008425	AK001956	EC 4.2.1.70	00240	Pyrimidine metabolism
HIT000012968	HIX0016881	AK056354	EC 2.4.2.9	00240	Pyrimidine metabolism
HIT000018796	HIX0009231	BC030601	EC 4.2.1.70	00240	Pyrimidine metabolism
HIT000034411	HIX0022535	BC009505	EC 4.2.1.70	00240	Pyrimidine metabolism
HIT000005710	HIX0009900	AK022436	EC 6.3.5.1	00251	Glutamate metabolism
HIT000016761	HIX0009707	AK091894	EC 3.5.1.1	00252	Alanine and aspartate metabolism
HIT000014369	HIX0007313	AK057762	EC 1.1.1.103	00260	Glycine, serine and threonine metabolism
HIT000041904	HIX0022766	BC033687	EC 2.1.2.9	00271	Methionine metabolism
HIT000008832	HIX0006550	AK025558	EC 1.1.1.31	00280	Valine, leucine and isoleucine degradation
HIT000004166	HIX0014523	AK001692	EC 3.5.1.18	00300	Lysine biosynthesis
HIT000012380	HIX0021688	AK055766	EC 3.5.1.4	00330	Arginine and proline metabolism
HIT000011231	HIX0010901	AK054617	EC 3.5.2.7	00340	Histidine metabolism
HIT000012476	HIX0022393	AK055862	EC 4.2.1.49	00340	Histidine metabolism
HIT000004593	HIX0002961	AK002119	EC 4.4.1.16	00450	Selenoamino acid metabolism
HIT000024385	HIX0022242	AL096750	EC 3.5.2.9	00480	Glutathione metabolism
HIT000020773	HIX0020869	AK095918	EC 3.2.1.4	00500	Starch and sucrose metabolism
HIT000023644	HIX0000875	AK098814	EC 3.2.1.14	00530	Aminosugars metabolism
HIT000029276	HIX0008220	BC000008	EC 4.1.3.19	00530	Aminosugars metabolism
HIT000003634	HIX0010434	AK001160	EC 3.2.1.14	00530	Aminosugars metabolism
HIT000007189	HIX0009686		E2.7.2.2914		

HIT000014259	HIX0009576	AK057649	EC 4.4.1.14	00640	Propanoate metabolism
HIT000015047	HIX0023118	AK074267	EC 6.2.1.17	00640	Propanoate metabolism
HIT000004284	HIX0000590	AK001810	EC 4.2.1.55	00650	Butanoate metabolism
HIT000008034	HIX0022598	AK024760	EC 6.2.1.16	00650	Butanoate metabolism
HIT000013606	HIX0022188	AK056992	EC 1.1.1.244	00680	Methane metabolism
HIT000016603	HIX0007177	AK091736	EC 2.7.6.2	00730	Thiamine metabolism
HIT000003871	HIX0013930	AK001397	EC 1.4.3.5	00750	Vitamin B6 metabolism
