# Supplementary material for: Integrative Annotation of 21,037 Human Genes Validated by Full-Length cDNA Clones
Source: PLoS Biol. 2004 Apr 20;2(6):e162. doi: 10.1371/journal.pbio.0020162 (PMC393292; doi:10.1371/journal.pbio.0020162)
Supplement: Table S7 — (See also Figure 6.) (9 KB PDF). [file pbio.0020162.st007.pdf]

Table S7.

A Functional Classification of Representative H-Inv cDNAs That Have Homologs in Other Species

Function	Mammal	Animal*	Fungus	Plant	Eukaryote†	Prokaryote
Ligand binding or carrier	3142	2917	1622	1732	1462	715
Enzyme	2361	2298	1665	1719	1468	1241
Transporter	635	597	319	359	273	243
Signal transducer	525	471	63	61	86	31
Transcription regulator	325	291	59	55	35	10
Structural molecule	256	262	190	200	182	117
Enzyme regulator	91	79	20	40	24	26
Motor	75	75	63	64	62	14
Translation regulator	54	53	46	49	36	34
Apoptosis regulator	46	27	3	5	3	2
Cell adhesion molecule	25	24	5	1	5	6
Chaperone	17	17	14	16	13	14
Others	106	100	52	71	35	35

* Non-mammalian animals.

† Eukaryotes other than animals, fungi, and plants.

(See also Figure 6.)
