# Supplementary material for: Integrative Annotation of 21,037 Human Genes Validated by Full-Length cDNA Clones
Source: PLoS Biol. 2004 Apr 20;2(6):e162. doi: 10.1371/journal.pbio.0020162 (PMC393292; doi:10.1371/journal.pbio.0020162)
Supplement: Table S8 — (8 KB PDF). [file pbio.0020162.st008.pdf]

Table S8. Basic Statistics for UTR Sequences Analyzed

	5' UTR				3' UTR		
	Number of sequences analysed	Mean length	Standard deviation of length		Number of sequences analysed	Mean length	Standard deviation of length
Homo sapiens	15,271	349.33	506.29		28,562	925.11	850.65
Pan troglodytes	116	101.34	81.6		75	192.52	130.52
Macaca fascicularis	367	328.91	376.72		379	698.86	634.68
Macaca mulatta	27	71.19	98.36		38	143.74	195.28
Mus musculus	7,368	179.75	266.82		7,914	711.07	672.9
Rattus norvegicus	949	158.08	232.57		1,039	543.67	560.68
