# Supplementary material for: Integrative Annotation of 21,037 Human Genes Validated by Full-Length cDNA Clones
Source: PLoS Biol. 2004 Apr 20;2(6):e162. doi: 10.1371/journal.pbio.0020162 (PMC393292; doi:10.1371/journal.pbio.0020162)
Supplement: Table S9 — One hundred and forty-seven UTR replacements distributed among different species were detected. (9 KB PDF). [file pbio.0020162.st009.pdf]

Table S9. UTR Replacements in Primates and Rodents

One hundred and forty-nine UTR replacements distributed among different species were detected.

	5' UTRs		3' UTRs	
	Number of observed replacements	Number of sequences analyzed	Number of observed replacements	Number of sequences analyzed
Homo sapiens	14	977	9	1,725
Pan troglodytes	2	195	14	95
Macaca	10	158	3	402
Macaca mulatta			3	53
Mus musculus	25	987	29	1,713
Rattus norvegicus	21	744	19	1,289
Total	72	3,061	77	5,277
