# Supplementary material for: Integrative Annotation of 21,037 Human Genes Validated by Full-Length cDNA Clones
Source: PLoS Biol. 2004 Apr 20;2(6):e162. doi: 10.1371/journal.pbio.0020162 (PMC393292; doi:10.1371/journal.pbio.0020162)
Supplement: Table S10 — (31 KB PDF). [file pbio.0020162.st010.pdf]

Table S10. List of the Databases and Software used in the H-Inv Project

	Database/Software	URL	Description
**DNA sequences**			
	DDBJ	http://www.ddbj.nig.ac.jp/Welcome.html	DNA sequence databases, reference data set is available on 15th July 2002
	EMBL	http://www.embl-heidelberg.de/	
	GenBank	http://www.ncbi.nlm.nih.gov/Genbank/index.html	
**Human genome**			
	NCBI	http://www.ncbi.nlm.nih.gov/	Human genome build 34
	UCSC	http://genome.ucsc.edu/	Genome Bioinformatics Site
**Protein sequences**			
	UniProt/SWISS-PROT/TrEMBL/TrEMBL_new	http://www.ebi.ac.uk/swissprot/	non-redundant protein dataset built from SWISS-PROT,TrEMBL, and TrEMBL_new constructed for H-Invitational
	RefSeq	http://www.ncbi.nlm.nih.gov/RefSeq/	A collection aims to provide a comprehensive, integrated, non-redundant set of sequences, including genomic DNA, transcript (RNA), and protein products, for major research organisms.

Domain, motifs, families and post translational modification(PTM).

InterProScan	http://www.ebi.ac.uk/interpro/scan.html	An integrated documentation resource for protein families, domains and sites.

Subcellular localisation

PSORT II	http://psort.nibb.ac.jp/	A computer program for the prediction of protein localization sites in cells.
SOSUI	http://sosui.proteome.bio.tuat.ac.jp/sosuiframe0.html	A computer program for classification and secondary structure prediction of Membrane proteins
HMMER	http://hmmer.wustl.edu/	A computer program for biological sequence analysis using profile hidden markov model
TargetP	http://www.cbs.dtu.dk/services/TargetP/	A program for prediction of the subcellular location of eukaryotic protein sequences

3-D structure

GTOP	http://spock.genes.nig.ac.jp/~genome/gtop.html	A database consisting of data analyses of proteins identified by various genome projects. This database mainly uses sequence homology analyses and features extensive utilization of information on three-dimensional structures.

	3D keynote	In preparation	Prediction of functions out of amino acid sequences based on module structure of proteins.
**Pathway**			
	KEGG	http://kegg.genome.ad.jp/	A suite of databases and associated software, integrating our current knowledge on molecular interaction networks in biological processes
**Disease**			
	OMIM	http://www.ncbi.nlm.nih.gov/omim/	OMIM, Online Mendelian Inheritance in Man, is a database of catalog for human genes and genetic disorders
	GenAtlas	http://www.dsi.univ-paris5.fr/genatlas/	The GENATLAS database compiles the information relevant to the mapping efforts of the Human Genome Project. This information is collected from original articles in the literature or from the proceedings of Human Gene Mapping and Single Chromosome Workshops.

Expression

iAFLP	http://bodymap.ims.u-tokyo.ac.jp/
	http://www.ncbi.nlm.nih.gov/entrez/query.fcgi?cmd=Retrieve&db=PubMed&list_uids=10613853&dopt=Abstract
	http://www.ncbi.nlm.nih.gov/entrez/query.fcgi?cmd=Retrieve&db=PubMed&list_uids=11125076&dopt=Abstract
Long Oligomer DNA-Chip	Watanabe, S. *et al. in Press*
HugeIndex - Affymetrix	http://www.ncbi.nlm.nih.gov/entrez/query.fcgi?cmd=Retrieve&db=PubMed&list_uids=11752297&dopt=Abstract
	http://zlab.bu.edu/HugeIndex/index.htm
TissueDB - Affymetrix	http://www2.genome.rcast.u-tokyo.ac.jp/tp/
Nylon membrane Microarray	http://www.ncbi.nlm.nih.gov/entrez/query.fcgi?cmd=Retrieve&db=PubMed&list_uids=10022985&dopt=Abstract
SAGE	http://www.ncbi.nlm.nih.gov/entrez/query.fcgi?cmd=Retrieve&db=PubMed&list_uids=7570003&dopt=Abstract
	http://www.ncbi.nlm.nih.gov/entrez/query.fcgi?cmd=Retrieve&db=PubMed&list_uids=12119410&dopt=Abstract
dbEST	http://www.ncbi.nlm.nih.gov/entrez/query.fcgi?cmd=Retrieve&db=PubMed&list_uids=8401577&dopt=Abstract
	http://www.ncbi.nlm.nih.gov/dbEST/index.html
BodyMap	http://www.ncbi.nlm.nih.gov/entrez/query.fcgi?cmd=Retrieve&db=PubMed&list_uids=1345164&dopt=Abstract
	http://www.ncbi.nlm.nih.gov/entrez/query.fcgi?cmd=Retrieve&db=PubMed&list_uids=11076866&dopt=Abstract

**Literatures**			
	Gene ontology	http://www.geneontology.org/	A project to produce a dynamic controlled vocabulary that can be applied to all organisms even as knowledge of gene and protein roles in cells is accumulating and changing.
**SNPs**			
	dbSNP	http://www.ncbi.nlm.nih.gov/entrez/query.fcgi?db=Snp	A database for collection of single nucleotide polymorphism
**Sequence analysis**			
	NCBI-BLASTX 2.0.11	http://www.ncbi.nlm.nih.gov/BLAST/	Compare DNA sequence to protein
	NCBI-BLASTN 2.2.2	http://www.ncbi.nlm.nih.gov/BLAST/	Compare DNA sequences
	FASTY 3.4t11	http://fasta.bioch.virginia.edu/fasta/home.html	Compare DNA sequence to protein
	Repeat masker	http://www.genome.washington.edu/UWGC/	Mask repeat and low complexity region in sequence
	GeneMark	http://opal.biology.gatech.edu/GeneMark/	Gene prediction program which has been designed to predict prokaryotic genes
**Protein sequence characterization**			
	InterProScan		
	PSORT II		
	SOSUI		
	HMMER		
	TargetP		
	GTOP		

Sequence alignment

ClustalW	http://crick.genes.nig.ac.jp/homology/clustalw.shtml	A program to make multiple sequence alignment for DNA or proteins
