# Supplementary material for: Integrative Annotation of 21,037 Human Genes Validated by Full-Length cDNA Clones
Source: PLoS Biol. 2004 Apr 20;2(6):e162. doi: 10.1371/journal.pbio.0020162 (PMC393292; doi:10.1371/journal.pbio.0020162)
Supplement: Protocol S1 — (25 KB PDF). [file pbio.0020162.sd004.pdf]

Protocol S1.

A Detailed Functional Annotation Based on Protein Modules

Generally, even state-of-the-art methods can predict the functions of at most 50-60% of all proteins encoded in the complete genomes. During our own analysis, 40.9% of proteins could not be given functional assignment and had to be classified as hypothetical. A novel method, named module 3D-keynotes, was thus developed to extract new amino acid sequence patterns of functional importance from tertiary structures of protein modules (Go et al. 1983; Noguti et al. 1993). Since the module 3D-keynotes define ancient conserved short amino acid patterns in an elaborate manner, it was expected that we could find these patterns for functional prediction even in those proteins previously classified as hypothetical proteins.

The 3D-keynotes have been applied to identify the patterns for assigning information regarding the cellular functions of the ORFs (Yura et al. 1999), and the predicted functions of ORFs in the cyanobacterial genome. These results have been experimentally verified (Yoshihara et al. 2001). Recent extension in the number of patterns allows us to predict the variety of the molecular functions. The application of 196 patterns to amino acid sequences deduced from the H-Inv proteins predicted the function of 350 hypothetical ORFs in which no significant similarity had been detected using more conventional methods (Fig. Protocol S1). The data suggests that there are many novel calcium-binding proteins that share similar modules in the human genome. The existence of a variety of calcium-binding proteins is consistent with current thinking that a calcium ion is employed extensively as a secondary messenger in eukaryotes (Bootman et al. 2001). This indicates a binding response to calcium ion under certain conditions may be conserved. However, the number of gene products involved in this behavior remains to be investigated.

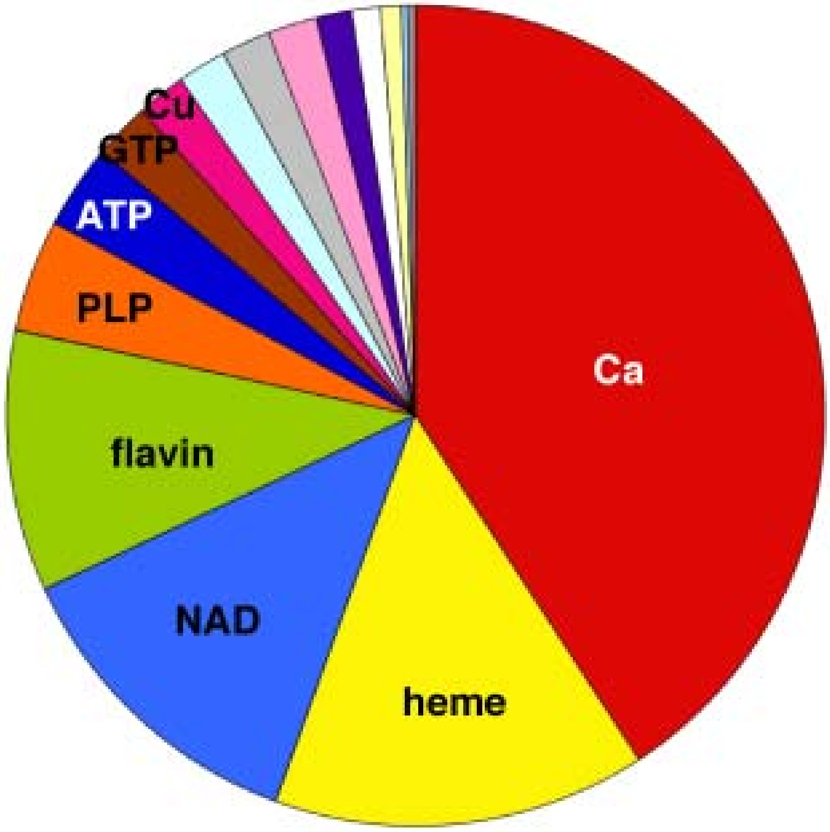

Figure Protocol S1. Assigning function to the H-Inv proteins using module 3-D keynotes.

We predicted the molecular functions of 350 proteins derived from Categories IV and V. The abbreviations on the above diagram indicate small molecules that have been predicted to bind to the proteins. Ca (calcium), NAD (nicotinamide adenine dinucleotide), PLP (pyridoxal 5'-phosphate), ATP/GTP (adenosine/guanosine 5'-triphosphate), and Cu (copper). Only the major molecules are explicitly named on the figure.

References

Go M. (1983) Modular structural units, exons, and function in chicken lysozyme. Proc. Natl. Acad. Sci. U.S.A. 80: 1964-1968.

Noguti T, Sakakibara H, Go M. (1993) Localization of hydrogen bonds within modules in barnase. Proteins: Struct. Funct. Genet. 16: 357-363.

Yura K, Toh H, Go M. (1999) Putative mechanism of natural transformation as deduced from genome data. DNA Res. 6: 75-82.

Yoshihara S, Geng X, Okamoto S, Yura K, Murata T. et al. (2001) Mutational analysis of genes involved in pilus structure, motility and transformation competency in the unicellular motile cyanobacterium Synechocystis sp. PCC 6803. Plant Cell Physiol 42: 63-73.

Bootman MD, Lipp P, Berridge, M. J. (2001) The organisation and functions of local Ca(2+) signals. J Cell Sci 114: 2213-2222.
